# Proteomic changes in the base of chrysanthemum cuttings during adventitious root formation

**DOI:** 10.1186/1471-2164-14-919

**Published:** 2013-12-26

**Authors:** Ruixia Liu, Sumei Chen, Jiafu Jiang, Lu Zhu, Chen Zheng, Shuang Han, Jing Gu, Jing Sun, Huiyun Li, Haibin Wang, Aiping Song, Fadi Chen

**Affiliations:** 1 College of Horticulture, Nanjing Agricultural University, Nanjing 210095, China

**Keywords:** Chrysanthemum, Anatomy, Adventitious root, Proteomics, Western blot

## Abstract

**Background:**

A lack of competence to form adventitious roots by cuttings of Chrysanthemum (*Chrysanthemum morifolium*) is an obstacle for the rapid fixation of elite genotypes. We performed a proteomic analysis of cutting bases of chrysanthemum cultivar ‘Jinba’ during adventitious root formation (ARF) in order to identify rooting ability associated protein and/or to get further insight into the molecular mechanisms controlling adventitious rooting.

**Results:**

The protein profiles during ARF were analyzed by comparing the 2-DE gels between 0-day-old (just severed from the stock plant) and 5-day-old cutting bases of chrysanthemum. A total of 69 differentially accumulated protein spots (two-fold change; t-test: 95% significance) were excised and analyzed using MALDI-TOF/TOF, among which 42 protein spots (assigned as 24 types of proteins and 7 unknown proteins) were confidently identified using the NCBI database. The results demonstrated that 19% proteins were related to carbohydrate and energy metabolism, 16% to photosynthesis, 10% to protein fate, 7% to plant defense, 6% to cell structure, 7% to hormone related, 3% to nitrate metabolism, 3% to lipid metabolism, 3% to ascorbate biosynthesis and 3% to RNA binding, 23% were unknown proteins. Twenty types of differentially accumulated proteins including ACC oxidase (CmACO) were further analyzed at the transcription level, most of which were in accordance with the results of 2-DE. Moreover, the protein abundance changes of CmACO are supported by western blot experiments. Ethylene evolution was higher during the ARF compared with day 0 after cutting, while silver nitrate, an inhibitor of ethylene synthesis, pretreatment delayed the ARF. It suggested that ACC oxidase plays an important role in ARF of chrysanthemum.

**Conclusions:**

The proteomic analysis of cutting bases of chrysanthemum allowed us to identify proteins whose expression was related to ARF. We identified auxin-induced protein PCNT115 and ACC oxidase positively or negatively correlated to ARF, respectively. Several other proteins related to carbohydrate and energy metabolism, protein degradation, photosynthetic and cell structure were also correlated to ARF. The induction of protein CmACO provide a strong case for ethylene as the immediate signal for ARF. This strongly suggests that the proteins we have identified will be valuable for further insight into the molecular mechanisms controlling ARF.

## Background

In dicotyledonous plants, adventitious roots can be defined as roots that develop from organs such as leaves and stems under unusual circumstances. Adventitious root formation (ARF) in leafy stem cuttings is a crucial physiological process for propagation of many ornamental plant species. Despite intensive control of environmental factors in the modern propagation industry, high economic losses still occur as a result of insufficient rooting [1]. Chrysanthemum (*Chrysanthemum morifolium*), a plant of high ornamental value and economic importance, also suffers insufficient rooting of leafy stem cuttings. Poor understanding of the mechanisms and the signals that control the development of adventitious root hampers the use of reliable technologies to improve ARF of cuttings of ornamental plants.

Adventitious rooting is known to be a quantitative genetic trait that is affected by multiple endogenous and environmental factors. One of the endogenous factors known to play a key role in the control of ARF is auxin. Numerous authors established that auxin had the ability to promote adventitious root. Pagnussat *et al.* demonstrate that nitric oxide mediates the auxin response leading the ARF in cucumber [2]. The progressive accumulation and local concentration of auxin in the base of the cuttings seems to be important for starting the rooting process [3]. Localized synthesis of ABCB19 protein leads to enhanced IAA transport and local accumulation of IAA which drives ARF [4]. Auxin-dependent ARF is possibly via H_2_O_2_– and Nitric oxide-dependent cGMP signaling in mung bean seedlings [5] or an involvement of crosstalk between the auxin and jasmonate regulatory pathways [6].

There is increasing evidence that ARF is also dependent on the action of ethylene [7,8], production of which is caused by wounding during the cutting process. The role of ethylene in the ARF has been examined in a variety of plant species, such as in *Pelargonium* cuttings, preharvest endogenous carbohydrate status interacts with postharvest ethylene action to regulate ARF [9]. Recent studies in tomato have also identified a positive role for ethylene in ARF with modulation of auxin transport as a central point of ethylene-auxin crosstalk [10]. Overexpression of *PtAIL1*, a transcription factor of the AP2, increased number of adventitious roots in Populus [11].

Wound responses associated with cutting excision are integrated and often necessary in the steps leading to adventitious root [12]. Once excised from the stock plant, cuttings need to redistribute their remaining resources as soon as possible to form adventitious roots and restore the balance of source and sink, which enables resources to be passed between different parts of the plant. There is evidence that carbohydrate allocation and distribution within the cutting could be more important than the content itself [13,14].

ARF in *Petunia hybrida* cuttings were defined as three metabolic phases, i.e., sink establishment phase, recovery phase, and maintenance phase [1]. During this complex process, many proteins work together to help the cutting survive. A proteomic analysis of different mutant genotypes of Arabidopsis led to the identification of 11 proteins correlating with adventitious root development [15]. 19 differentially accumulated proteins during shoot-born root were identified in maize via ESI MS/MS mass spectrometry [16]. However, how proteins play roles in ARF of chrysanthemum cuttings remained unknown. In present study, we described the analysis of 2-DE protein profiles, which is aimed to contribute to a better understanding of the mechanisms underlying ARF in chrysanthemum. Compared to 0-day-old cutting bases, 69 protein spots showed significant variation. 42 protein spots were identified by MALDI-TOF/TOF and assigned as 24 different types of protein of known function. 20 genes corresponding to these proteins were successfully cloned and analyzed by qRT-PCR. Most of gene expression profiles were in accordance with the protein pattern. Moreover, the changes in protein abundance of ACO during ARF are supported by western blot experiments. The present study gained a new insight on the proteins related to ARF of chrysanthemum.

## Results

### Anatomy of ARF in cutting bases of ‘Jinba’

**Figure 1:**
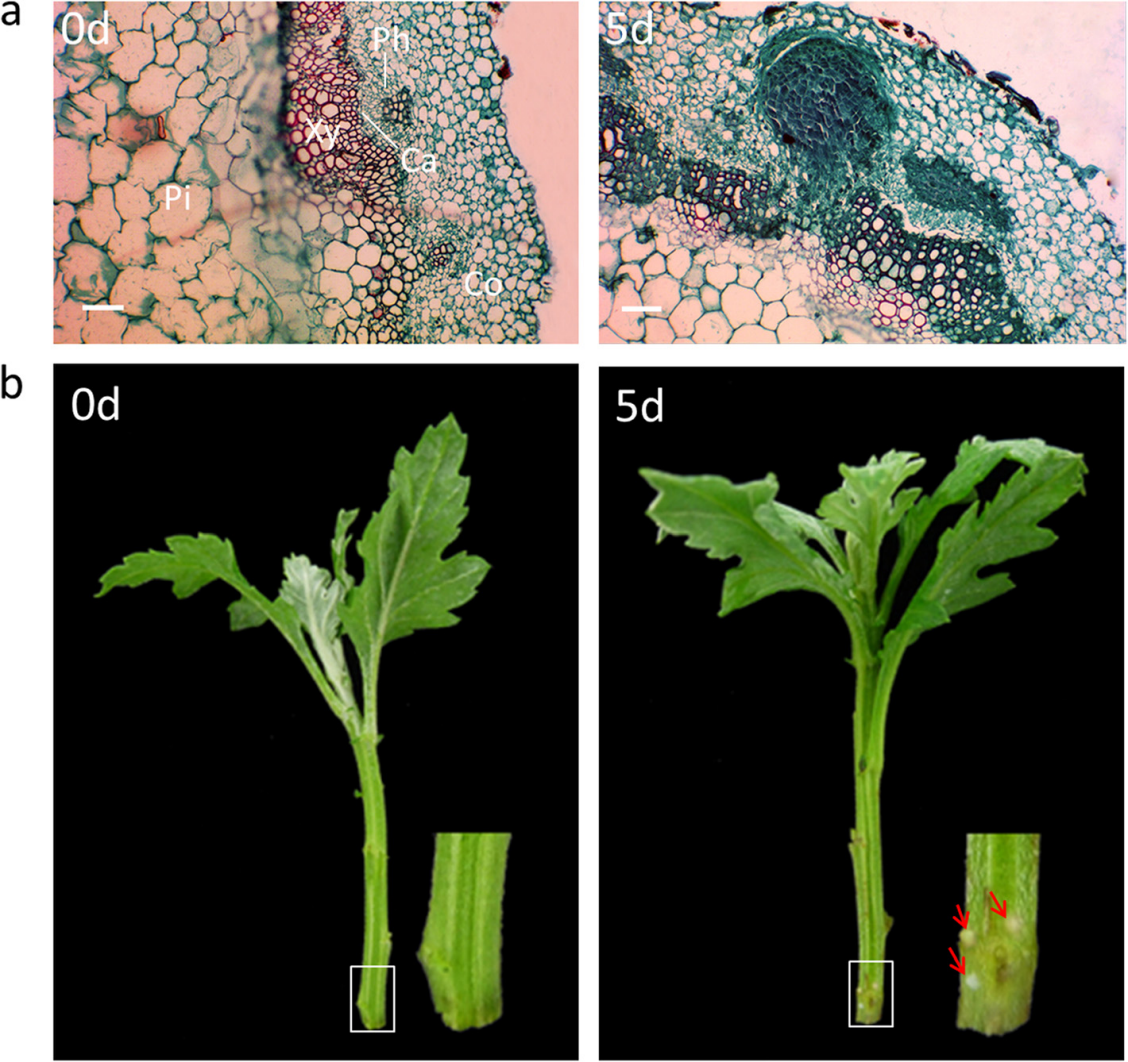
**Morphological and histological properties of adventitious root primodium formation in the stem base of chrysanthemum ‘Jinba’ cuttings.** Anatomy of adventitious root primodium formation **(a)** 0 d post excision, typical stem anatomy consists of the cortex (Co), the pith parenchyma (Pi) and a ring of vessels with phloem (Ph), cambium (Ca), and xylem (Xy) cells; 5 d after cutting, the first differentiated root primordia can be seen; Bars = 500 μm. Morphology of 0 d and 5 d cuttings **(b)**. The arrow indicated the adventitious root, zoomed image shows the cutting position.

Morphological and histological analyses were performed (Figure 1) to determine the time course of ARF in the cutting bases of chrysanthemum. Compared to 0-day-old cutting bases (Figure 1), in 5-day-old cutting bases some early adventitious root primordium with apical meristems and differentiation of the root body are visible under the microscope and from outside while adventitious roots are not yet initiated. For our subsequent proteomic analyses 5-day-old cutting bases have been chosen to compare the protein accumulation of 0-day-old cutting bases of chrysanthemum. In this way, proteins whose expression levels were changed during ARF could be identified.

### Analysis of 2-DE protein patterns of ARF

**Figure 2:**
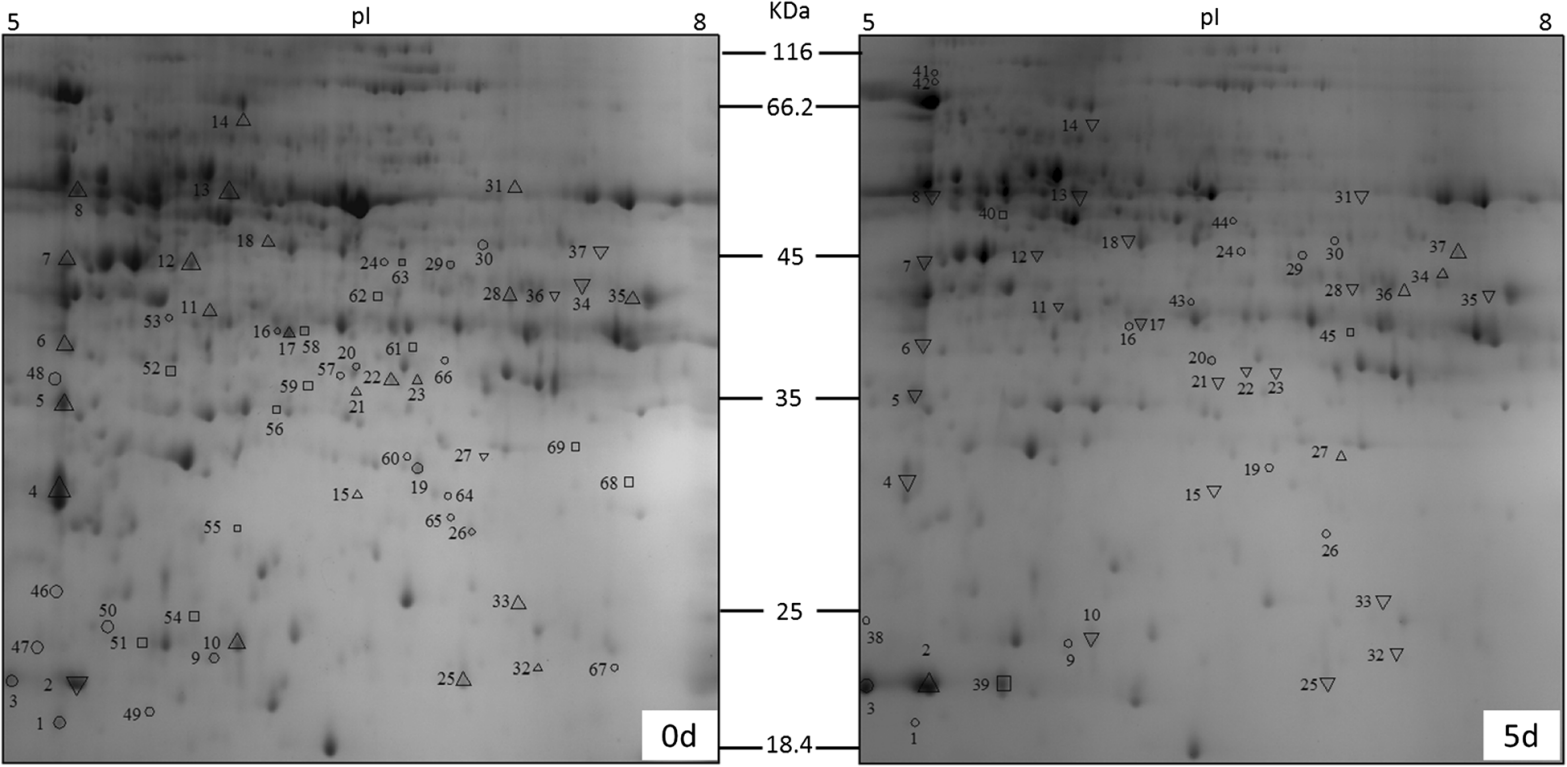
**Comparative proteomic 2-DE maps of soluble proteins extracted from 0 d and 5 d chrysanthemum cutting bases.** Each map depicts one representative gel (of three replicates). Proteins were separated in the first-dimension by their pIs on IPG strips pH 5–8 and in the second-dimension by their molecular masses on 12.5% SDS-polyacrylamide gels. Proteins were stained with colloidal Coomassie Blue R-350. A total of 69 protein spots showing differences between the 0 d and 5 d maps are numbered. Squares indicate spots that are only present at 0 d or 5 d. Triangles indicate spots that differed between 0 d and 5 d (up-regulated, up-pointing triangle; down-regulated, down-pointing triangle). Circles indicate spots that could not be identified by MALDI-TOF/TOF. Mass spectrometric identification of these proteins is summarized in Table 1.

After electrophoresis, the gels were stained with CBB R350 and analyzed using the PDQuest software (8.0.1). In three independent experiments, there were 604 and 530 protein spots in the 0 d and 5 d groups, respectively, mainly in the range of pH 5–8 and relative molecular mass 18–116 kDa. A total of 69 protein spots showed more than a 2-fold difference in expression values in 5-day-old cutting bases compared to the control 0-day-old ones (Figure 2). Of which 15 protein spots were up-regulated, 8 out of 15 protein spots were exclusively accumulated in the 5-day-old cutting bases. 54 protein spots were down-regulated, 24 out of 54 protein spots disappeared in the 5 d group.

### Identification of differentially accumulated proteins

All the 69 spots from the 2-DE gels (indicated in Figure 2) were cut out by hand and subjected to gel digestion using trypsin and MALDI-TOF/TOF analysis. We used automated the Mascot software to search the NCBI.nr database. 42 of the 69 differentially accumulated protein spots were confidently matched to NCBI database entries, but the remaining 27 protein spots were not confidently matched. This could be due to the lack of genomic information for the chrysanthemum. Table 1 lists the accession numbers, molecular weights and possible molecular functions of all proteins identified.

**Table 1:** MALDI-TOF/TOF identification of proteins from 0 d and 5 d cutting bases of chrysanthemum and the expression profiles of the corresponding genes

^**a**^**Spot number**	^**b**^**Accession number**	^**c**^**Species**	^**d**^**Protein description**	^**e**^**Abb.**	^**f**^**Observed Mr(kDa)/pI**	^**g**^**Specificity 5d: 0d**	^**h**^**Cov. (%)**
**Carbohydrate and energy metabolism associated proteins**							
**8**	gi|7708624	*Roussea simplex*	**ATP synthase beta subunit**	AtpB	50.0/5.4	−2.36^±0.36^	13
**51**	gi|6017842	*Simmondsia chinensis*	**ATP synthase beta subunit**	AtpB	44.1/5.8	0d only	11
**52**	gi|6017842	*Simmondsia chinensis*	**ATP synthase beta subunit**	AtpB	49.7/6.0	0d only	14
**68**	gi|6017842	*Simmondsia chinensis*	**ATP synthase beta subunit**	AtpB	33.8/7.0	0d only	11
**15**	gi|2497857	*Brassica napus*	**Malate dehydrogenase**	MDH	31.4/6.4	−3.67^±0.33^	4
**61**	gi|2497857	*Brassica napus*	**Malate dehydrogenase**	MDH	36.1/6.6	0d only	7
**18**	gi|1346698	*Spinacia oleracea*	**Phosphoglycerate kinase**	PGK	46.0/6.1	−3.33^±0.33^	3
**28**	gi|1766048	*Arabidopsis thaliana*	**NAD**^**+ **^**dependent isocitrate dehydrogenase subunit 2**	IDH	42.0/6.8	−3.57^±0.15^	5
**35**	gi|255575381	*Ricinus communis*	**Putative fructose-bisphosphate aldolase**	ALDOA	33.9/5.3	−3.03^±0.19^	3
**37**	gi|169039	*Pisum sativum*	**Aldolase**	ALD	43.8/5.4	+3.33^±0.33^	8
**39**	gi|169039	*Pisum sativum*	**Aldolase**	ALD	50.0/5.4	5d only	10
**58**	gi|169039	*Pisum sativum*	**Aldolase**	ALD	46.0/6.1	0d only	10
**63**	gi|169039	*Pisum sativum*	**Aldolase**	ALD	20.8/6.9	0d only	10
**N assimilation and metabolism**							
**36**	gi|152962665	*Lactuca sativa*	**Glutamate dehydrogenase**	GDH	37.6/5.4	+2.70^±0.41^	6
**Lipid metabolism**							
**62**	gi|134944	*Carthamus tinctorius*	**Stearoyl-ACP desaturase**	S-ACP-DES	35.9/6.7	0d only	7
**Photosynthesis**							
**4**	gi|6716783	*Euphorbia esula*	**Chlorophyll a/b binding protein precursor**	CAB	28.5/5.3	−7.26^±1.85^	8
**12**	gi|100380	*Nicotiana tabacum*	**Ribulose-bisphosphate carboxylase activase**	RCA	44.1/5.8	−5.33^±0.19^	7
**25**	gi|100380	*Nicotiana tabacum*	**Ribulose-bisphosphate carboxylase activase**	RCA	20.8/6.9	−2.88^±0.31^	7
**32**	gi|100380	*Nicotiana tabacum*	**Ribulose-bisphosphate carboxylase activase**	RCA	21.1/7.2	−3.00^±0.17^	5
**31**	gi|47604692	*Freycinetia formosana*	**RuBisCO large subunit**	RbcL	50.1/6.9	−2.67^±0.67^	5
**56**	gi|4754913	*Gossypium hirsutum*	**Carbonic anhydrase isoform 1**	CA1	39.4/6.2	0d only	5
**59**	gi|15234905	*Arabidopsis thaliana*	**Magnesium-protoporphyrin IX methyltransferase**	CHLM	35.3/6.4	0d only	3
**Hormone related**							
**6**	gi|222051629	*Lactuca sativa*	**ACC oxidase 1**	ACO	37.6/5.4	−2.31^±0.68^	4
**45**	gi|728744	*Nicotiana tabacum*	**Auxin-induced protein PCNT115**	PCNT115	40.5/5.9	5d only	4
**Plant defense**							
**7**	gi|255563252	*Ricinus communis*	**Disease resistance protein RPS5, putative**	RPS5	43.8/5.4	−2.78^±0.40^	0
**33**	gi|77744871	*Populus balsamifera*	**Temperature-induced lipocalin**	TIL	20.7/5.4	−3.46^±0.288^	9
**34**	gi|77744871	*Populus balsamifera*	**Temperature-induced lipocalin**	TIL	28.5/5.3	+4.00^±0.58^	9
**Ascorbate biosynthesis**							
**17**	gi|15241945	*Arabidopsis thaliana*	**GDP-mannose 3,5-epimerase**	GME	39.4/6.2	−2.60^±0.21^	7
**Cell structure**							
**23**	gi|82394883	*Gerbera hybrid cultivar*	**Xyloglucan endotransglucosylase**	XTH	35.9/6.7	−4.00^±1.00^	5
**54**	gi|17366765	*Petunia hybrida*	**Actin-depolymerizing factor 2**	ADF2	64.7/6.0	5d only	8
**Protein fate**							
**10**	gi|33325121	*Hevea brasiliensis*	**Eukaryotic translation initiation factor 5A isoform III**	eIF-5A	21.9/5.9	−3.96^±0.44^	18
**13**	gi|1709798	*Solanum tuberosum*	**26S protease regulatory subunit 6B homolog**	PRS6B	49.7/6.0	−3.43^±0.37^	4
**14**	gi|1709798	*Solanum tuberosum*	**26S protease regulatory subunit 6B homolog**	PRS6B	64.7/6.0	−2.46^±0.83^	4
**40**	gi|1155261	*Arabidopsis thaliana*	**Eukaryotic release factor 1 homolog**	eRF1	21.9/5.9	5d only	2
**2**	gi|46806499	*Oryza sativa Japonica Group*	**Putative heterogeneous nuclearribonucleoprotein A2**	hnRNPA2	20.7/5.4	+2.44^±0.43^	4
**Unknown**							
**5**	gi|168065628	*Physcomitrella patens subsp. patens*	**Predicted protein**		33.9/5.3	−2.49^±0.19^	7
**11**	gi|168044242	*Physcomitrella patens subsp. patens*	**Predicted protein**		40.5/5.9	−2.67^±0.44^	1
**21**	gi|224111100	*Populus trichocarpa*	**Predicted protein**		35.3/6.4	−2.19^±0.31^	10
**22**	gi|326530532	*Hordeum vulgare subsp. vulgare*	**Predicted protein**		36.1/6.6	−2.50^±0.50^	3
**27**	gi|224111100	*Populus trichocarpa*	**Predicted protein**		33.8/7.0	+3.88^±0.60^	10
**55**	gi|224116012	*Populus trichocarpa*	**Predicted protein**		31.4/6.4	0d only	15
**69**	gi|302852099	*Volvox carteri f. nagariensis*	**Hypothetical protein VOLCADRAFT_98675**		42.0/6.8	0d only	0

Proteins were considered to be differentially expressed if they showed at least a two-fold difference between the 0 and 5 d gels at *p* < 0.05 in t-tests of three biological replicates.

^a^Numbering corresponds to the 2-DE gels shown in Figure 2.

^b^Accession number of the homologous proteins obtained via the MASCOT software from the NCBI.nr database.

^c^Species of the homologous proteins obtained via the MASCOT software from the NCBI.nr database.

^d^Names of the homologous proteins obtained via the MASCOT software from the NCBI.nr database.

^e^Abbreviation of the homologous proteins from the NCBI.nr database.

^f^Mr and pI of the protein spots on gel calculated with PDQuest software.

^g^Specificity indicates the ratio of accumulation of a particular protein between 5-day-old cutting bases versus 0-day-old cutting bases protein preparations. A plus sign indicate spots that up-regulated. A minus sign indicate spots that down-regulated. Each value represents the mean of three independent replicates ± SE. ^h^Percentage of predicted protein sequence covered by matched peptides.

### Functional annotation of the identified proteins

For the 42 protein spots identified via the annotated NCBInr database (Table 1, Figure 2) a function could be immediately predicted. The proteins identified were assigned as 31 different types of protein, including 24 known proteins and 7 unknown proteins. Since spots 8, 51, 52 and 68 have been identified as ATP synthase beta subunit (AtpB). Spots 15 and 61 as malate dehydrogenase (MDH). Spots 37, 39, 58 and 63 as aldolase (ALD). Spots 12, 25 and 32 as ribulose-bisphosphate carboxylase activase (RCA). Spots 33 and 34 as temperature-induced lipocalin (TIL). Spots 13 and 14 as 26 S protease regulatory subunit 6 B homolog (PRS6B). This could be due to post-translational modifications of the same gene product as one protein was found in different locations and very abundant proteins cannot be clearly separated, or that a protein can have different splice variants.

Of the 24 proteins with known function, only 6 proteins were positively correlated with ARF, including putative heterogeneous nuclearribonucleoprotein A2 (spot 2, hnRNPA2), a predicted protein (spot 27), glutamate dehydrogenase (spot 36, GDH), and auxin-induced protein PCNT 115 (spot 45, PCNT115), protein TIL (spot 34) and ALD (spots 37 and 39).

The ALD protein (spots 37 and 39) out of the 6 proteins related to energy and carbon metabolism (19% of the identified proteins) were positively correlated with ARF. Spot 39 (ALD) was only appeared in 5-day-old cutting bases, and spot 37 (ALD) was up-regulated in 5-day-old cutting bases. ALD may play an important role during ARF in cutting bases of chrysanthemum.

GDH (spot 36) related to nitrate assimilation and metabolism (3% of the identified proteins), was up-regulated in 5-day-old cutting bases. GDH contribute to both carbon skeleton supply (2-oxoglutarate) and ammonium assimilation in plants, and also plays an important role in metabolic acclimation of tobacco roots to boron deprivation [17]. Thus, GDH is an important protein in meeting the nitrogen demand of cells during root formation in cuttings of chrysanthemum.

hnRNPA2 (spot 2), which was linked to RNA binding (3% of the identified proteins), up-regulated in 5-day-old cutting bases, has been reported that transcriptionally regulates smooth muscle cell differentiation gene expression and promotes neural crest cell migration and differentiation toward smooth muscle cells [18]. However, there has been no report of the functional involvement of hnRNPA2 in plant differentiation and development. In this study, we identified a potential role for hnRNPA2 in ARF from chrysanthemum cutting bases.

Adventitious root development in the cutting base of chrysanthemum involves the induction and repression of numerous genes in conjunction with changes in the levels of phytohormones (7% of the identified proteins). Here we found changes in the expression of ACO (spot 6) and auxin-induced protein PCNT115 (spot 45) in ARF.

Moreover, one of the interesting features was 5 proteins linked to photosynthesis (16% of the identified proteins) were negatively correlated with ARF. Chlorophyll a/b binding protein precursor (spot 4, CAB), RCA (spots 12, 25, 32) and RuBisCO large subunit(spot 31, RbcL) were down-regulated, while carbonic anhydrase isoform 1 (spot 56, CA1) and magnesium-protoporphyrin IX methyltransferase (spot 59, CHLM), an enzyme in the chlorophyll biosynthetic pathway [19], disappeared in the 5-day-old cutting bases during ARF.

Three proteins linked to protein fate (10% of the identified proteins) may regulate the protein degradation in ARF. Eukaryotic translation initiation factor 5A isoform III (spot 10, eIF-5A) and PRS6B (spot 13,14) were down-regulated at 5-day-old cutting bases, suggesting that protein synthesis was decreased, and Eukaryotic release factor 1 homolog (spot 40, eRF1) was only accumulated on the 5-day-old cutting bases, which is consistent with the protein degradation during ARF.

Some protein negatively correlated with ARF also could be identified. Two proteins involved in stress response and defense (7% of the identified proteins): disease resistance protein RPS5 (spot 7, RPS5) and TIL (spot 33). ARF of cuttings can be improved via conditioning of the donor plant, by application of arbuscular mycorrhizal fungi [20]. RPS5 declined during ARF may good for pathogen infection which will help the development of adventitious roots of chrysanthemum.

One protein related to ascorbate biosynthesis (3% of the identified proteins), GDP-mannose 3,5-epimerase (spot 17, GME). GME constitutes a control point for regulation of the ascorbate pathway in plants [21,22]. A possible control of root elongation by ascorbate via its action on peroxidases that are involved in the regulation of cell-wall extensibility [23]. The reason of down-regulation of GME on 5-day-old cutting bases may be that synthesis of ascorbic acid needs GME.

One protein related to lipid metabolism (3% of the identified proteins), Stearoyl-ACP desaturase (spot 62, S-ACP-DES). Two proteins involved in cell-wall structure (6% of the identified proteins): xyloglucan endotransglucosylase (spot 23, XTH) and actin depolymerizing factor (spot 54, ADF2).

**Figure 3:**
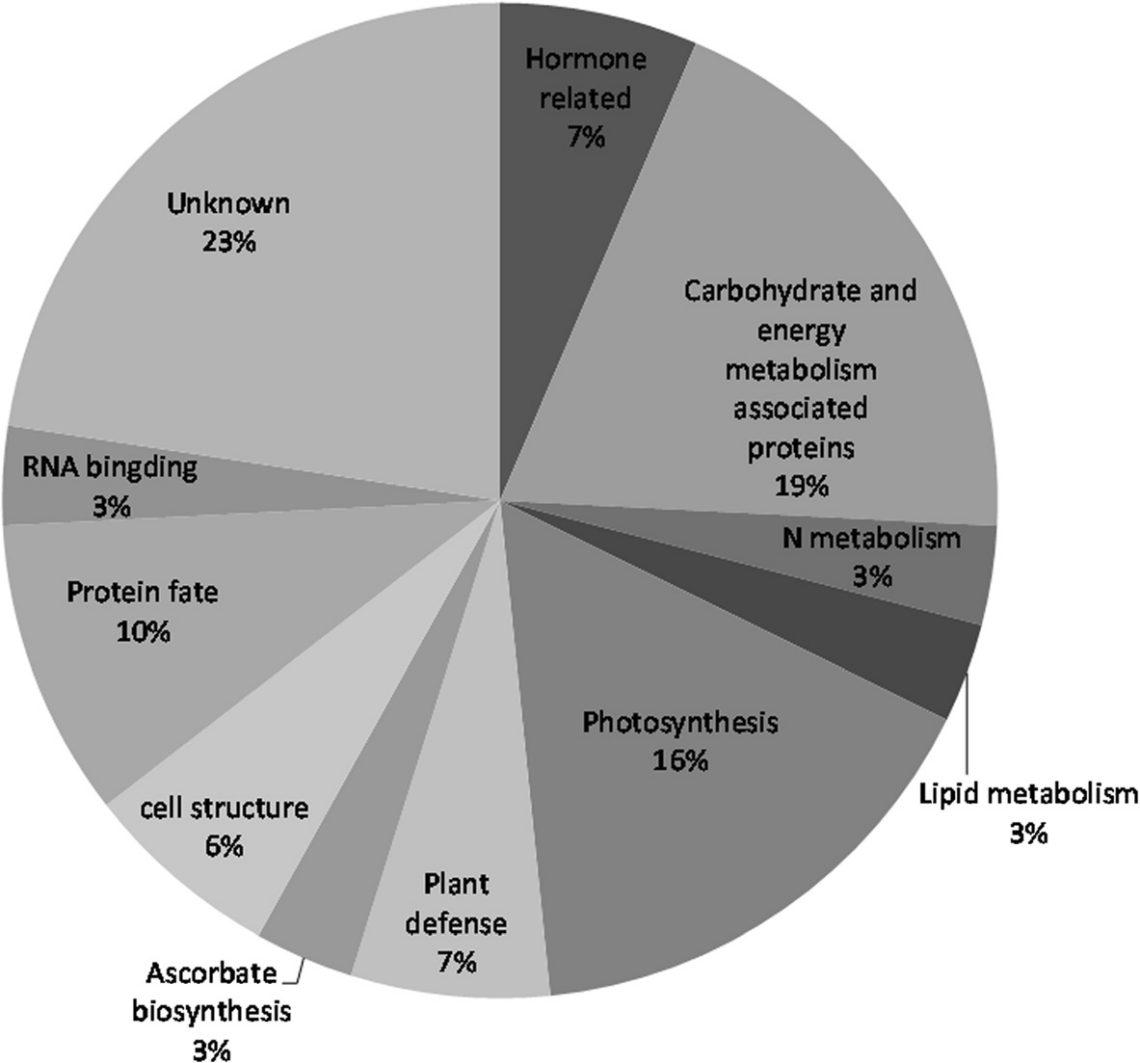
**The functional classification and distribution of all 42 identified proteins from cutting bases of chrysanthemum.** Unknown proteins include those whose functions have not been described. This classification is based on a BLAST search and their homologies and literature.

The proteins identified in this study fell into eleven functional categories (Figure 3). The most abundant category of proteins at 23% was proteins that matched entries in the database with as yet unknown function. As it can be seen in Figure 3, of the proteins with known function, the most striking feature of this classification was the highest representation of proteins linked with carbohydrate and energy metabolism, which was in good accordance with Sorin’s study [15].

### Analysis of the identified proteins at the mRNA level

To confirm the protein expression changes during ARF observed in the 2-DE gels, we checked their corresponding gene expression levels at 0-day-old and 5-day-old cutting bases of chrysanthemum using qRT-PCR.

**Figure 4:**
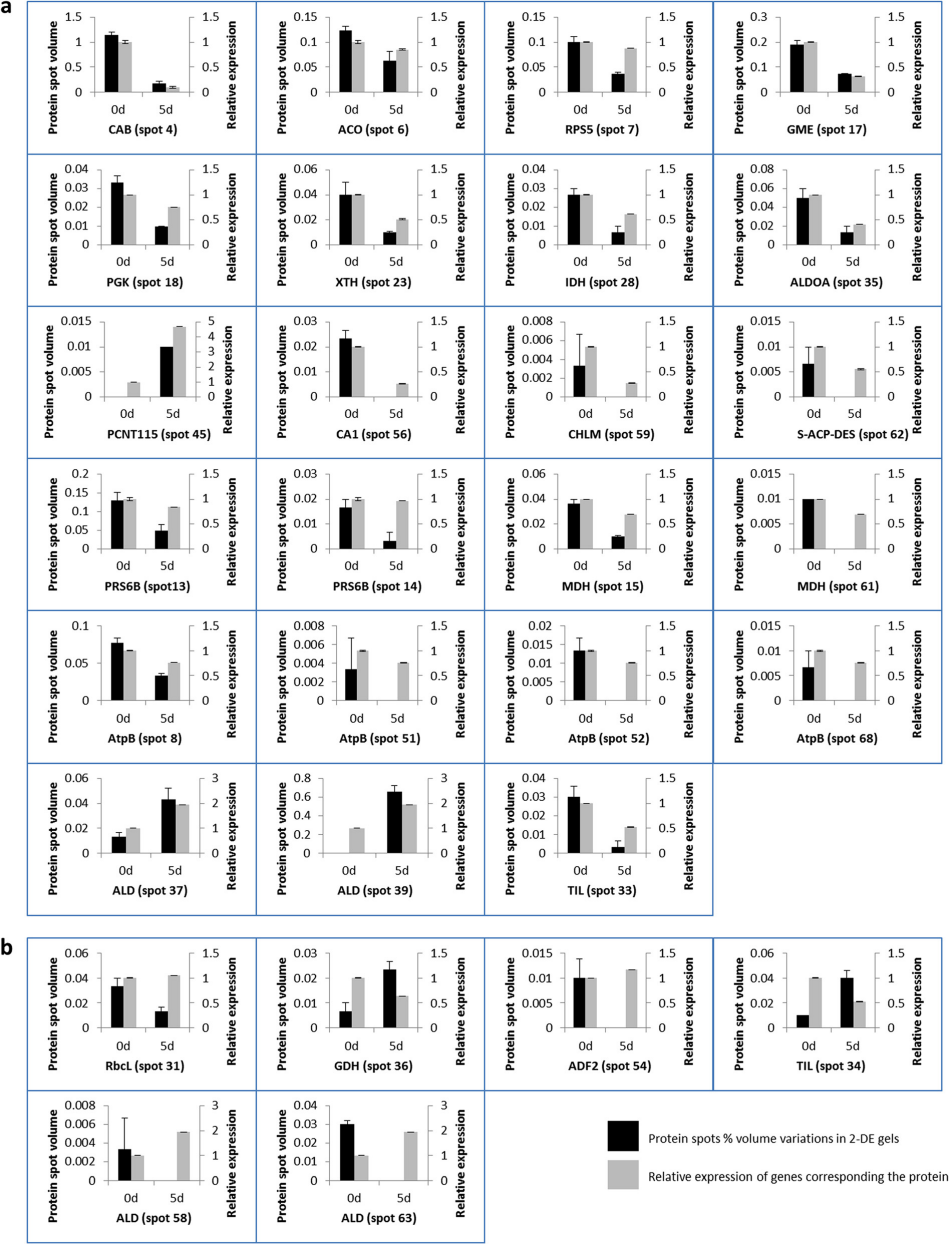
**Expression of confidently identified protein and its corresponding genes. (a)**, relative expression of genes are corresponding to successful identified protein expression patterns; **(b)**, relative expression of genes are different from the protein expression patterns. Each value represents the mean of three independent replicates ± SE.

The 42 identified protein spots were assigned as 31 different types of protein. Eleven proteins including four known proteins (eIF-5A, spot 10; eRF1, spot 40; hnRNPA2, spot 2; RCA, spots 12, 25 and 32) and 7 unknown proteins were not successfully cloned. Twenty genes (Additional file 1) corresponding to these proteins were cloned successfully according to our EST database (unpublished data), and their expression patterns at 0 and 5-day-old cutting bases were investigated at the mRNA level using qRT-PCR. The transcripts for the 23 spots (Figure 4a) were in accordance with the protein pattern, while the mRNA levels for 6 protein spots (Figure 4b, spot 31, 36, 54, 34, 58 and 63) were different from the protein pattern. For example, the levels of spot 37 and 39 of ALD (Figure 4a) were in accordance with the corresponding gene expression levels, while levels of spot 58 and 63 of ALD protein (Figure 4b) are contrast to their mRNA levels. TIL protein, for spot 33, the transcript level is consistent with the protein expression patterns of spot 33 (Figure 4a), but not for the spot 34 (Figure 4b). One possible explanation for this discrepancy between protein patterns and mRNA levels could be that there may be post-transcriptional changes in those proteins in the chrysanthemum roots projecting epidermis process.

### Expression level of *CmACO* during ARF

**Figure 5:**
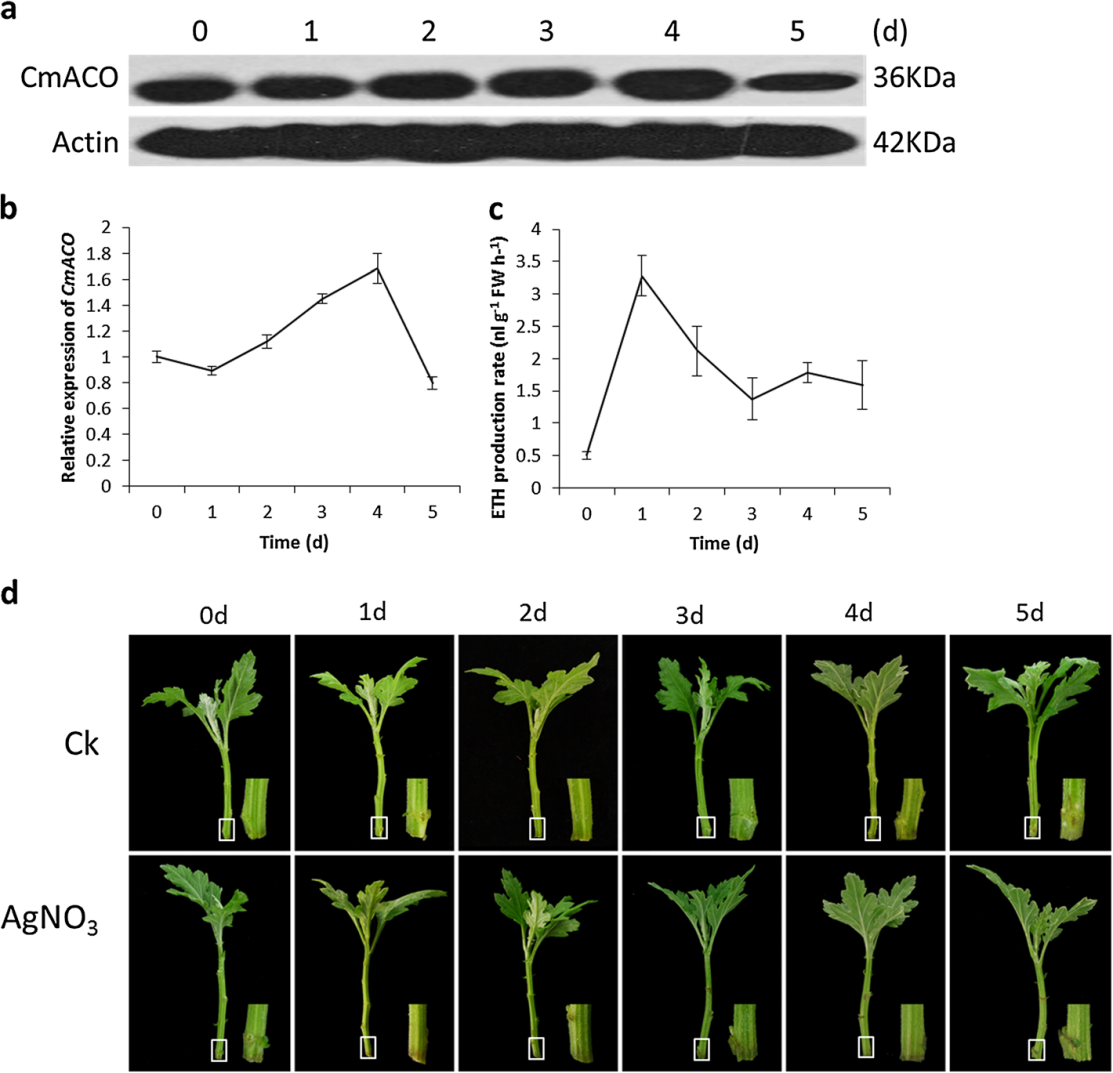
**CmACO transcript and protein expression in chrysanthemum cutting bases during adventitious root formation. (a)**, CmACO protein expression in the cutting bases of chrysanthemum were detected by Western blotting using antiACO-monoclonal antibody; **(b)**, *CmACO* transcript was analyzed by qRT-PCR. Each value represents the mean of three independent replicates ± SE. **(c)**, ethylene production during adventitious root formation in chrysanthemum cutting bases. Each value represents the mean of three independent replicates ± SE; **(d)**, morphological changes during adventitious root formation and AgNO_3_ inhibits adventitious root formation.

Treatment with the ethylene precursor 1-aminocyclopropane carboxylic acid reduced lateral root formation in Arabidopsis [24], *CmACO* was of particular interest. To determine *CmACO* expression level and protein abundance during ARF in the cutting bases of chrysanthemum, we used qRT-PCR and western blot technique. We established a new monoclonal antibody against the CmACO (date unpublished) and used it for the Western blot detection. At the transcript level, *CmACO* expression was slightly decreased in 1-day-old cutting bases, then gradually increased from 1 to 4-day-old cutting bases, while with a rapid declined at 5-day-old cutting bases (Figure 5b). Western blots demonstrated that the monoclonal antibody to CmACO recognized ACC oxidase from 0 d to 5-day-old cutting bases, showing a single band of the expected molecular mass of approximately 36 kD (Figure 5a). CmACO showed an increase in the amount of protein from 0 to 4-day-old cutting bases, followed by a subsequent decrease at 5-day-old cutting bases. The expression of *CmACO* on the level of mRNA and protein is strictly linear except that in 1-day-old cutting bases. Compared with the control (0-day-old cutting bases), the level of mRNA is down-regulated but the level of protein is up-regulated in 1-day-old cutting bases. Moreover, ethylene production during ARF in chrysanthemum cutting bases was detected by gas chromatography. Ethylene evolution was much higher than that of day 0. A climactic peak was observed on 1-day-old cutting bases followed by a decrease till day 3 after cutting, rose again after day 4, then decreased gradually on day 5 (Figure 5c). Ethylene synthesis inhibitor, AgNO_3_, inhibited the ARF of chrysanthemum (Figure 5d). All these data suggest that CmACO is involved in ARF of chrysanthemum.

## Discussion

2-DE analysis confirmed that a number of proteins were correlated to ARF, which is similar to the observations in Arabidopsis and maize [15,16]. We could find some overlap among the protein identified here and the proteins described to be potentially associated with ARF in Arabidopsis [15]. This is the case of the CA1 (spot 56), the ACO (spot 6), the ALDOA (spot 35) and the IDH (spot 28). We have shown that grafting improved rooting ability of chrysanthemum by altering several physiological aspects [25].

### Carbohydrate and energy metabolism

Respiration is the most important postharvest physiological process, so the fact that 19% of the proteins identified during the process of rooting are energy related is not surprising. Among which, only ALD (spot 37 and 39) positively correlated to the ARF. Whereas, AtpB (spot 8, 51, 52 and 68), MDH (spot 15 and 61), PGK (spot 18), IDH (spot 28) and ALDOA (spot 35) were down-regulated.

Interestingly, agreement with study in Arabidopsis, the ALDOA that was recently proposed to be negatively correlated to adventitious root number as well as to the free IAA content, and phosphoribulokinase were negatively correlated to adventitious root number [15]. The expression of ALD, which may regulate the vacuolar H^+^-ATPase mediated control of cell elongation that determines root length [26]. Evidence showed an important role of the TCA cycle in the coordination of photosynthetic and respiratory metabolisms of the illuminated leaf [27], the possible role of IDH, a TCA cycle enzyme [28], may play an important role in resources redistribution during ARF.

AtpB was identified as a novel plant cell death regulator in Arabidopsis [29]. Programmed epidermal cell death induced by ethylene occurred at the site of adventitious root emergence in rice [30]. Thus, whether a decrease in AtpB (spots 8, 51, 52 and 68) triggered the cell death during ARF of chrysanthemum remained to be studied.

### Photosynthetic

The down-regulation of some photosynthetic proteins is regulated by light. For example, CA is regulated by light at the mRNA level [31], and there are interactions between mitochondrial metabolism and photosynthetic carbon assimilation [32]. Consequently, the down-regulation of proteins linked to photosynthesis and to the TCA cycle (such as MDH, spots 15 and 61) fits with the light hypersensitivity of ARF.

### Protein fate

eRF1 is responsible for the recognition of stop codons in mRNAs during protein synthesis, but accumulating evidence indicates that eRF1 functions in other processes in addition to translation termination. Expression pattern of eRF1(spot 40) and CAB (spot 4) in ARF of chrysanthemum is in agreement with overexpressing eRF1 in *Arabidopsis* during germination and early seedling development was accompanied by a dramatic reduction of CAB [33]. This is reliable evidence to the 2-DE protein profiles of ARF in chrysanthemum.

### Hormone-related proteins

Given the complexity role of ethylene in the rooting process, we are interested in the detailed mechanisms of ACO (which catalyzes the last step of ethylene biosynthesis) in the rooting process. Adding ACC to the rooting medium significantly inhibits rooting percentage [34]. Some researchers have found that enhanced ethylene synthesis promoted the initiation of lateral root primordial [35]. The wound-induced increase in ethylene, seen within 3 h of production of the cuttings, is a key stimulatory factor in the formation of root primordial [36]. In mung bean hypocotyl cuttings, ethylene stimulated rooting by enhancing the increase in auxins [37]. Applied ACC both increased ARF on vegetative stem cuttings from ethylene-insensitive *Never ripe* tomato and wild-type plants, but *Never ripe* tomato cuttings produced fewer adventitious roots than wild-type cuttings [7]. Although a Pearson correlation analysis indicated that ACO non-significantly varied with ARF in Arabidopsis [15]. But in chrysanthemum, CmACO is highly induced till early adventitious root appeared, the ethylene evolution were higher than that on day 0 after cuttings. AgNO_3_ inhibited *in vitro* rooting in sweet orange indicating that ethylene was important for rhizogenesis [38]. Similarly, the ethylene synthesis inhibitor, AgNO_3_, inhibited the ARF of chrysanthemum. In addition, our previous study showed that waterlogging triggers ethylene evolution, which in turn results in ARF [39]. These data suggests that the function of ACO is at least partially conserved among different plant species, and ethylene did contribute to the ARF of chrysanthemum.

An interplay between ethylene and auxin in the process of ARF in waterlogged tobacco, most likely on the level of polar auxin transport [40]. A higher auxin concentration was required for the induction phase of adventitious root but inhibited the formation phase in which anatomical changes take place [12]. We observed that auxin-induced protein PCNT115 (spot 45) was present only in 5-day-old cutting bases and the transcript displayed higher expression correspondingly on day 5. The auxin-induced protein PCNT115 is thought to belong to the aldo and keto reductase (AKR) family 2 [41]. GmAKR1 in soybean showed a root-specific expression pattern and was inducible by the synthetic auxin analogue 2,4-D, which appeared to be corroborated by the presence of root-specific and stress-response elements in its promoter region [42]. It inferred that Auxin-induced protein PCNT115 is the most likely protein governing the formation of new roots in chrysanthemum. It will be our ongoing research topic.

## Conclusion

The proteomic analysis of cutting bases of chrysanthemum allowed us to identify proteins whose expression was related to ARF. We identified auxin-induced protein PCNT115 and CmACO positively or negatively correlated to ARF, respectively. Several other proteins related to carbohydrate and energy metabolism, protein degradation, photosynthetic and cell structure were also correlated to ARF. The induction of protein CmACO provide a strong case for ethylene as the immediate signal for ARF. This strongly suggests that the proteins we have identified will be valuable for further insight into the molecular mechanisms controlling ARF.

## Methods

### Plant materials, growth and harvesting conditions

Chrysanthemum cultivar ‘Jinba’ plants were grown in the Chrysanthemum Germplasm Resource Preserving Centre, Nanjing Agricultural University, China. To avoid the effects of artificial conditions, e.g., agar substrate and exogenous hormones, which might lead to a misinterpretation of the pathways that are modulated during ARF, excised leafy cuttings of chrysanthemum ‘Jinba’ harboring four to five leaves of similar size were transferred to perlite and vermiculite (1:1) as a neutral substrate without any external additives. Trays containing cuttings were covered to maintain a humid environment, and put in a greenhouse (day/night temperature 25/18°C, photoperiod 16 h, light intensity 50 μmol m^−2^ s^−1^, relative humidity 70%). At 0-day-old, 1-day-old, 2-day-old, 3-day-old, 4-day-old and 5-day-old cutting bases of ARF, 8 mm in length samples of each cutting base (the rooting zone) were immediately frozen in liquid N_2_ and stored at −80°C or fixed in a solution of formalin–alcohol–glacial acetic acid (90:5:5 by volume) for anatomical investigation. Roots emerged after 5 d from the first 1 cm of the cutting stem base. All analyses were therefore carried out within 5 d of excision.

### Anatomical investigation of cutting bases

Samples were taken from the cutting base, cut into ~0.5 cm segments, and fixed in formalin–alcohol–glacial acetic acid (90:5:5 by volume) for at least 24 h. Stem segments were dehydrated through an alcohol series, infiltrated with xylene, and embedded in paraffin wax [43]. Transverse 8 μm thick sections were obtained using a rotary microtome (RM2016, China), double stained with Safranin/Fast Green, mounted in Canada balsam and photographed with an Olympus Bx40 microscope (Olympus Optical, Tokyo, Japan).

### Protein extraction

Protein extraction was performed using the phenol protocol [44] with some modifications. The samples of frozen chrysanthemum cutting bases were finely powdered in a mortar with quartz and liquid nitrogen. Then, 6 mL of TCA/acetone was added for homogenization and the solution was centrifuged at 13,500 g for 10 min at 4°C. The supernatant was discarded, and the precipitate was washed two to three times with cold 80% acetone and centrifuged again at 13,500 g at 4°C for 10 min. Next, 1 mL of 1% SDS was added to dissolve the precipitate on ice, followed by centrifugation at 13,500 g at 4°C for 10 min. The supernatant was then transferred to a fresh tube. An equal volume of Tris–phenol was added to the supernatant before centrifugation at 13,500 g at 4°C for 10 min. Five volumes of 0.1 M ammonium acetate/methanol solution were added and gently mixed with the transferred upper phenol phase before incubation for overnight at −20°C. The phenol phase was then centrifuged at 13,500 g at 4°C for 10 min, and the precipitate was washed twice with cold 80% acetone. After air-drying, the precipitate was dissolved in hydration solution [7 M urea, 2 M thiourea, 4% (w/v) CHAPS, 1% (w/v) DTT and 0.5% (v/v)]. The protein content was determined colorimetrically according to the Bradford method [45], using bovine serum albumin as a standard. The protein samples were stored at −20°C for further analysis.

### 2-DE and staining

Sample aliquots containing 1200 μg of protein were applied to 17 cm pH 5–8 IPG strips, and small volumes of lysis buffer (hydration solution and 0.5% (v/v) pH 5–8 IPG buffer) were added to the sample aliquots to achieve a final volume of 350 μL. After 12 h in gel rehydration, isoelectric focusing was performed on a PROTEAN IEF system (Bio-Rad) for a total of 76 kVh at 20°C. The voltage was set at 100 V for 1 h, 200 V for 1 h, 500 V for 1 h, 1000 V for 1 h, 4000 V for 2 h, slow mode ramped to 8000 V over 2 h and then run at 8000 V until the final volt-hours (76 kVh) were reached. The strips were then equilibrated for 15 min in 2% (w/v) DTT in equilibration buffer (50 mM Tris–HCl pH 8.8, 6 M urea, 20% (v/v) glycerol and 2% (w/v) SDS) followed by 15 min in 2.5% (w/v) iodoacetamide in equilibration buffer. After equilibration the strips were sealed with 0.5% molten agarose in running buffer on 12% home-made gels and run on the Ettan Six vertical set (GE Healthcare) in a buffer of 25 mM Tris, 192 mM glycine, 0.1% SDS, at 15°C with a cooling device (GE Healthcare). The gels were run at 1 W/gel for 1.5 h, and then at 15 W/gel for 4 h. Staining was performed by placing the gels into fixative solution (40% ethanol, 10% acetic acid) for 2 h and then staining them with 0.175% Coomassie brilliant blue (CBB) R350 (GE Healthcare). This assay was repeated at least three times.

### Image acquisition and data analysis

The CBB R350-stained 2-DE gels were imaged with a Versdoc 4000 scanner (Bio-Rad), and the spot patterns were characterized using the PDQuest software (ver. 8.0.1, Bio-Rad). The images were properly cropped and optimized, and then gel-to-gel matching of the standard protein maps was performed. The spot detection parameters were optimized by checking different protein spots in certain regions of the gel and then automatically detected, followed by visual inspection for removal or addition of undetected spots. Spot detection was refined by manual spot editing when needed. The spots that were present on at least two gels of one treatment or control based on the image analysis were identified as expressed protein spots. The abundance of each protein spot was estimated by the percentage volume (vol.%), i.e., the spot volumes were normalized as a percentage of the total volume for all the spots present in the gel to correct variability because of loading, gel staining, and destaining. The percentage volumes were used to designate the significant differentially expressed spots (at least two-fold increase/decrease and statistically significant as calculated by one-way ANOVA, *P* < 0.05). Only those with reproducible and significant changes were considered to be differentially expressed proteins.

### Protein in-gel digestion

Spots showing statistically significant changes (at *P* < 0.05) above a 2-fold threshold or only present on either 0 d or 5 d gels were excised, washed with double-distilled water and transferred to clean tubes. The protein spots were then washed with 25 mmol L^−1^ NH_4_HCO_3_, followed by dehydration with 50% (v/v) acetonitrile (ACN) in 25 mmol L^−1^ NH_4_HCO_3_. The proteins therein were then reduced with 10 mmol L^−1^ DTT in 50 mmol L^−1^ NH_4_HCO_3_ for 1 h at 56°C and alkylated in 55 mmol L^−1^ iodoacetamide in 50 mmol L^−1^ NH_4_HCO_3_ for 1 h at room temperature. The gel pieces were washed several times with 50 mmol L^−1^ NH_4_HCO_3_ followed by dehydration with ACN and finally dried in a vacuum centrifuge. The proteins were digested overnight at 37°C by the addition of 15 mL of trypsin (Promega, USA, 12.5 ng mL^−1^ in 25 mmol L^−1^ NH_4_HCO_3_). The resulting peptides were extracted by washing the gel pieces with 0.1% trifluoroacetic acid in 67% ACN. The supernatants were gathered and stored at −20°C until analysis.

### Protein identification by MALDI-TOF/TOF and database searches

Samples were air-dried and analyzed with an ultraflex TOF/TOF Proteomics Analyzer (Bruker). The UV laser was operated at a 200 Hz repetition rate with a wavelength of 355 nm. The accelerated voltage was set at 20 kV. Protein digested by trypsin was used to calibrate the mass instrument using the internal calibration mode. Parent mass peaks with a mass range of 800–4000 Da and minimum S/N 20 were picked out for tandem TOF/TOF analysis. A combined search (MS plus MS/MS) was performed using the GPS Explorer™ software v3.5 (Applied Biosystems) over the NCBI database using the MASCOT search engine v3.5 (Matrix Science Ltd., London). The following parameters were allowed: taxonomy restriction to Viridiplantae, one missed cleavage, 50 ppm mass tolerance in MS and 0.2 Da for MS/MS data, cysteine carbamidomethylation as a fixed modification and methionine oxidation as a variable modification. The confidence in the peptide mass fingerprinting matches (*P* < 0.05) was based on the MOWSE score and confirmed by the accurate overlapping of the matched peptides with the major peaks of the mass spectrum. Only significant hits, as defined by the MASCOT probability analysis (*P* < 0.05), were accepted. Database searches were performed using all available plant proteins, since the chrysanthemum genome has not been sequenced and many proteins are well conserved in plants. Identified proteins in this study were submitted to World-2D PAGE (Nos. XXXXX).

### Quantitative RT-PCR (qRT-PCR) for identified proteins

Plant material was pooled from six different cuttings bases for each sample for isolation of total RNA using the TaKaRa RNAiso reagent [46]. First-strand cDNA was synthesized from total RNA using the M-MLV reverse transcription system (TaKaRa, Japan) and oligo (dT)18 primer following treatment with RNase-free DNaseI (TaKaRa, Japan). cDNA was amplified by qRT-PCR using the primers listed in Table 2. To standardize the results, the relative abundance of *GAPDH* was used as the internal standard. Each reaction mix contained a 15 ng RNA equivalent of cDNA and 10 μM gene-specific primers. All assays were performed at least three times. The ΔCt (threshold cycle) values were calculated by subtracting the Ct value of GAPDH from the arithmetic mean Ct value of the target gene. The ΔΔCt values were calculated by subtracting the ΔCt values at 0-day-old cutting bases from the ΔCt value at each particular treatment time [47].

**Table 2:** List of the genes whose transcription profile was evaluated by qRT-PCR

**Gene name**	**Primer**	**5′-3′ primer sequence**	**Size (bp)**
CAB	F	TGATGGGTGCAGTTGAGGGTT	289
	R	GGGACGAAGTTTGTAGCGTAGGA	
ACO	F	ACTGATGGAACCCGAATG	248
	R	TTCTTCACTCAAGCCGTCGCAAT	
RPS5	F	CTCGGACGGGGATGAAGAAACAA	235
	R	CTGCTGGAGGTTGGGTGTATCAT	
PRS6B	F	ACTTCTTCGTCCTGGTAGGCTTGA	192
	R	ATCCCTGCTTCTTGACAGATGGC	
GME	F	GGTTCTTCTATGCGTCAAGTGCT	220
	R	GCCAAAAGGTCCATAGATGTTGTGAAA	
PGK	F	GGTCCTGAGGTTGAGAAACTGGT	197
	R	TTGGCAACACCTTCAGTAGACGC	
XTH	F	CAAGCACCCTTTTACGCATACTA	220
	R	CATTCTGGTGGGGTCACAGGGTT	
IDH	F	AGATTGCCAAAAGTTATCCCGACA	184
	R	ACATTTCCTCCTGGCATTACACC	
RbcL	F	AACGCCAGGGTTTTGATTACAGA	230
	R	ATACTCGGCTCCTGTCACGATGGC	
TIL	F	TGGACTGATGGTAAGAGGGGATA	130
	R	CACCAGTAACAGGAATGATAGGC	
ALDOA	F	AGAACCTTCGCCAACCATCAATC	250
	R	AGACCTGGTGGAGTAACAAGTAG	
GDH	F	TTCGGAAATGTTGGTTCGTGGGC	130
	R	ATGTTTTAGCAGGTTGGGGATGT	
ALD	F	GACTACATTGACAAAGTTGGGGAGC	260
	R	AAGCCGACCTGTCTCATTCCACCC	
PCNT115	F	AAGAATCAAGTTGGGTTCACAGG	210
	R	CCCTTCAAAGCCTTTCCAAGTAA	
AtpB	F	TATGGTTAGGACTATTGCTATGGATGG	290
	R	TCTTTCCTCCTCTTTGATACGGT	
ADF2	F	GCATCTGGAATGGCTGTTGACGA	201
	R	GTCAAAATCAAAGACAGCATAGCG	
CA1	F	GTATTTGCCTGCTCGGATTCTCG	280
	R	CAGGCAACCCGATTTTGACCCAT	
CHLM	F	TGGCTTGGGACTTGGGGTTCTTG	280
	R	CACAGATAAGCAGAAGAAACGAG	
MDH	F	CAAGATGGTGGAACAGAGGTGGT	174
	R	CTTTGAGGCGAAGAATGGTAGTT	
S-ACP-DES	F	GATGGGCGTGATGACAACCTCTT	180
	R	TGGCAACCCACAAACATACTCTT	
GAPDH	F	CTGCTTCTTTCAACATCATTCC	170
	R	CTGCTCATAGGTAGCCTTCTTC	

### Western-immunoblot analysis

A total of 18 μg protein was loaded per lane, separated by SDS-PAGE, transferred to a PVDF membrane (Bio-Rad, Hercules, CA), and probed with the CmACO monoclonal antibody. The membrane was incubated for 1 h with TBST + BSA [Tris-buffered saline-Tween 20; 10 mM Tris, 250 mM NaCl, 0.1% (v/v) Tween 20 + 1% (w/v) BSA, pH 7.2] and then treated with a 1:1000 dilution of the monoclonal anti-chrysanthemum ACO for 3 h. The membrane was rinsed with TBST + BSA (4 × 15 min), and then incubated with mouse anti-goat alkaline phosphatasetagged secondary antibody (Sigma-Aldrich) diluted 1:3000 in TBST + BSA for 1 h. After washing, the membrane was allowed to equilibrate in reaction buffer (100 mMTris, 100 mMNaCl, 10 mMMgCl2, pH9.5), and protein immune detection was visualized by a reaction with p-Nitro-Blue tetrazolium chloride (NBT) and X-Phosphate in the reaction buffer (45 mL of NBT and 35 mL of X-Phosphate/10 mL of buffer).

### Measurement of ethylene evolution

Six different cuttings bases for each sample were harvested and transferred to a 10 mL vial, and capped gas tight. Ethylene was measured after 6 h in the head space using a gas chromatograph (Aglient 6890 N GC System). Cuttings bases were weighed, and ethylene production was expressed as nanoliter per mg fresh weight per hour.

### Ethylene inhibitor AgNO_3_ pretreatment and rooting phynotype

Excised leafy cuttings of chrysanthemum ‘Jinba’ with two to three fully expanded leaves were pretreated with 60 μM AgNO_3_ for 30 minutes [48], then transferred to perlite and vermiculite (1:1) substrate without any external additives. Cuttings were kept in a greenhouse (day/night temperature 25/18°C, photoperiod 16 h, light intensity 50 μmol m^−2^ s^−1^, relative humidity 70%). Photographs of AgNO_3_ cuttings and control plants (without AgNO_3_) were taken every day.

## Competing interests

The authors declare that they have no competing interests.

## Authors’ contributions

FC designed and supervised the work. SC and JJ participated in the design of the study and help in the drafting of the manuscript. RL carried out protein extraction, 2-DE gel analysis, image and statistical analysis and LC-ESI-MS/MS and drafted the manuscript. ZL conducted western blotting and cutting, CZ preformed the ethylene evolution detection and cutting. SH, HL and HW carried out the cultivation of plants, anatomical investigation and ethylene evolution measurement. JG carried out Western blotting. JS and AP carried out gene transcriptional analysis. All authors read and approved the final manuscript.

## Supplementary Material

Additional file 1 **1.** PMF maps of the 69 differentially accumulated proteins in the base of chrysanthemum cuttings during adventitious root primordium formation. **2.** The nucleotide sequences of genes in this study. Click here for file
