# Supplementary material for: Proteomic changes in the base of chrysanthemum cuttings during adventitious root formation
Source: BMC Genomics. 2013 Dec 26;14:919. doi: 10.1186/1471-2164-14-919 (PMC3937169; doi:10.1186/1471-2164-14-919)
Supplement: Additional file 1 — 1. PMF maps of the 69 differentially accumulated proteins in the base of chrysanthemum cuttings during adventitious root primordium formation. 2. The nucleotide sequences of genes in this study. [file 1471-2164-14-919-S1.doc]

1.PMF maps of the 69 differentially accumulated proteins in the base of chrysanthemum cuttings during adventitious root primordium formation.

Spot 1


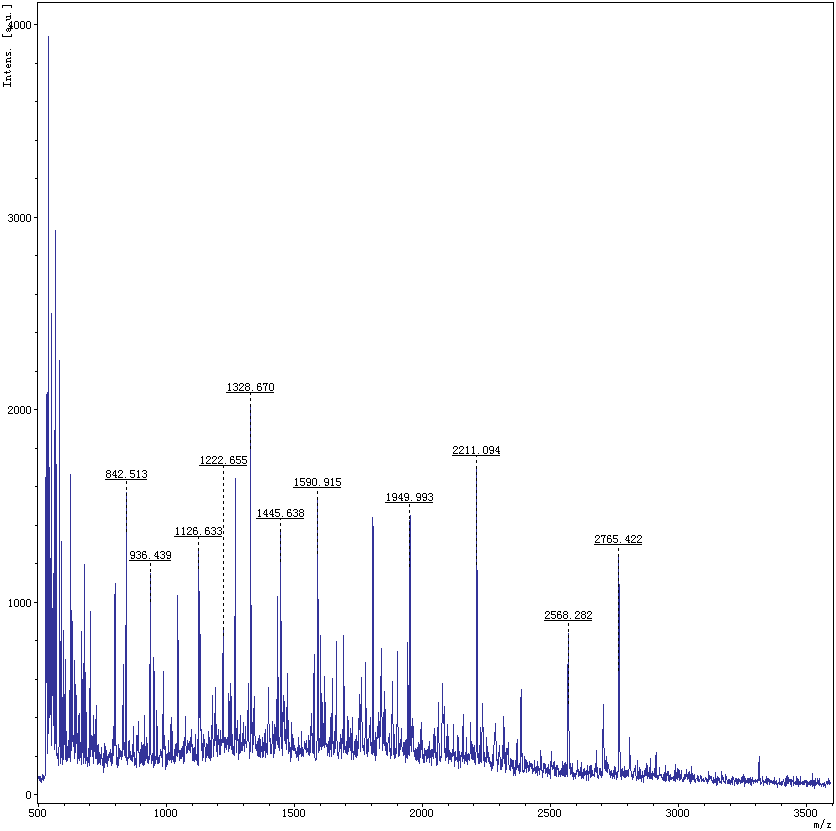


Spot 2


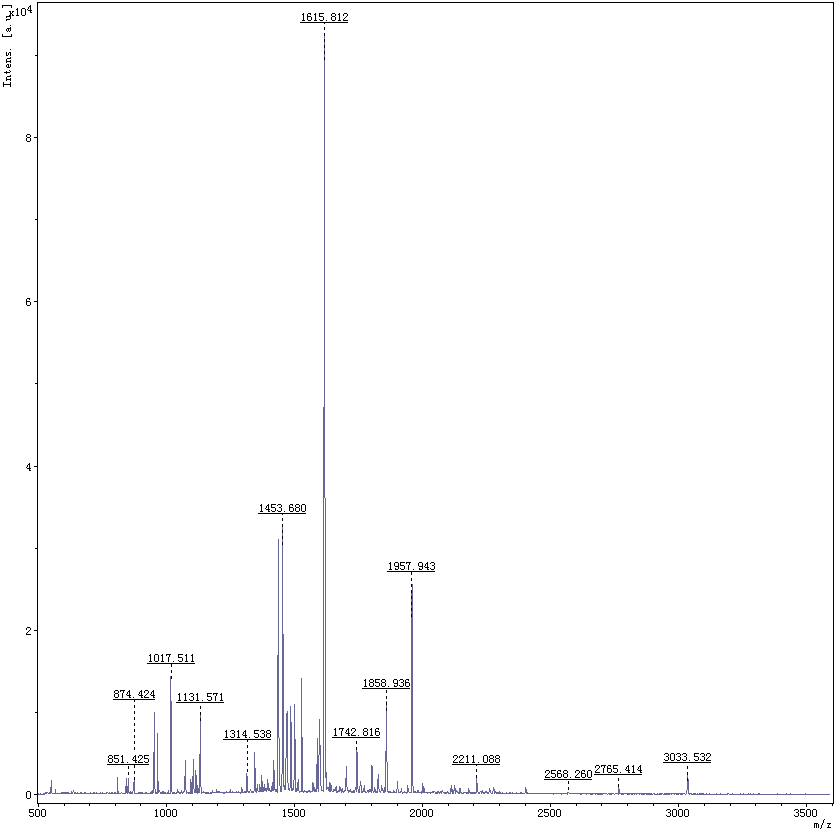


Spot 3


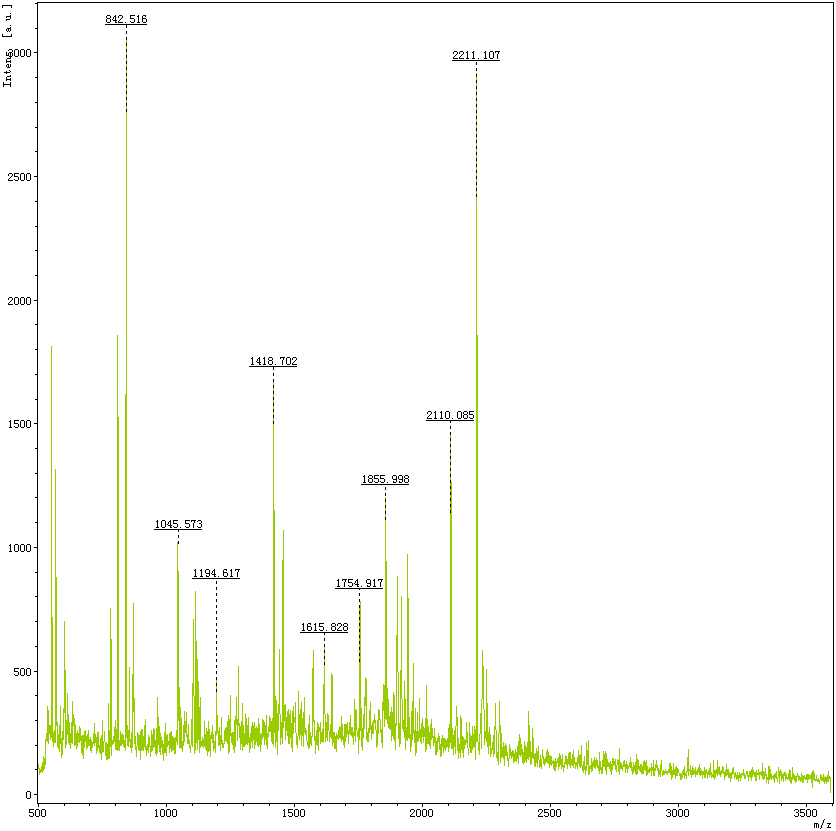


Spot 4


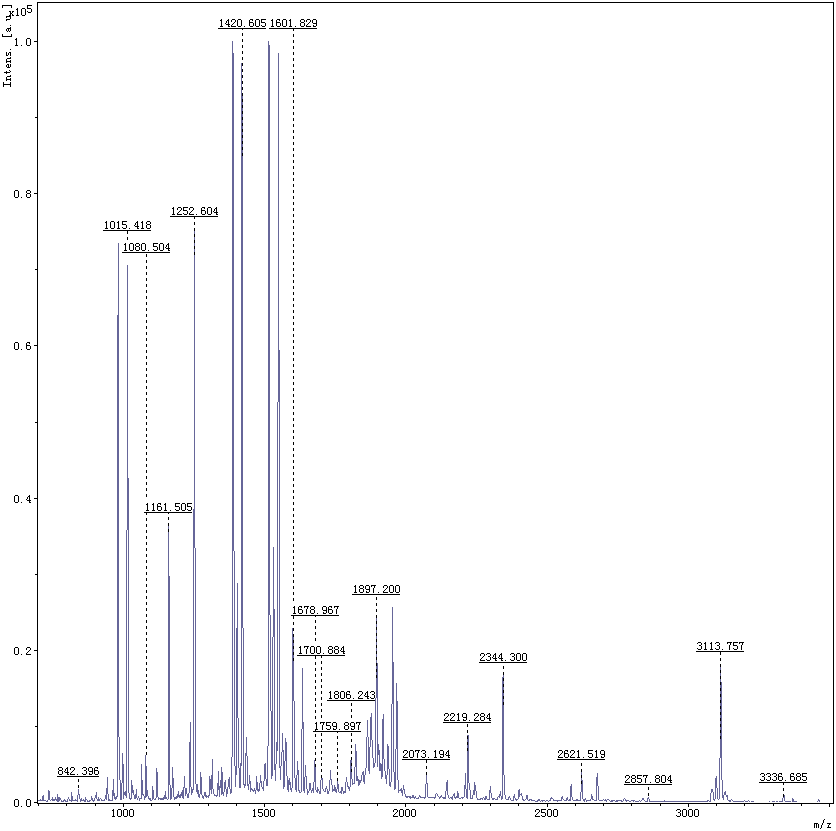


Spot 5


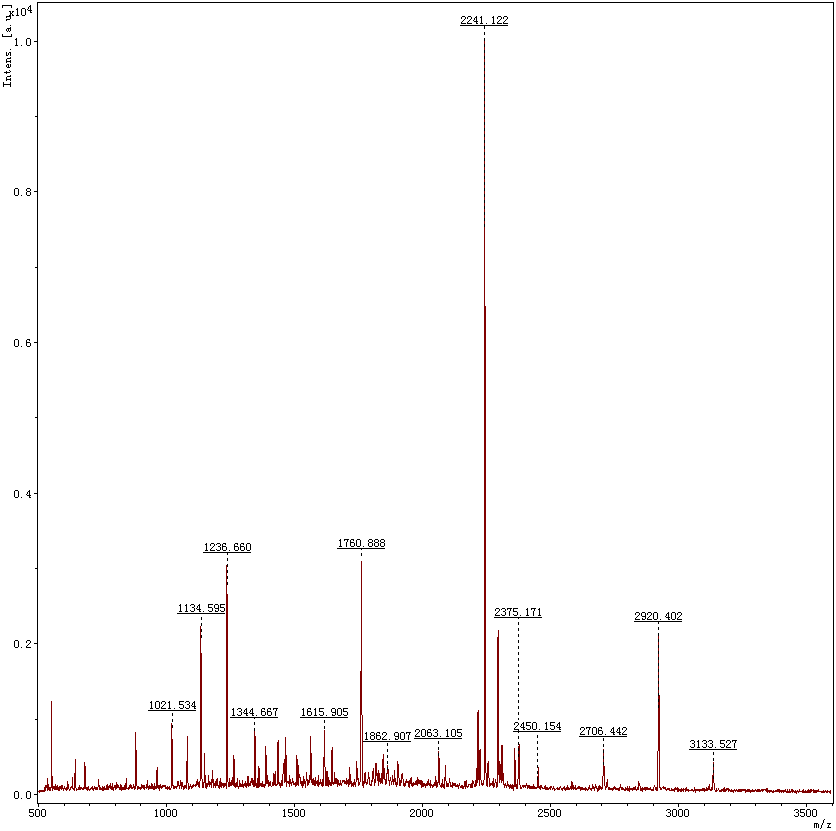


Spot 6


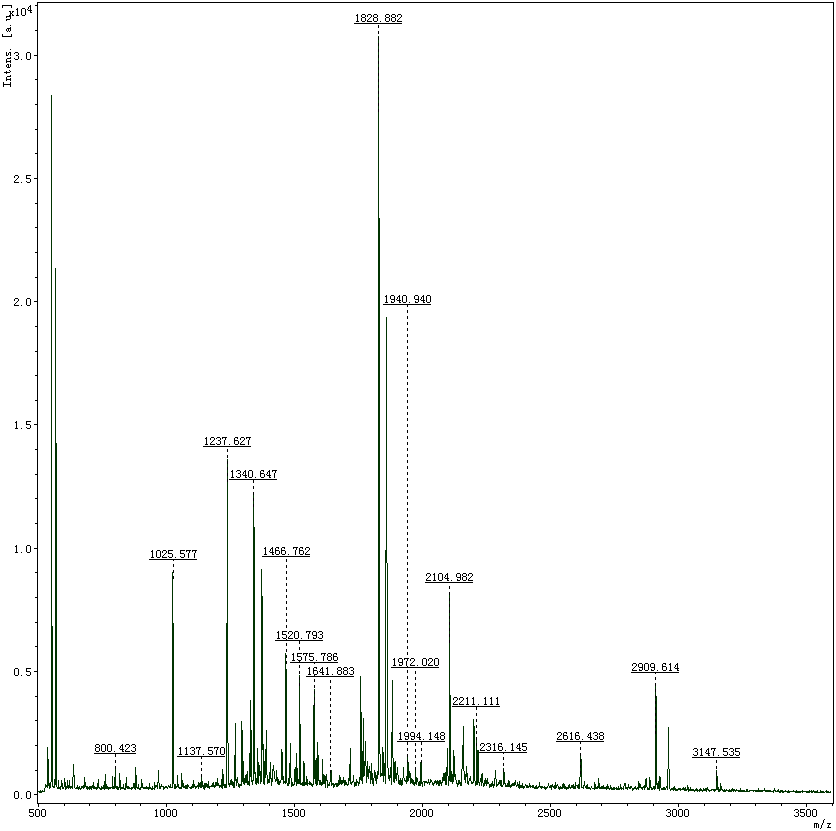


Spot 7


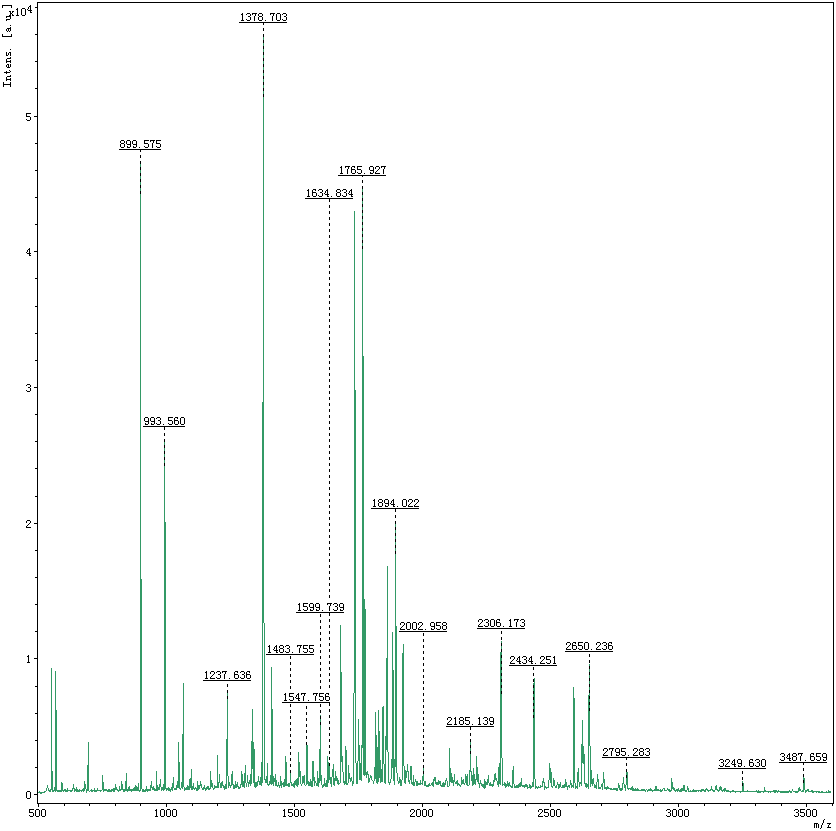


Spot 8


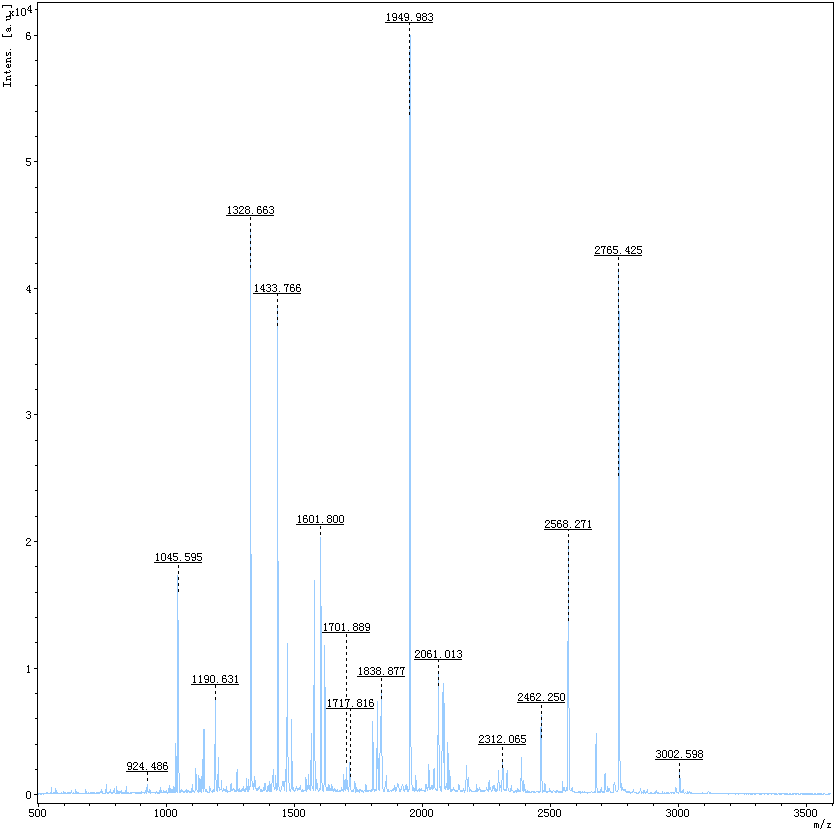


Spot 9


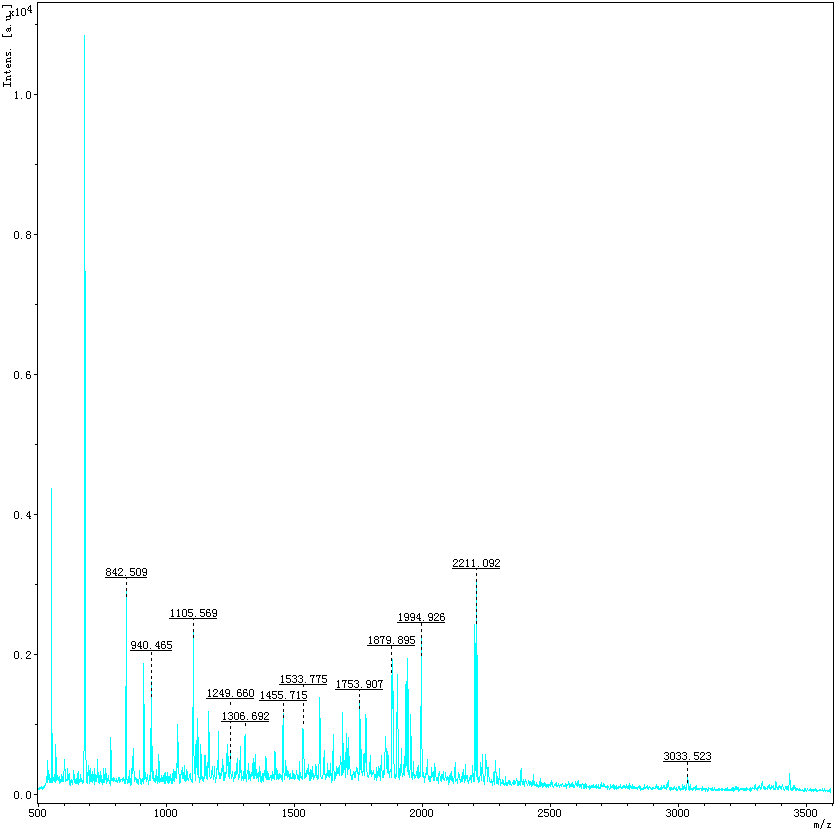


Spot 10


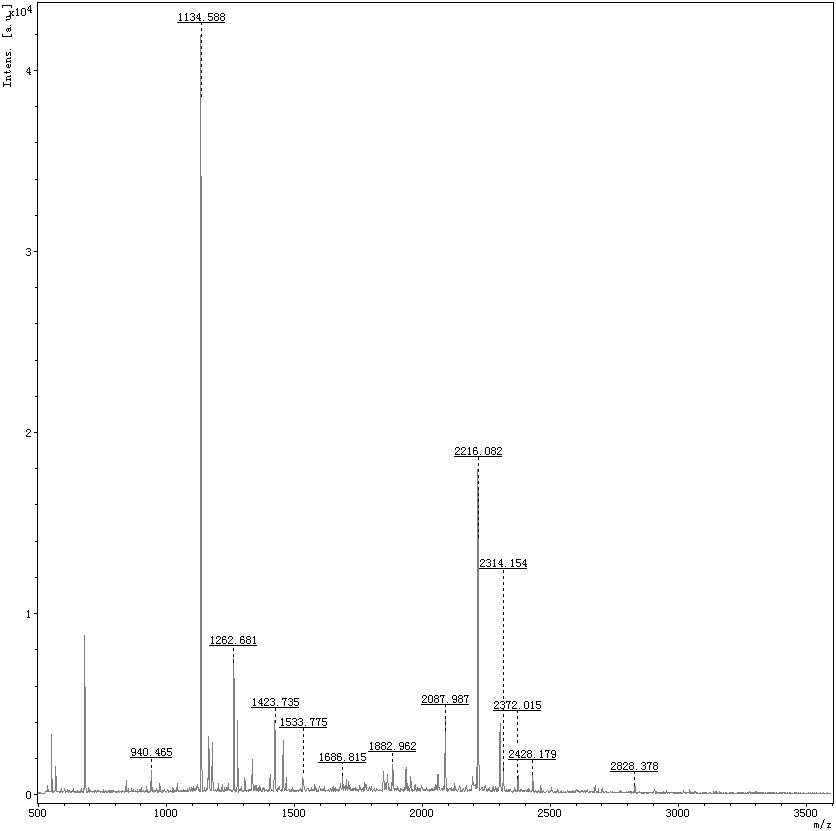


Spot 11


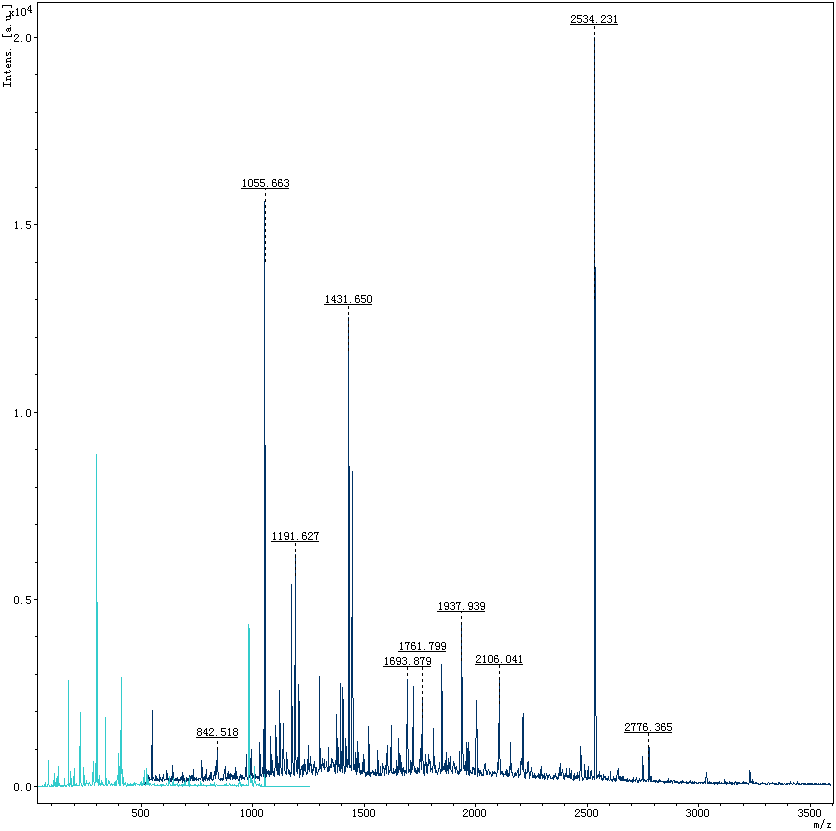


Spot 12


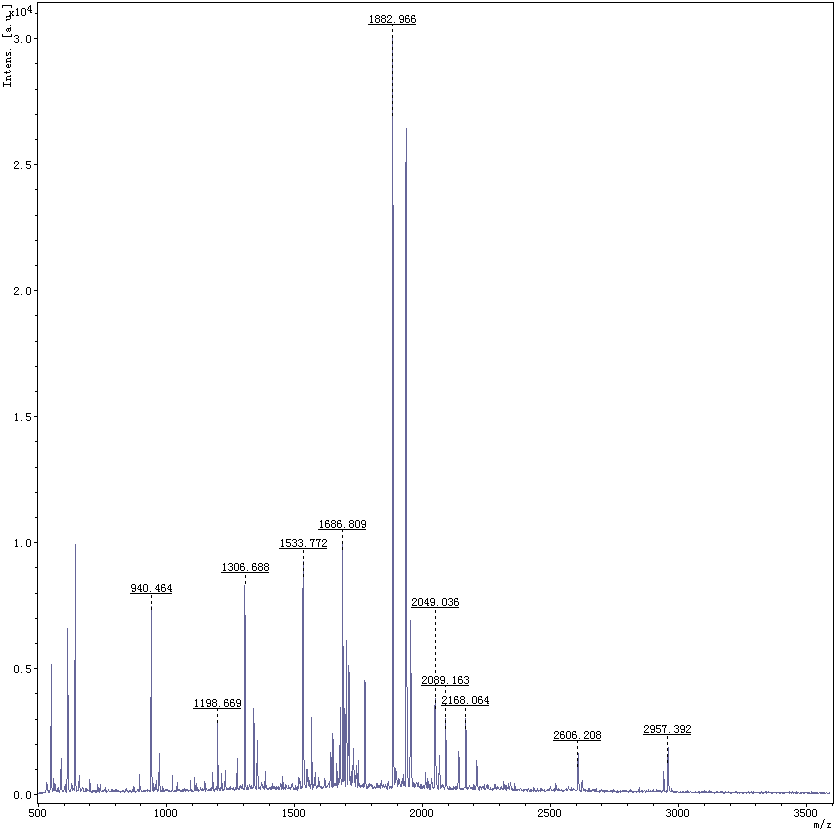


Spot 13


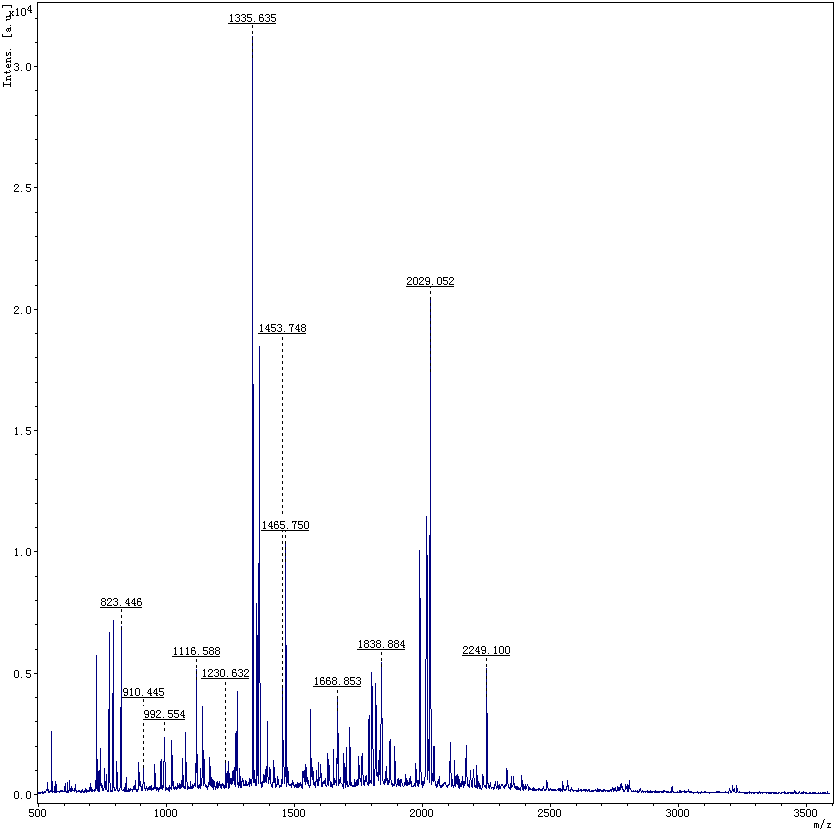


Spot 14


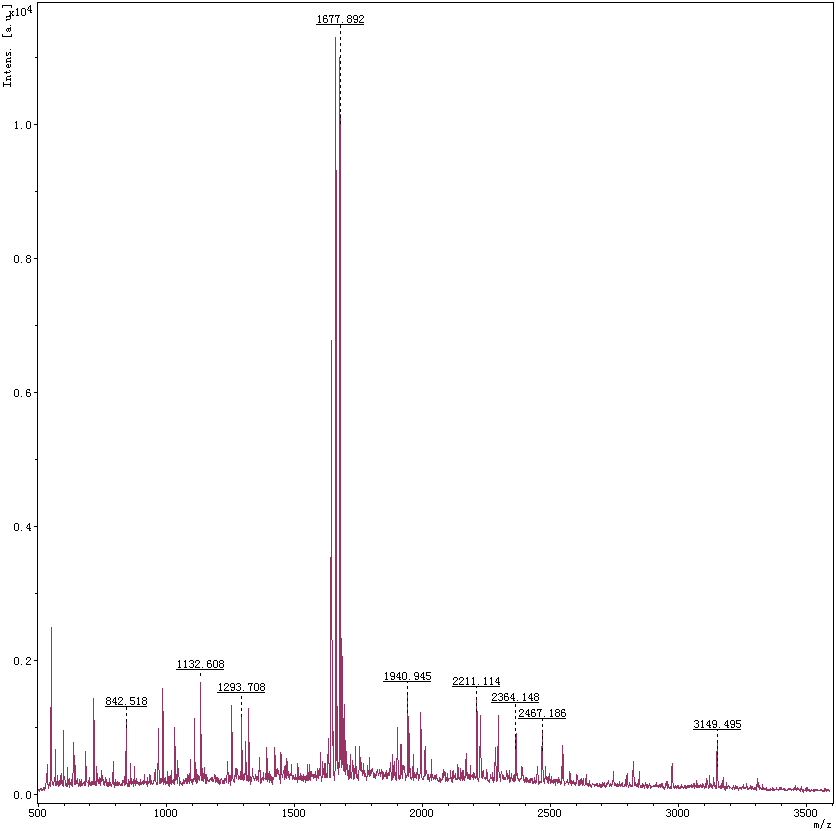


Spot 15


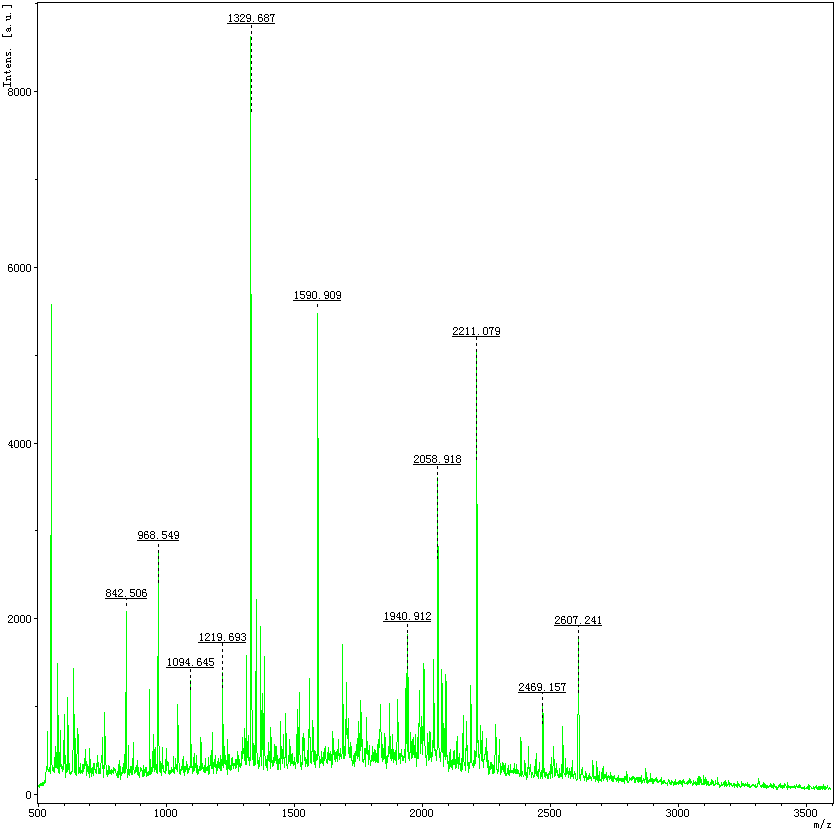


Spot 16


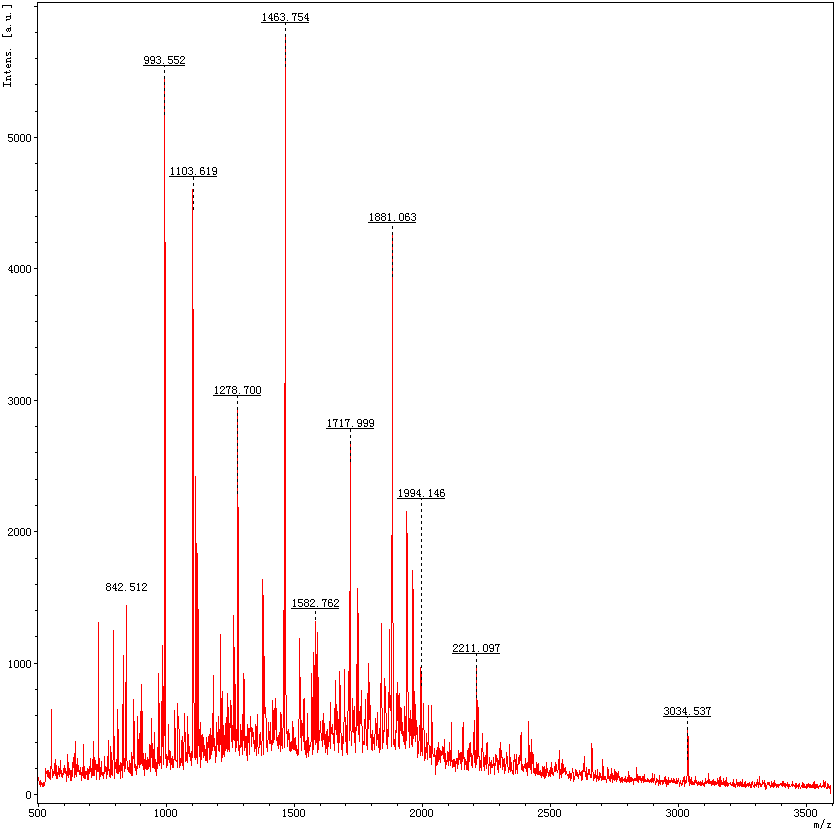


Spot 17


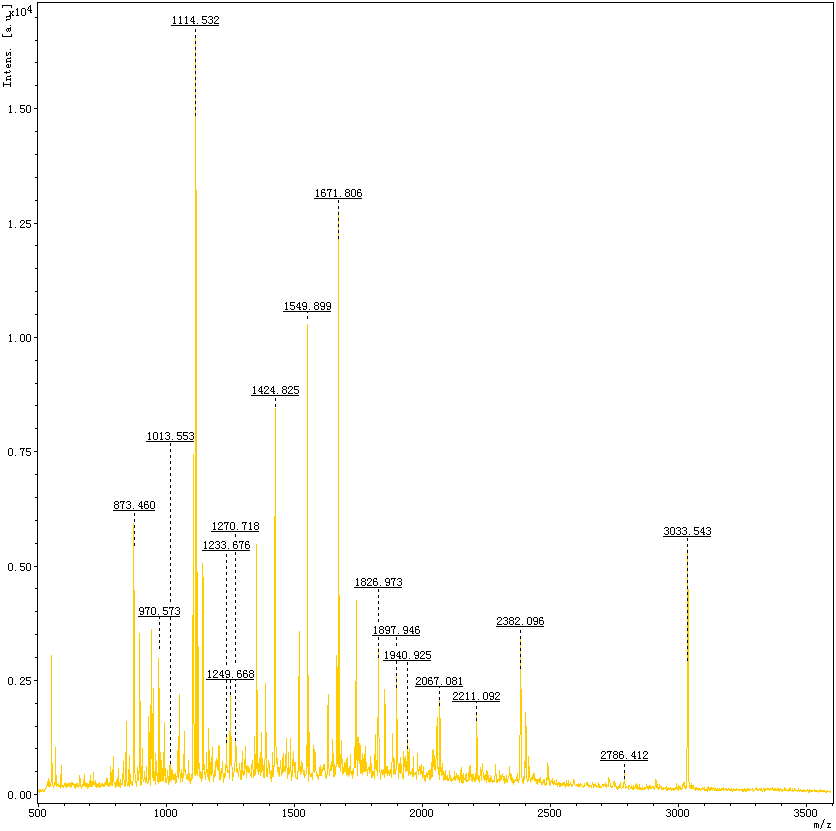


Spot 18


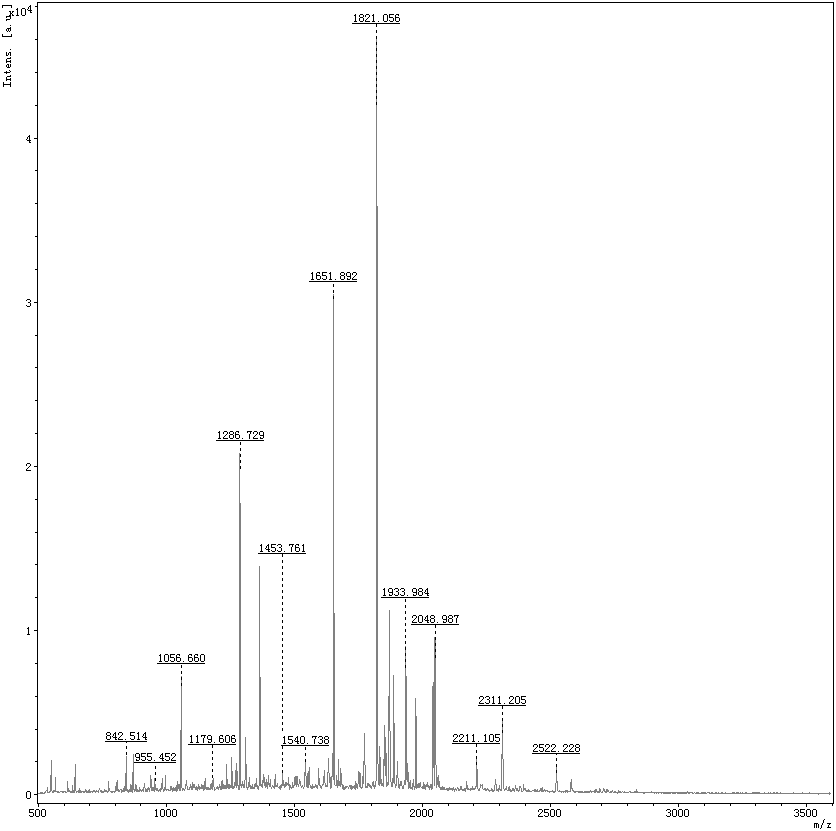


Spot 19


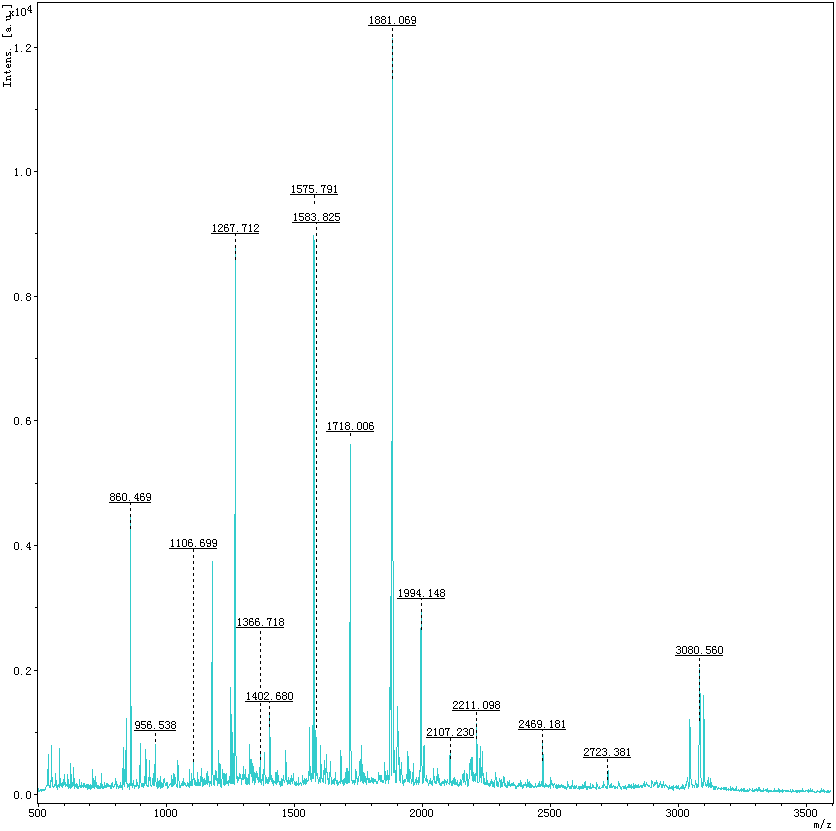


Spot 20


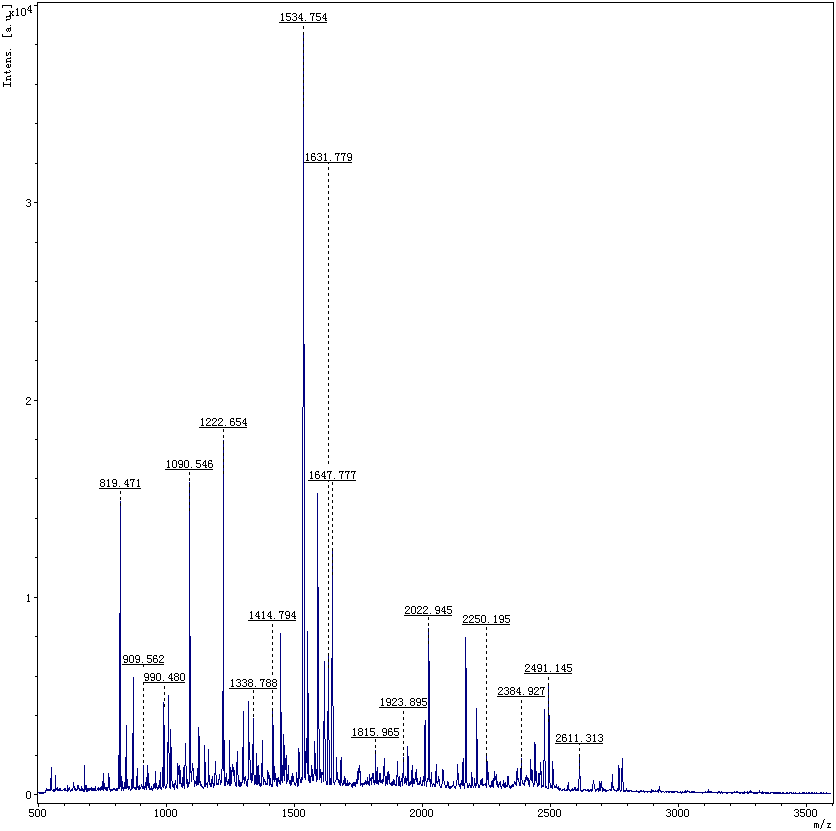


Spot 21


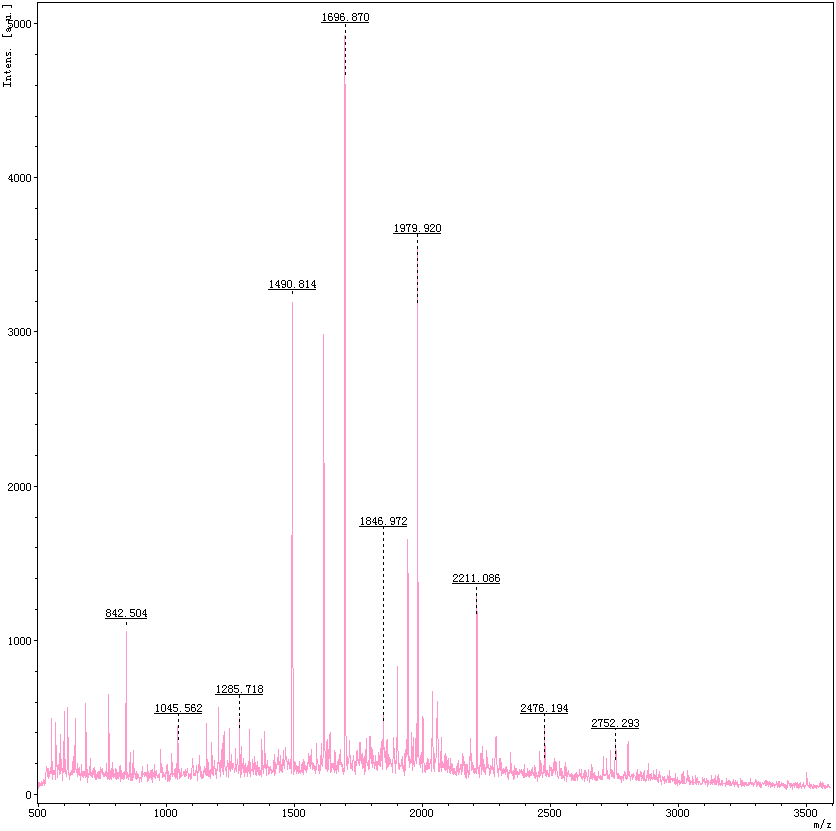


Spot 22


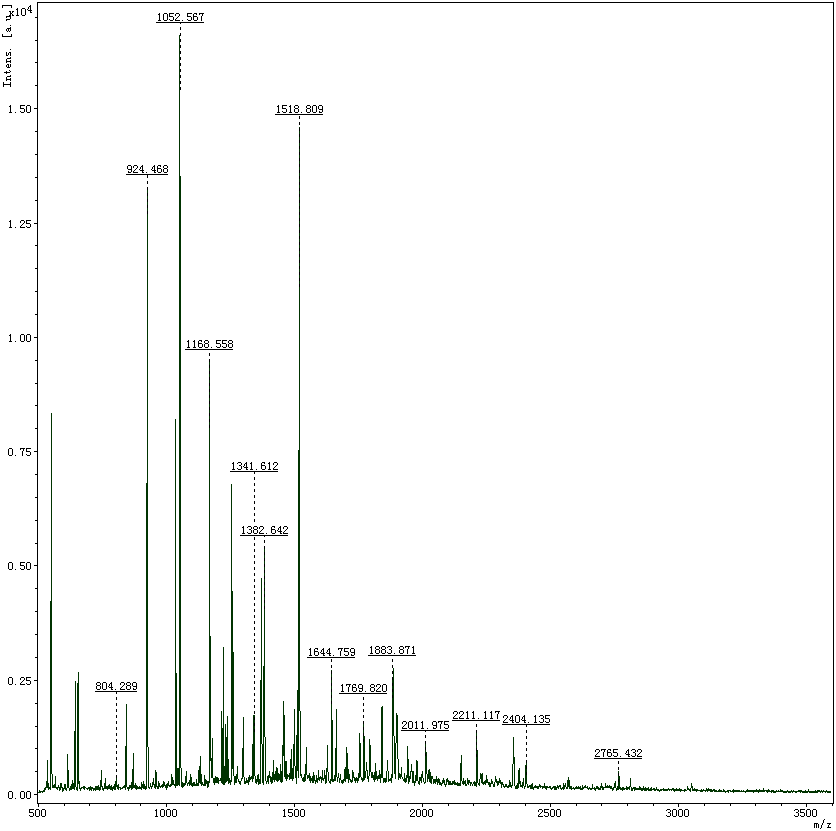


Spot 23


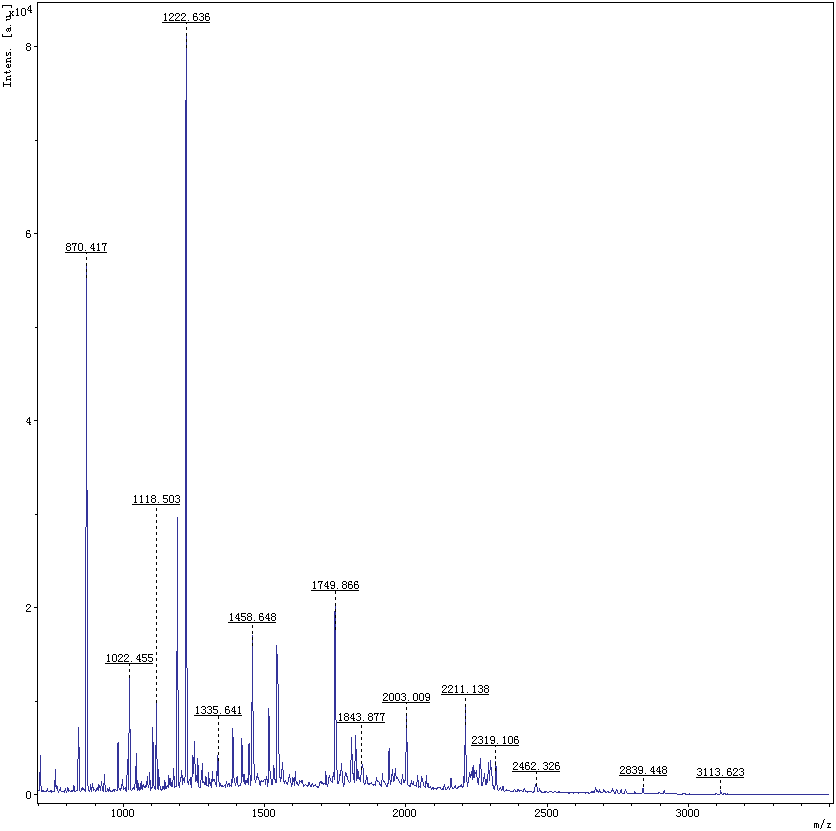


Spot 24


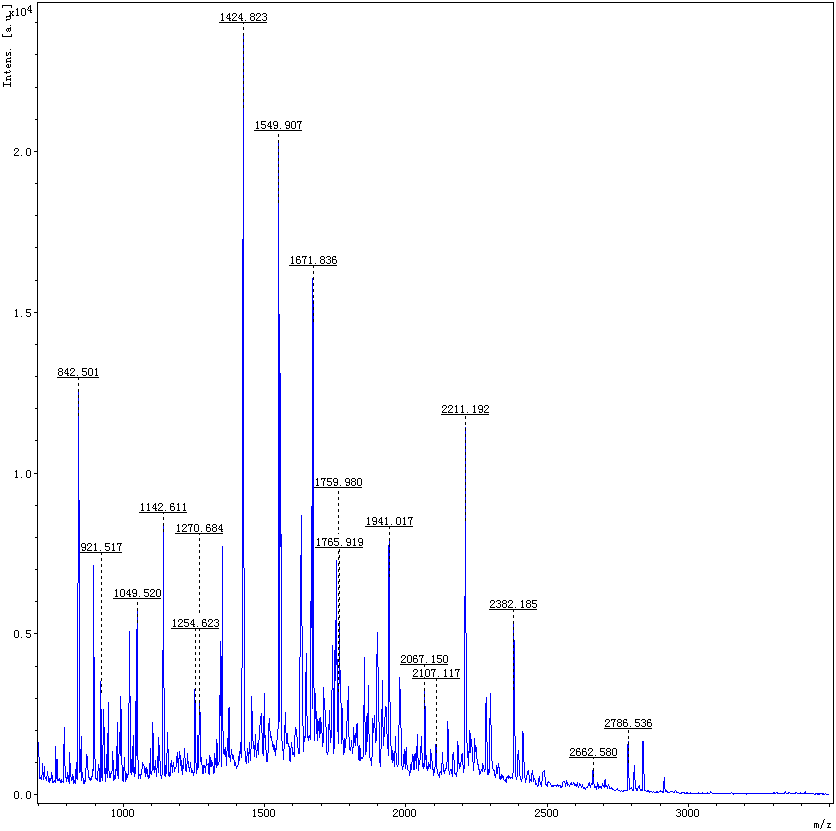


Spot 25


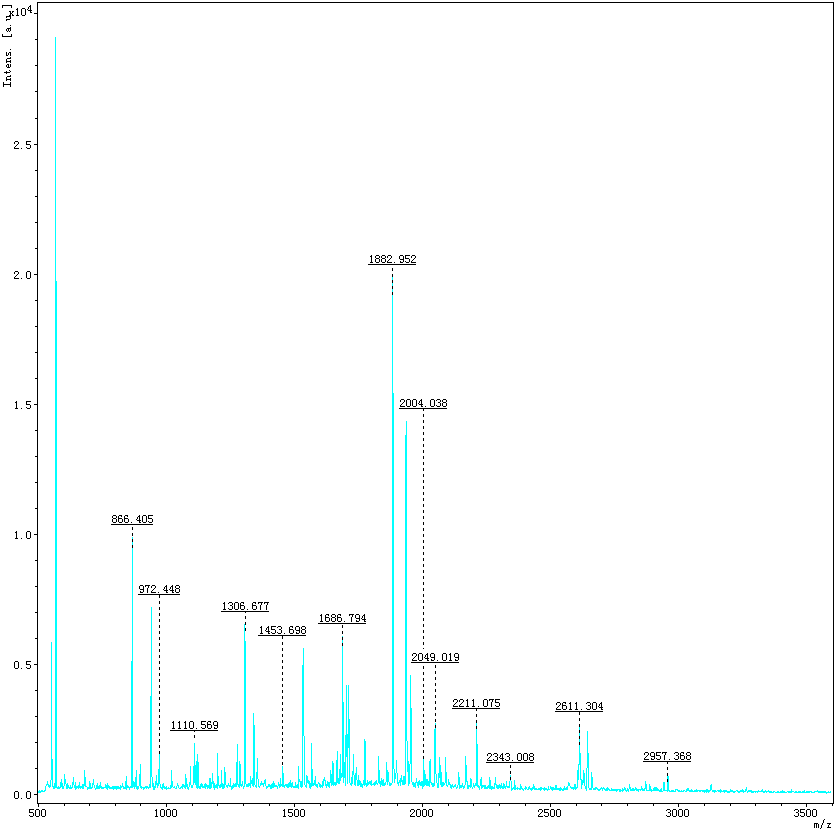


Spot 26


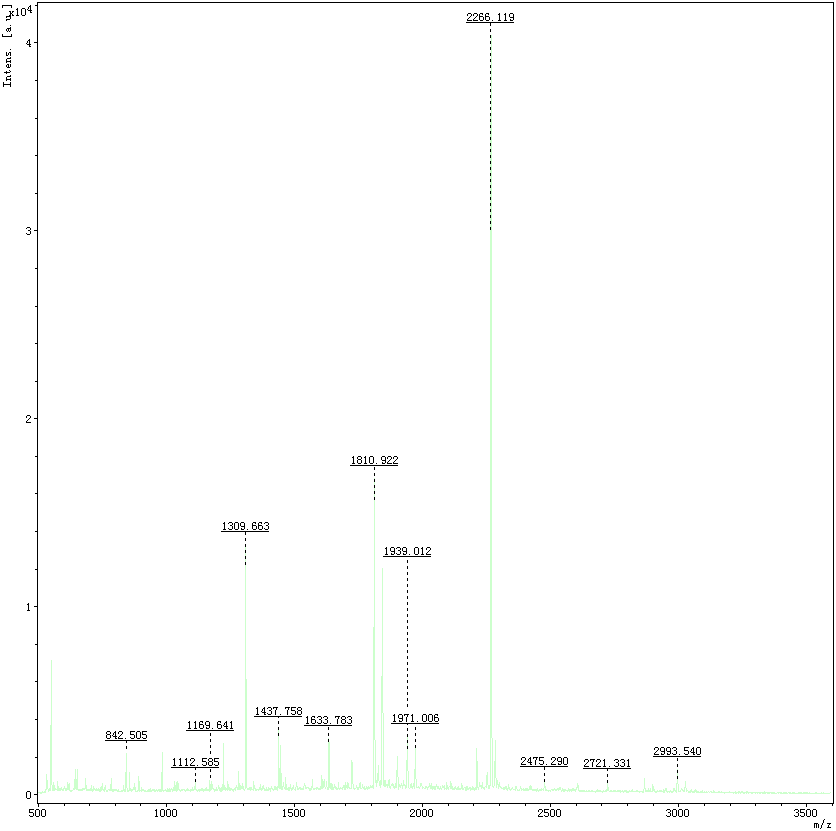


Spot 27


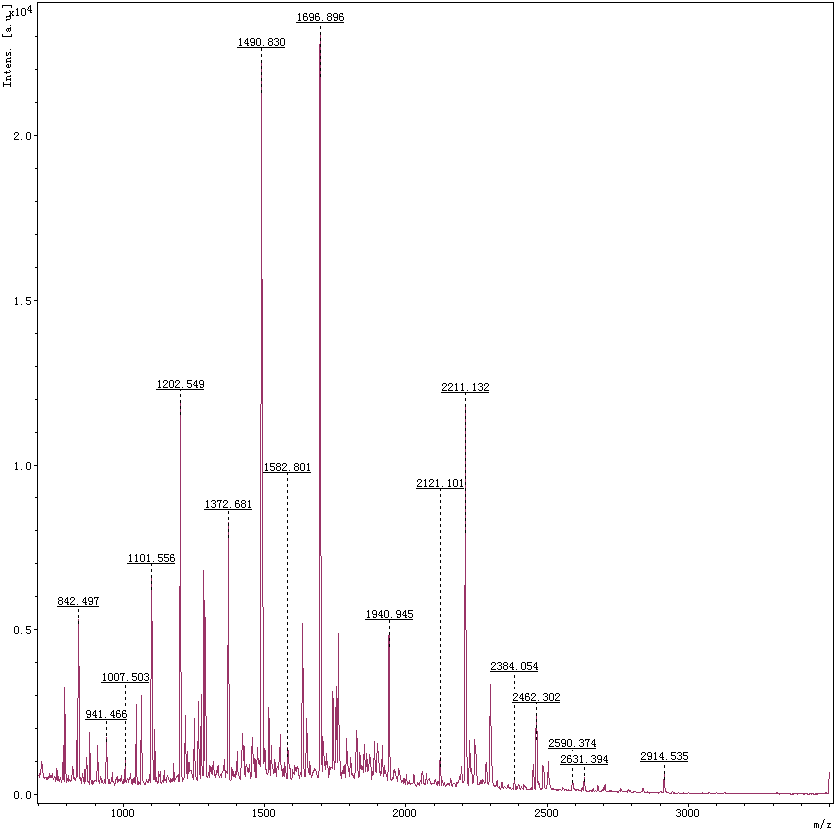


Spot 28


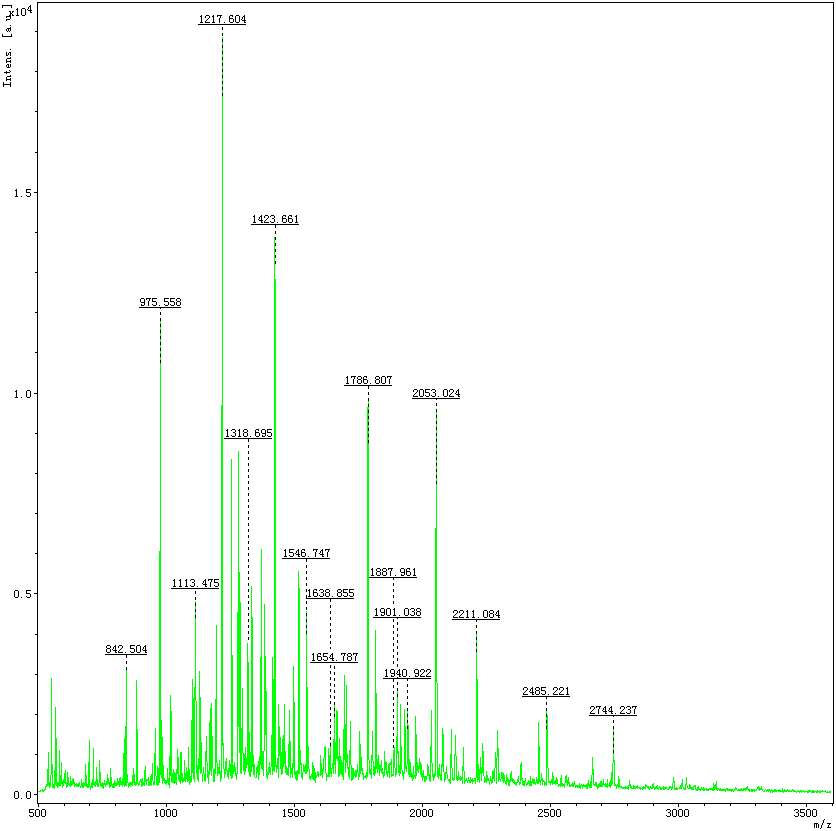


Spot 29


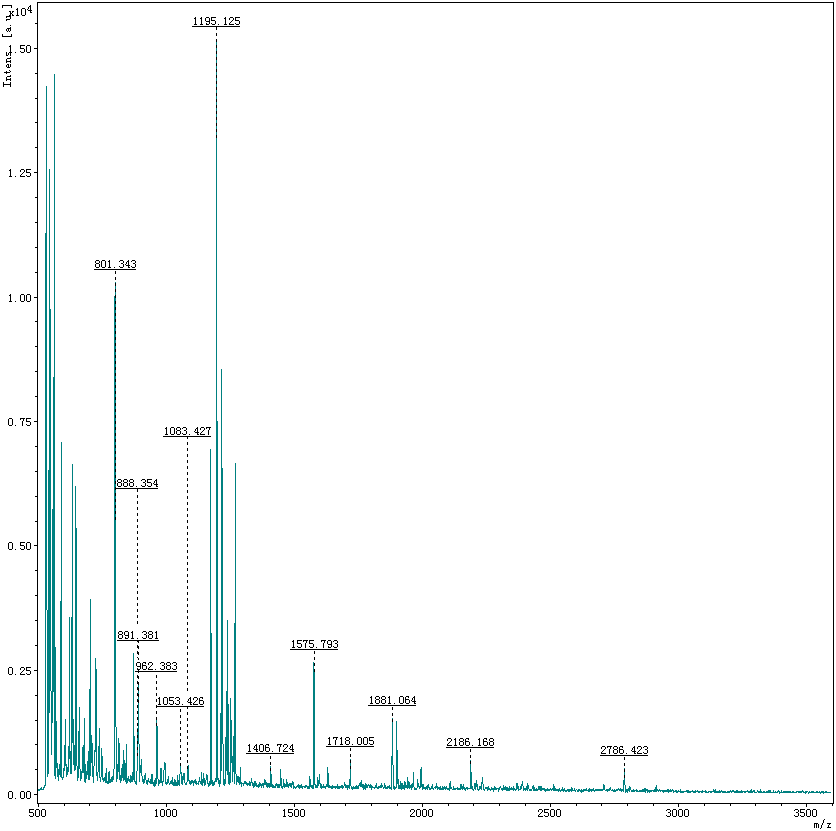


Spot 30


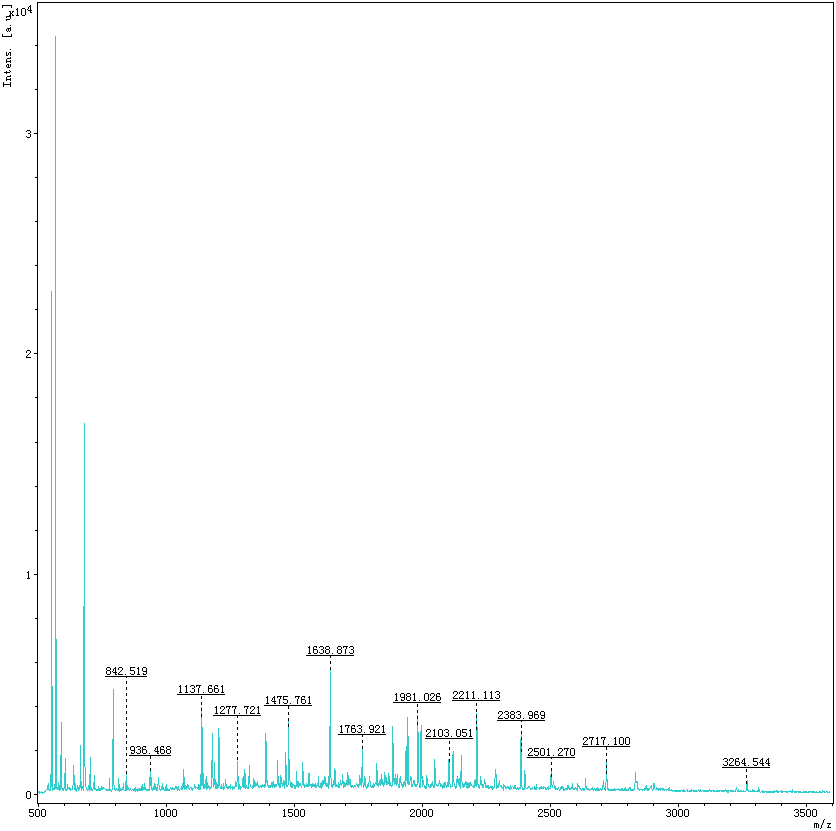


Spot 31


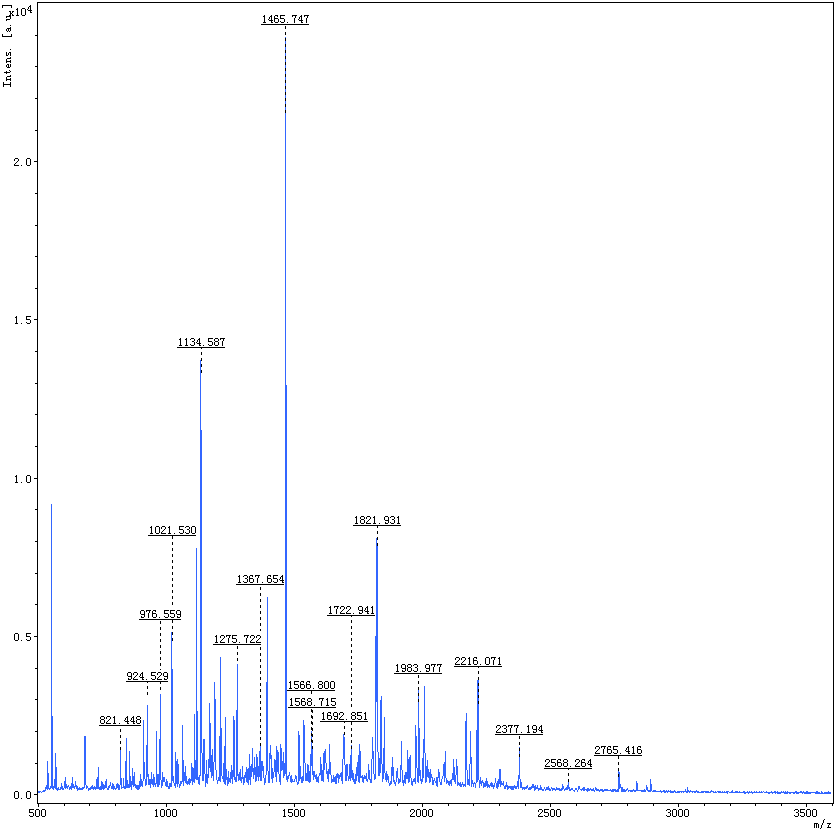


Spot 32


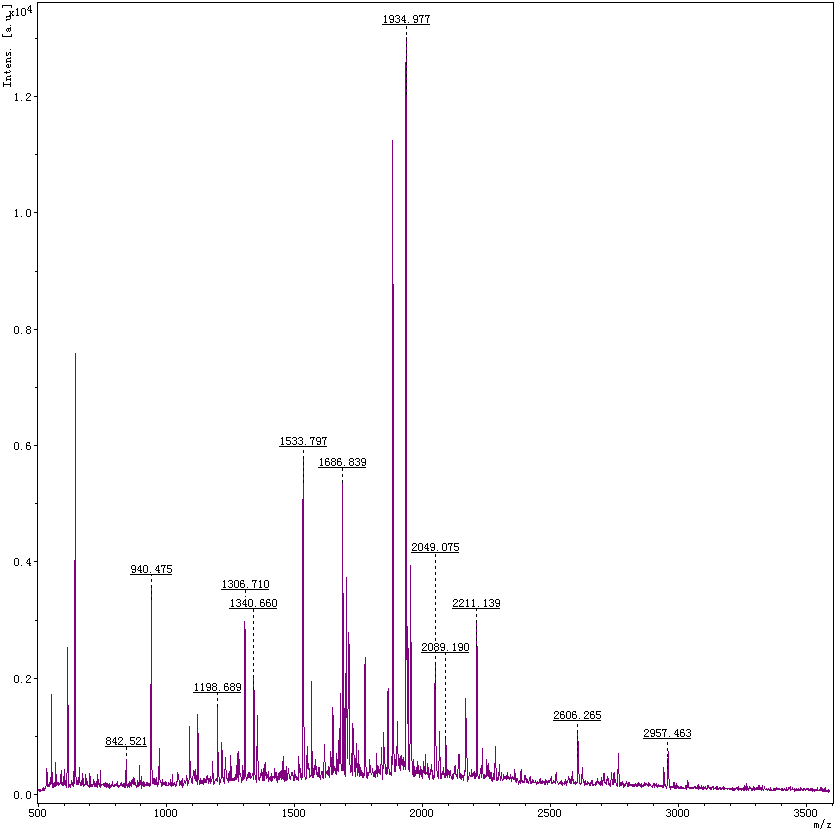


Spot 33


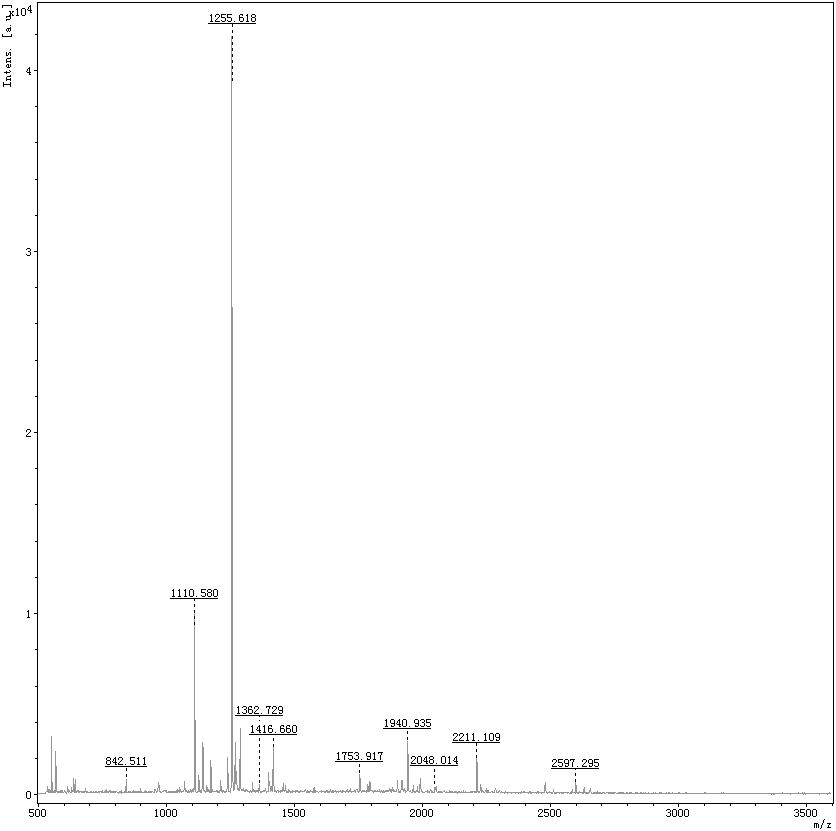


Spot 34


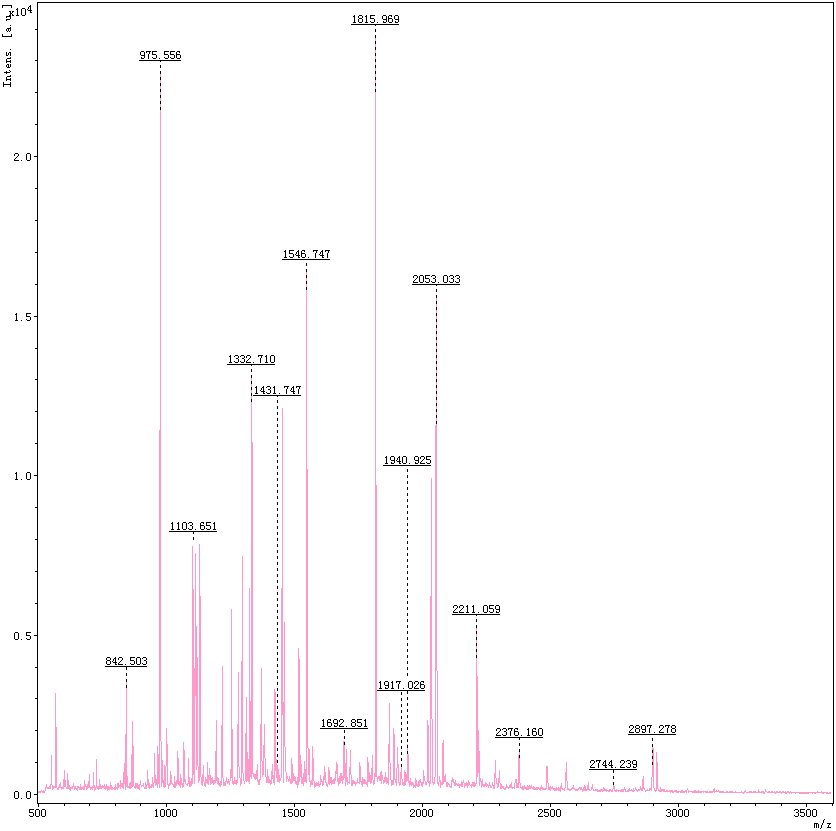


Spot 35


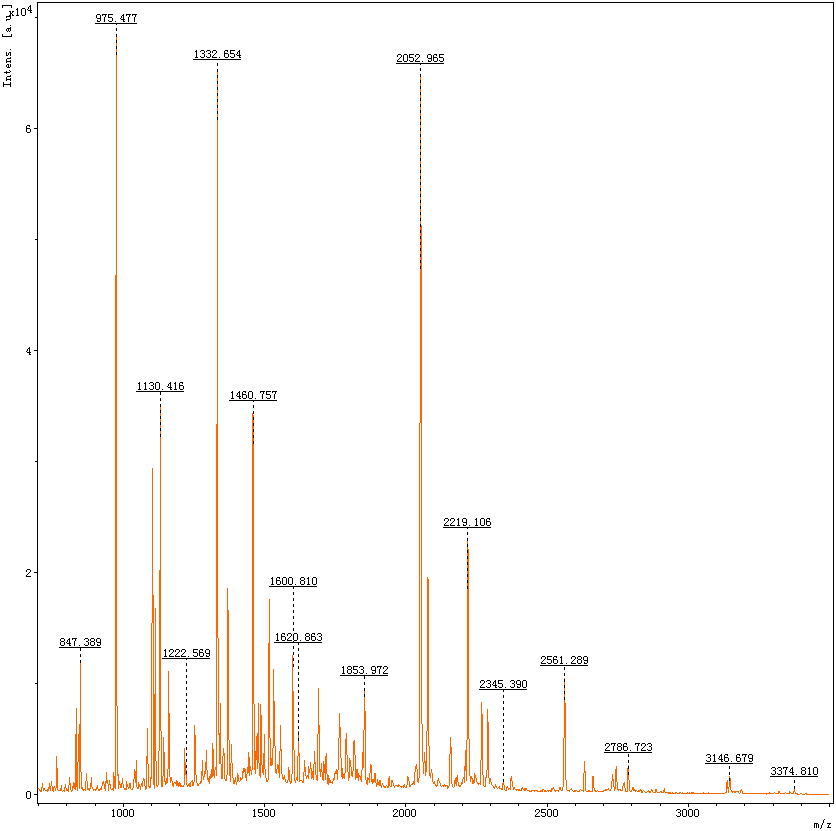


Spot 36


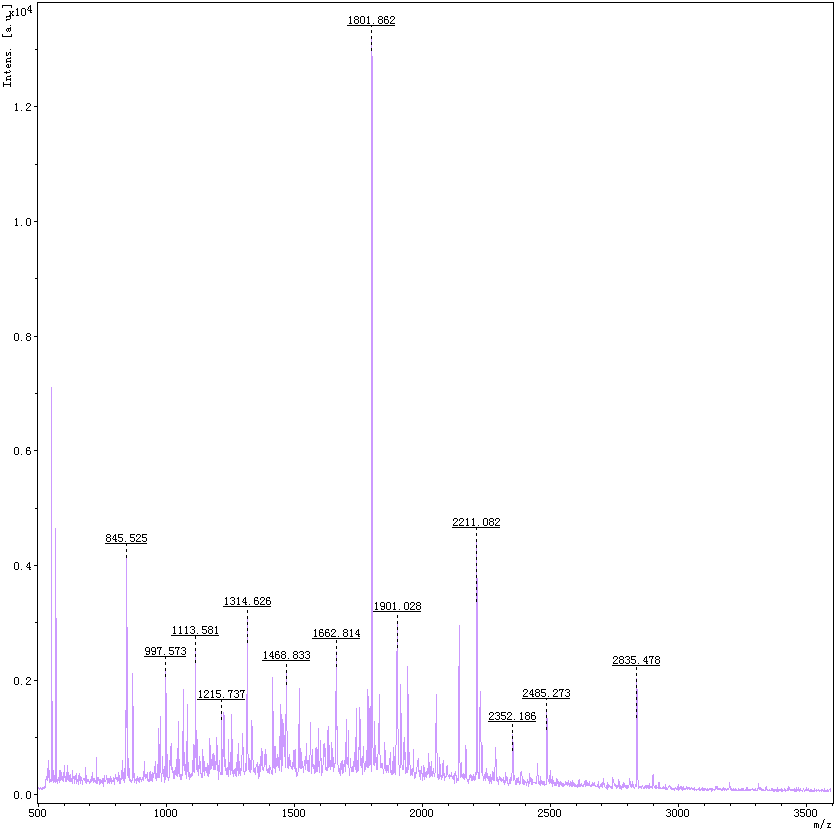


Spot 37


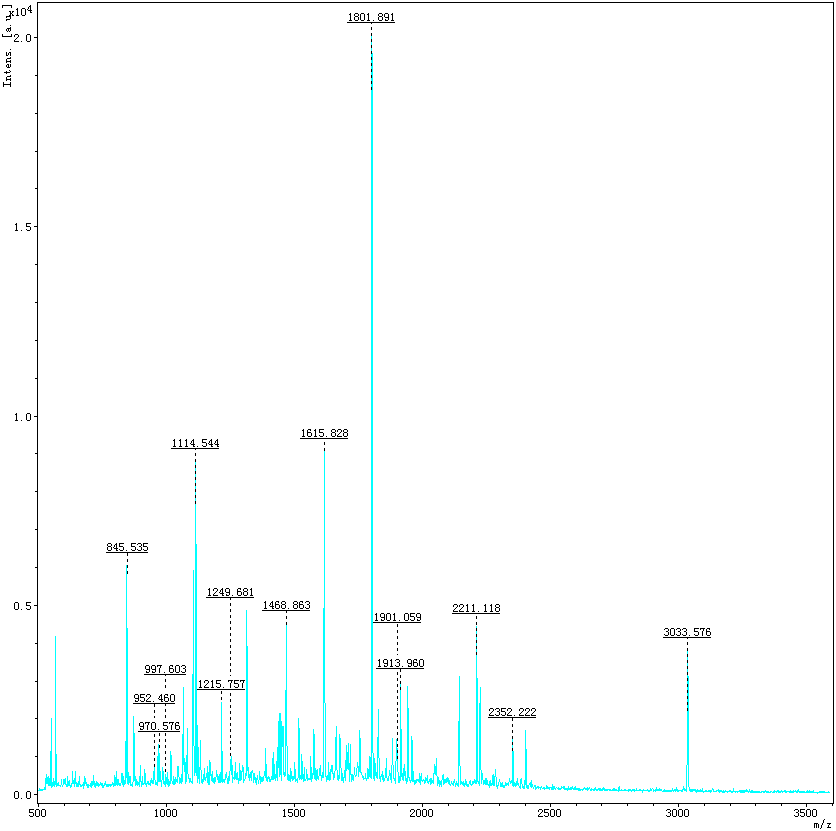


Spot 38


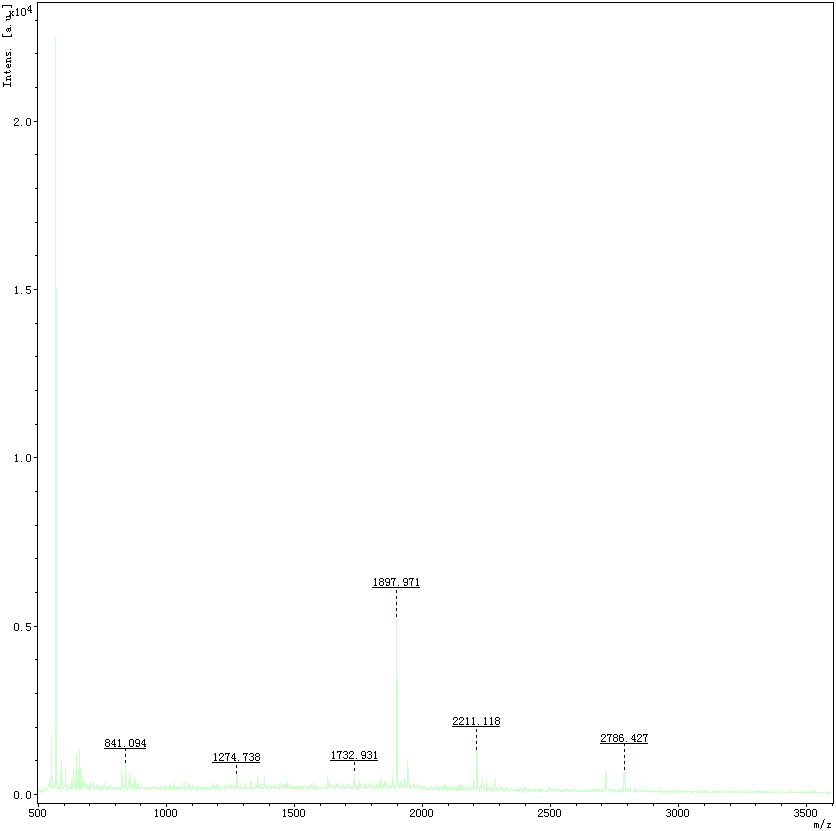


Spot 39


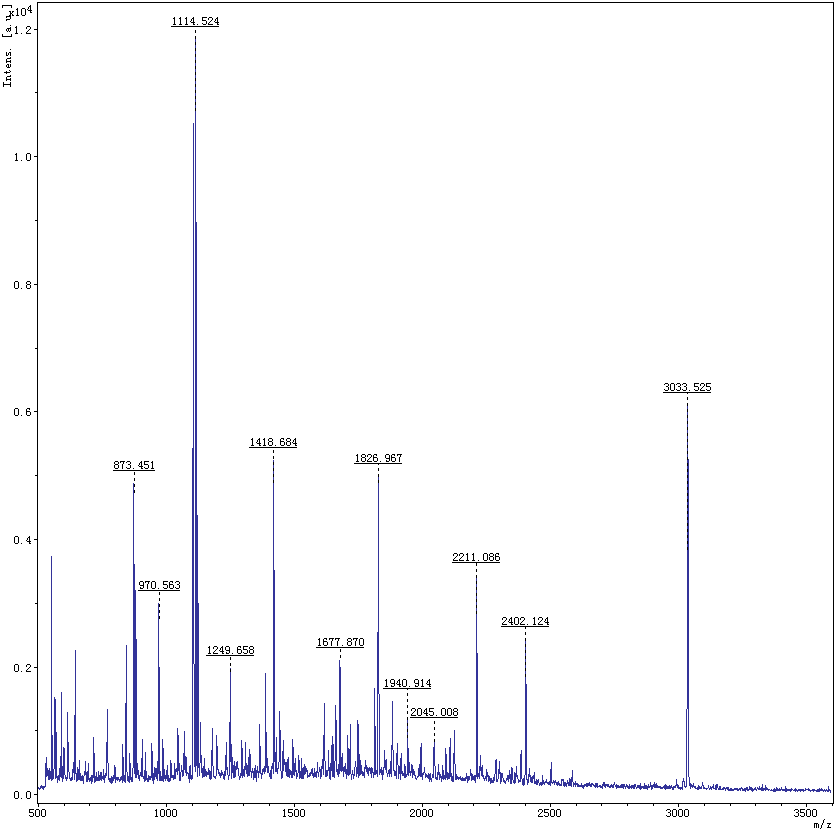


Spot 40


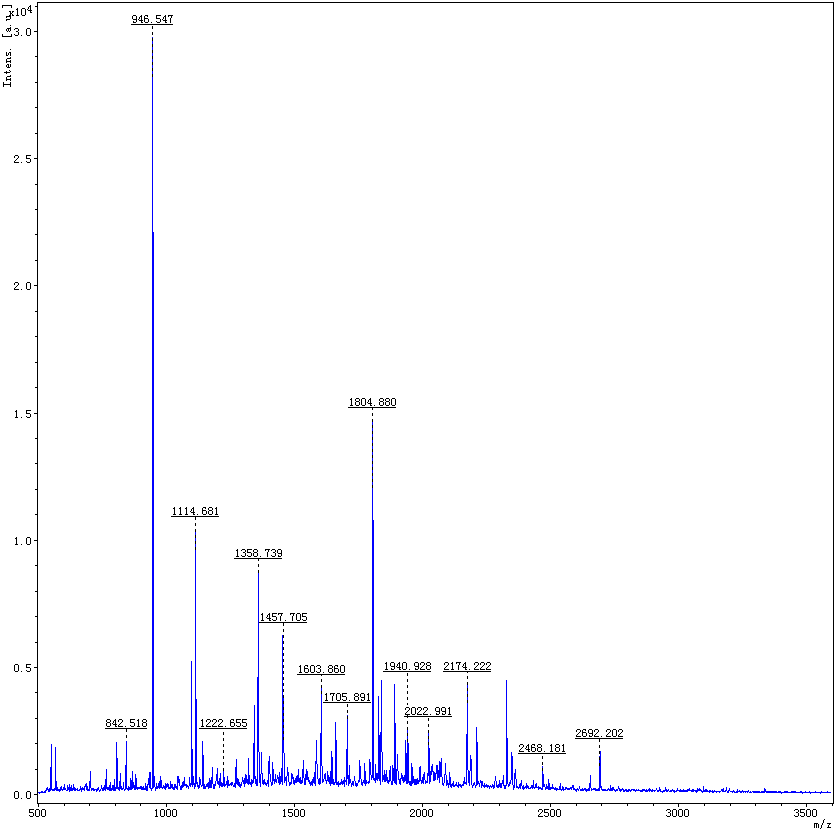


Spot 41


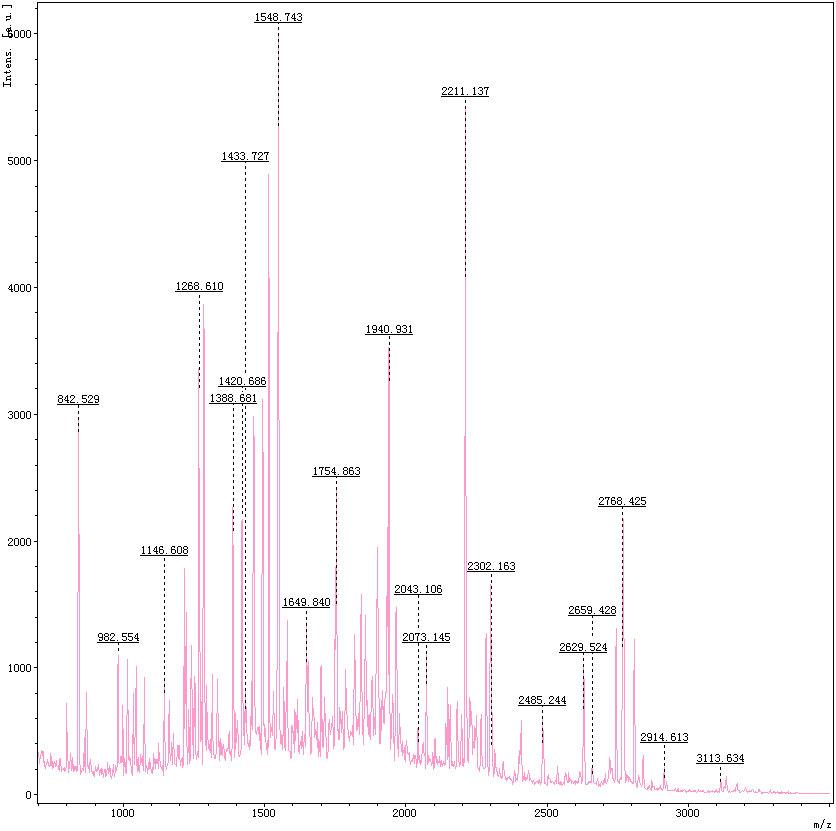


Spot 42


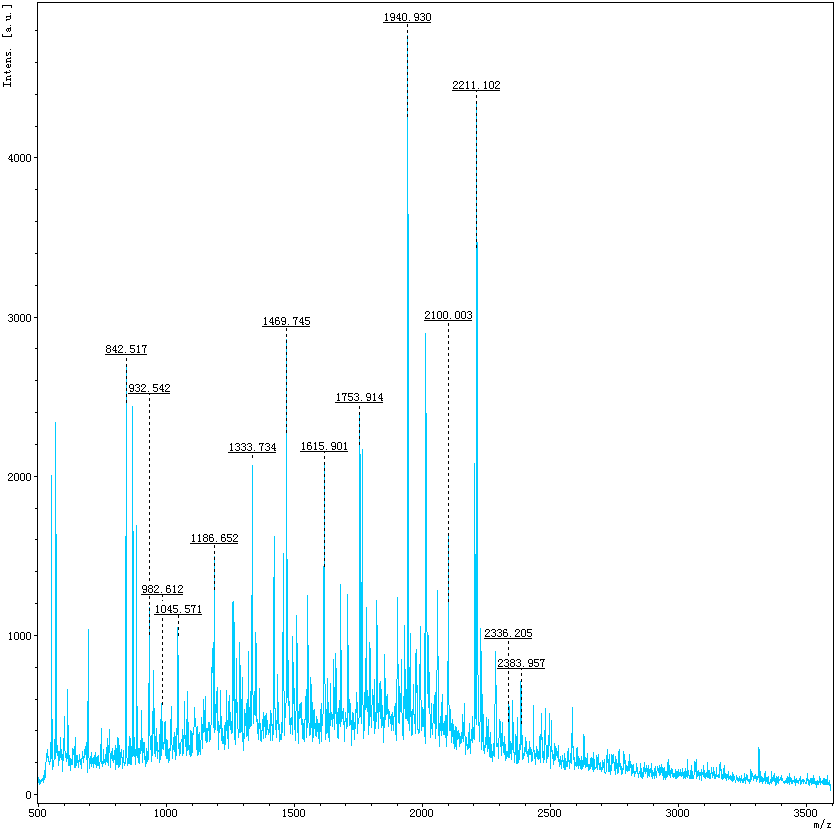


Spot 43


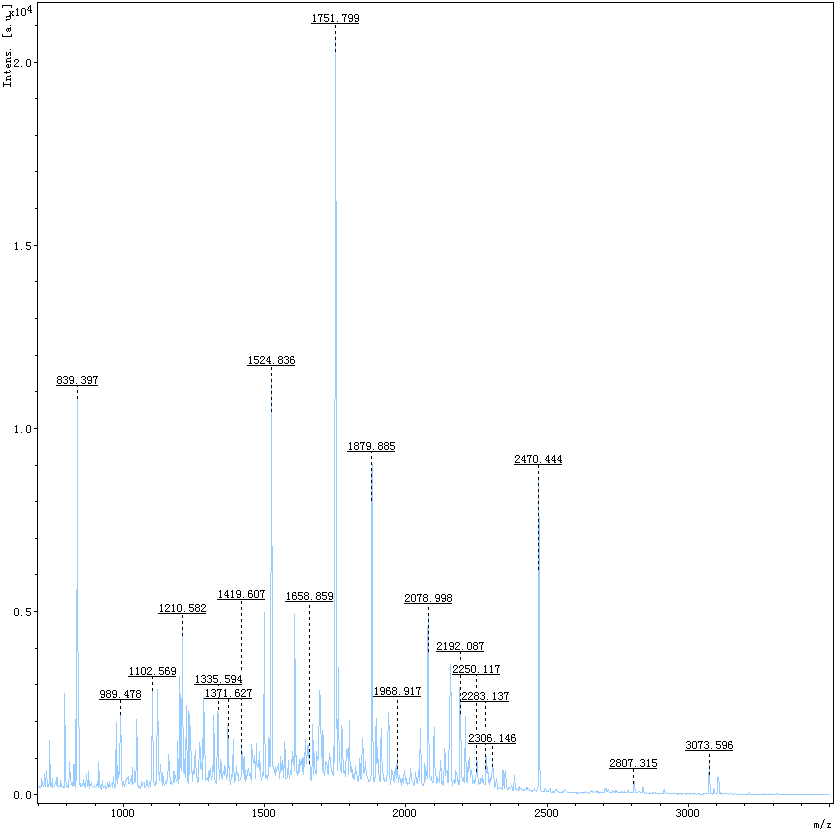


Spot 44


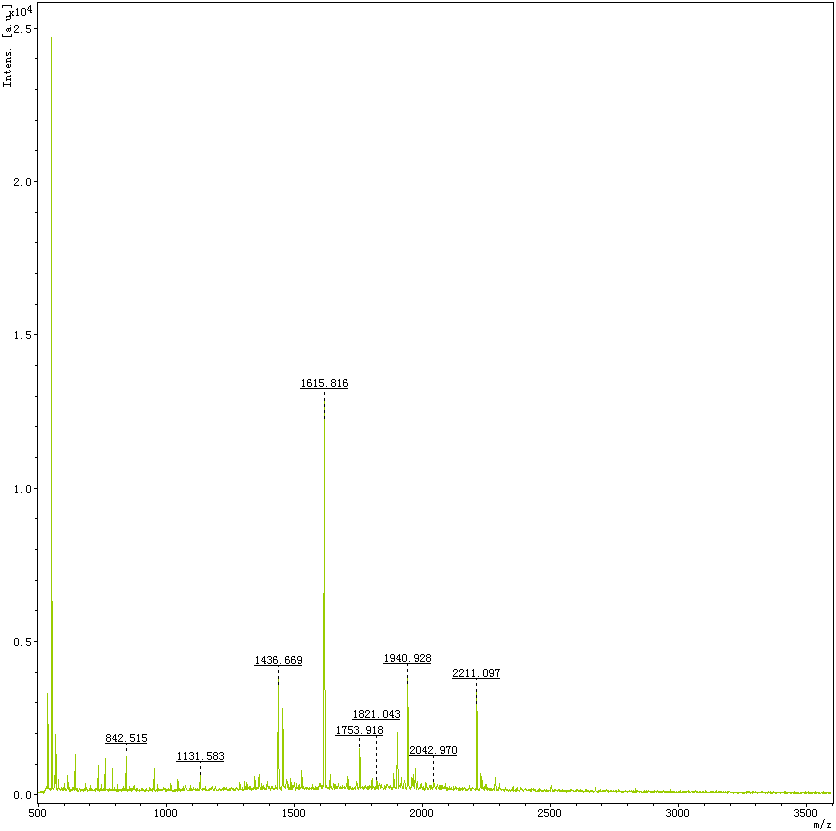


Spot 45


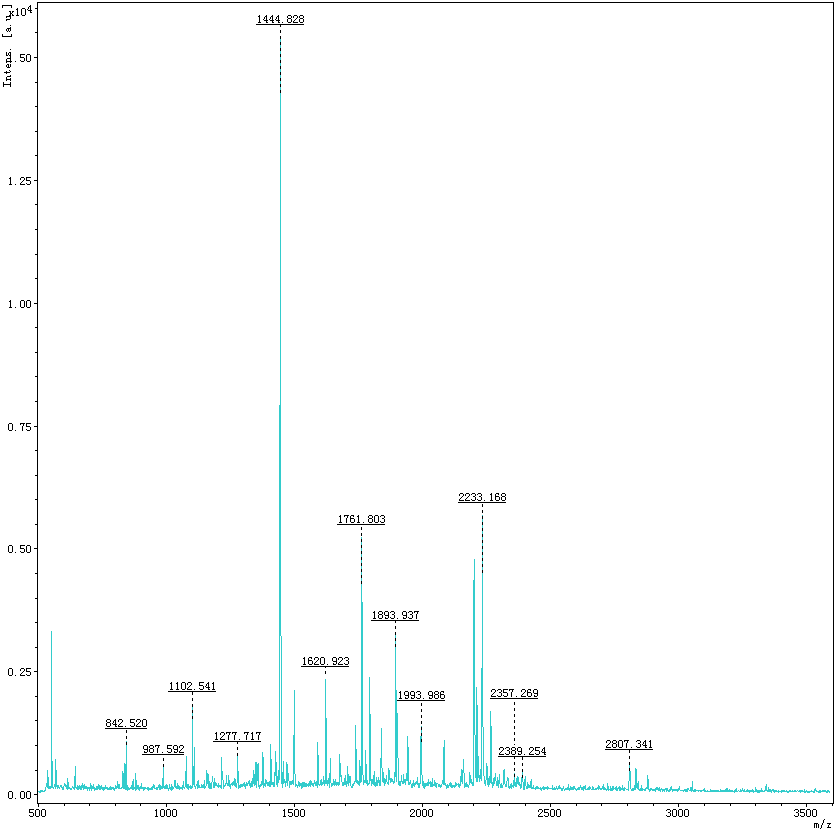


Spot 46


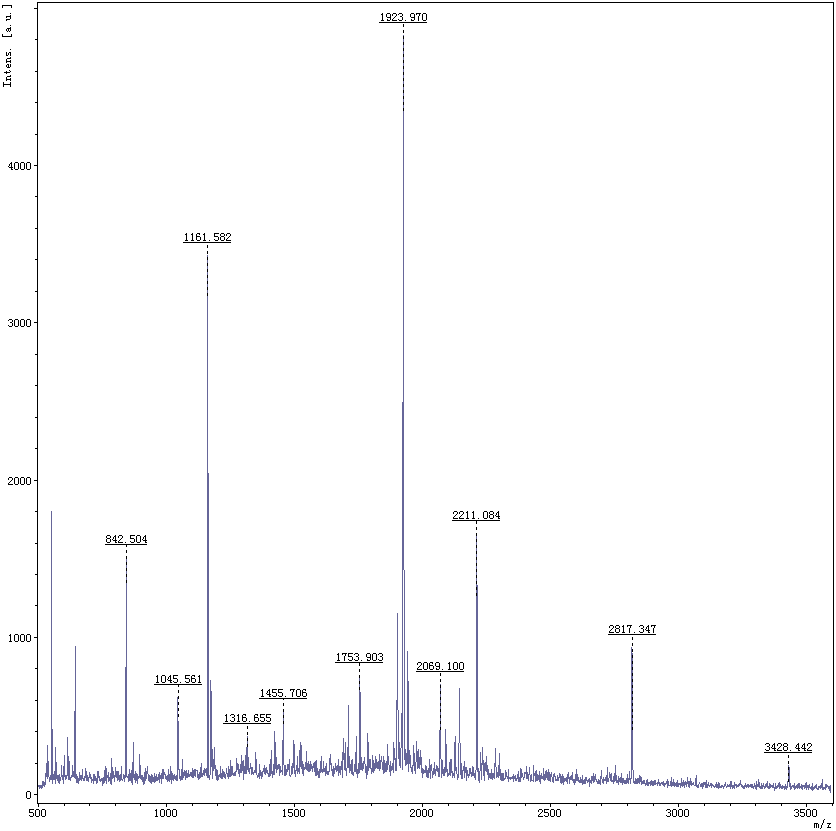


Spot 47


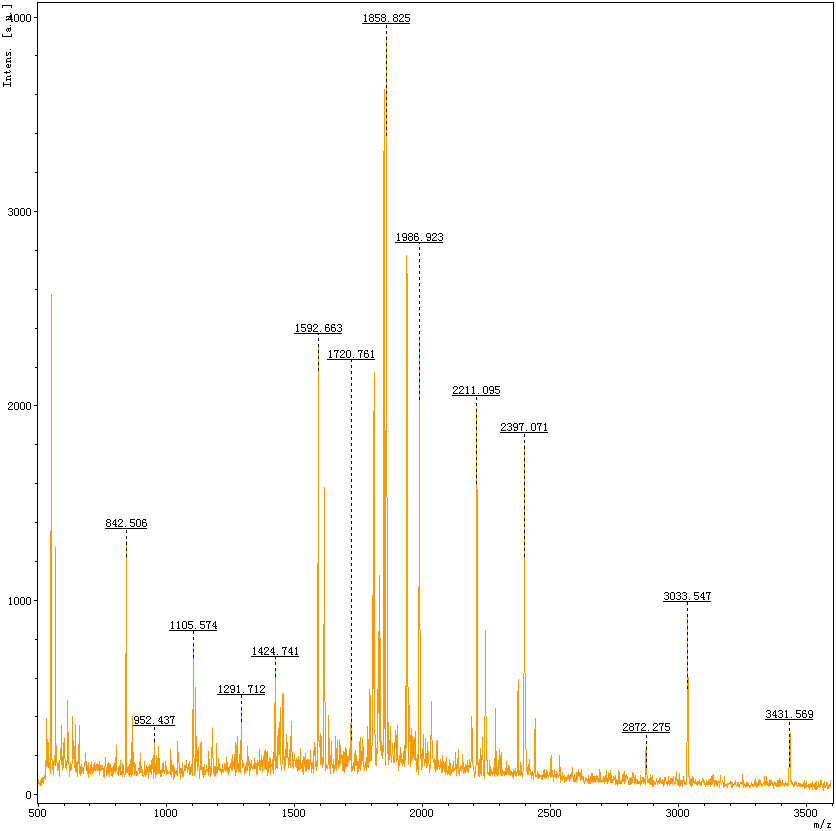


Spot 48


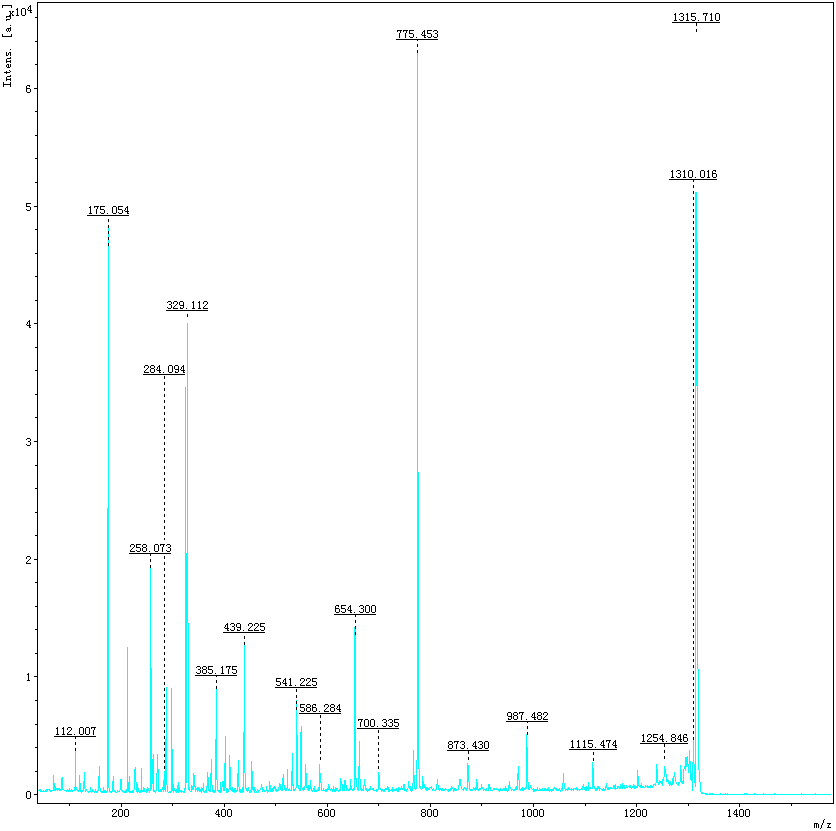


Spot 49


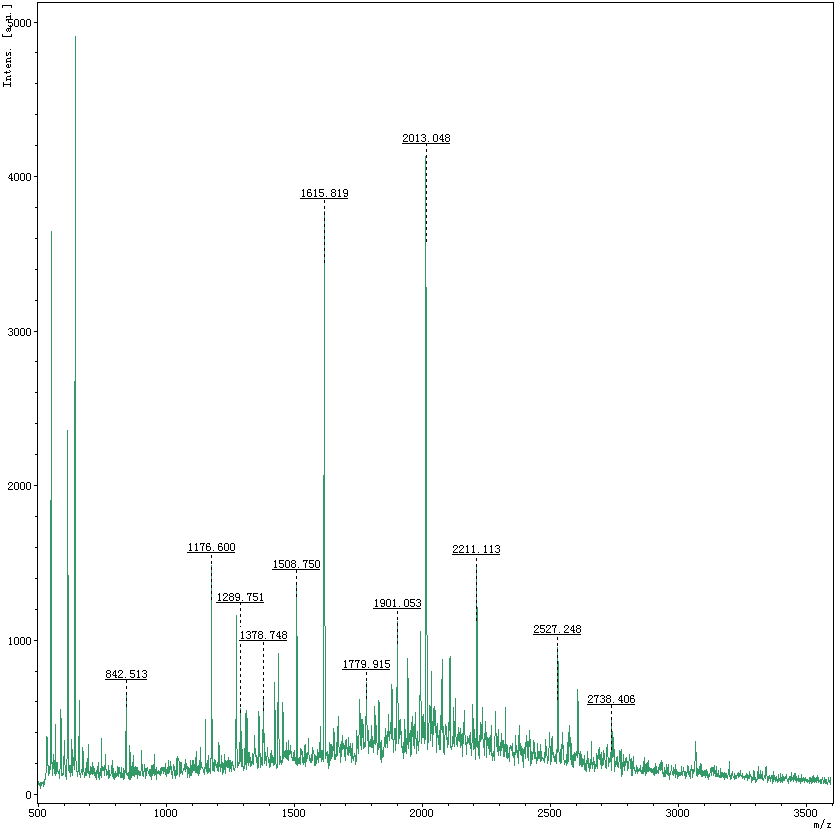


Spot 50


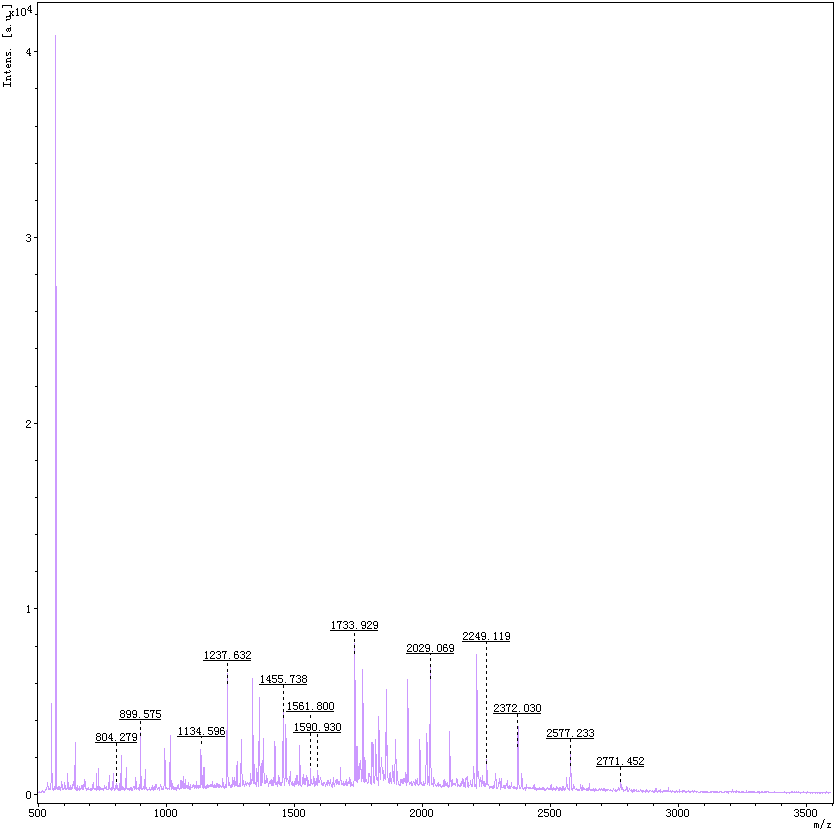


Spot 51


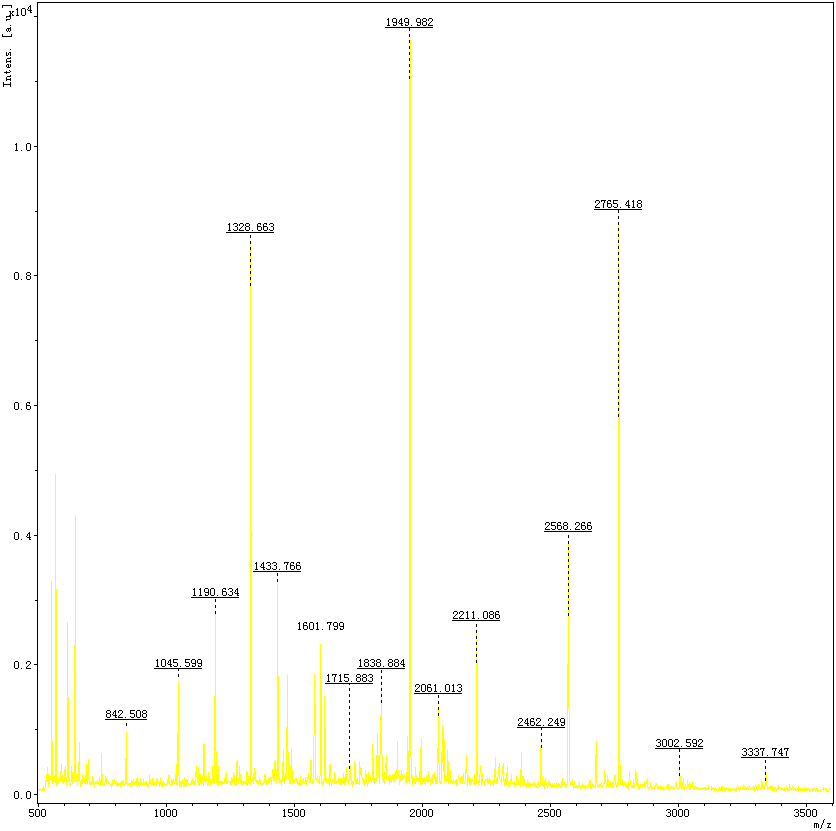


Spot 52


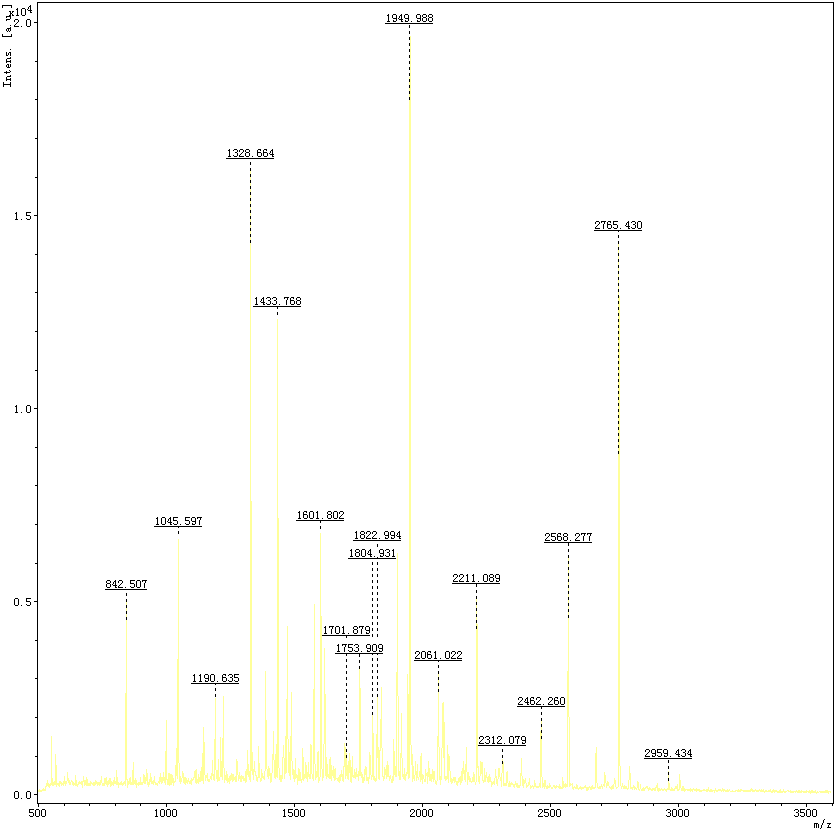


Spot 53


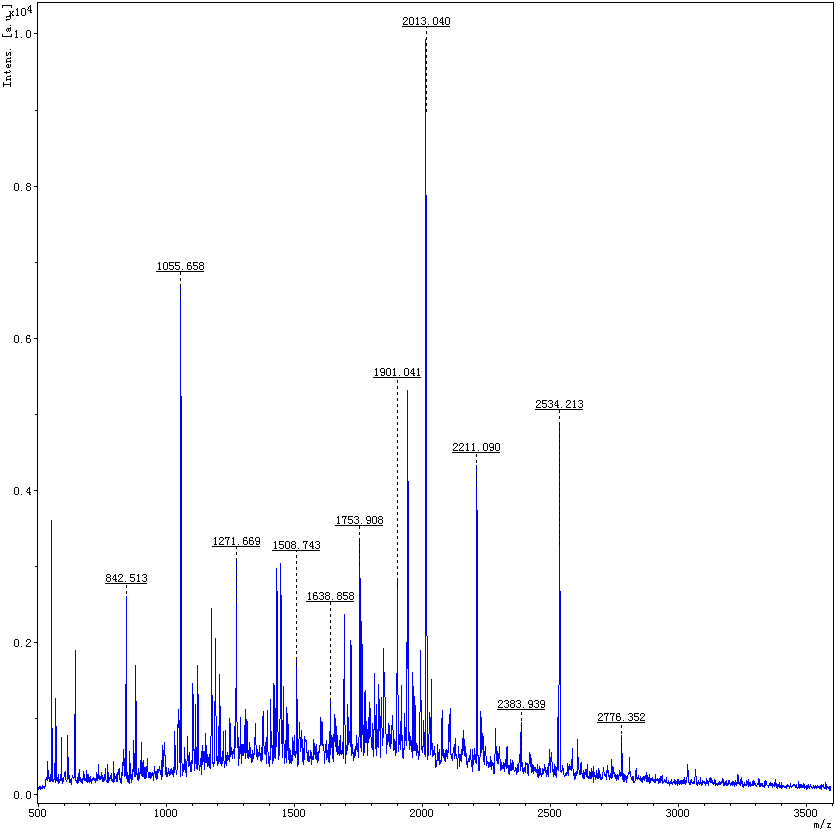


Spot 54


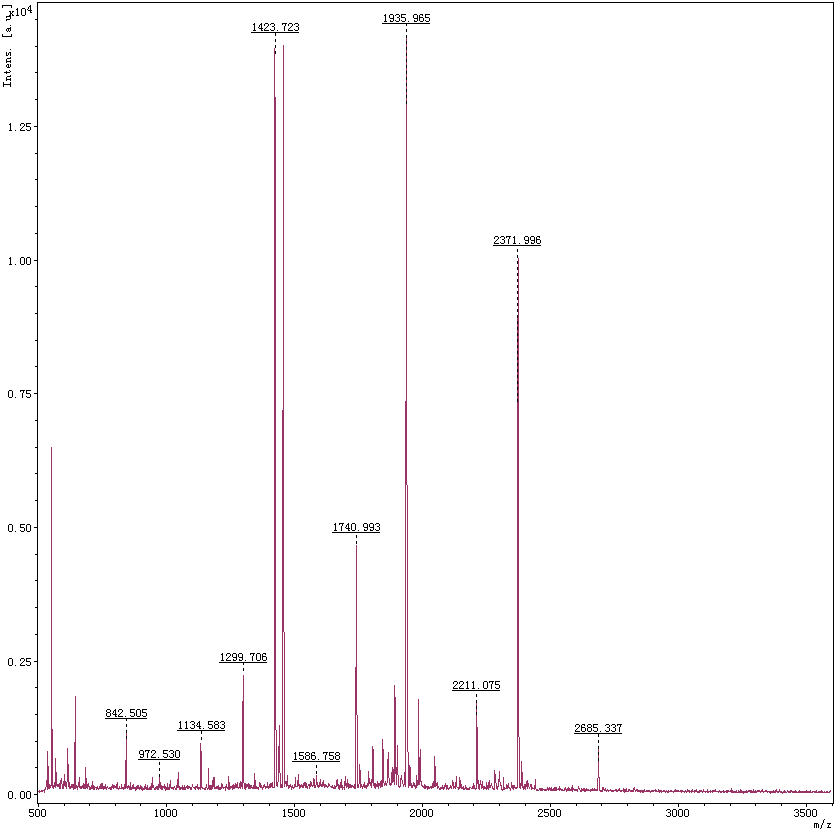


Spot 55


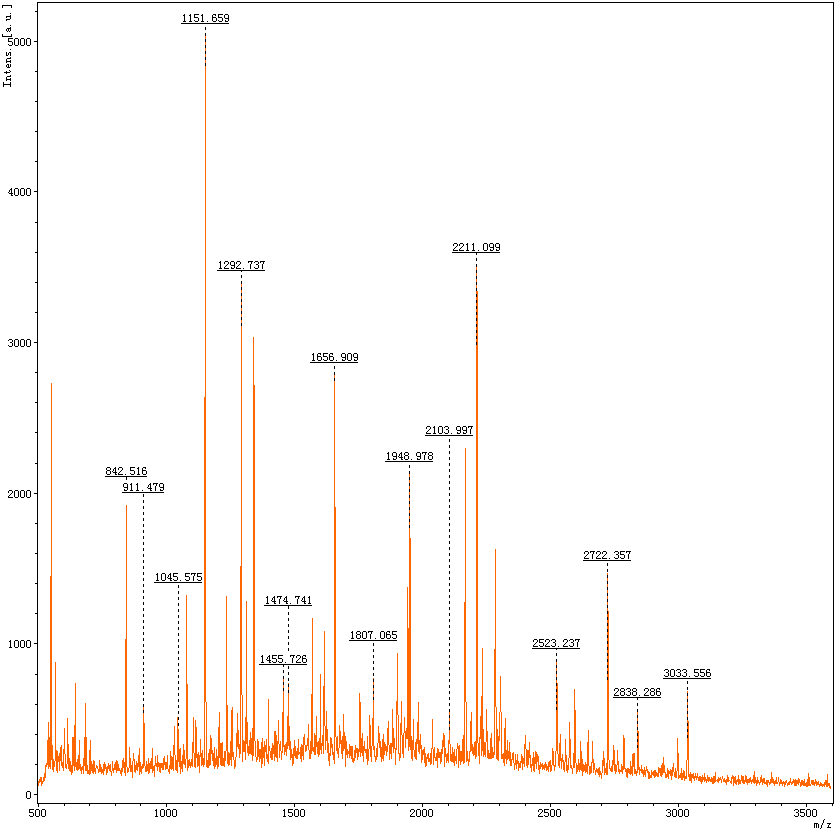


Spot 56


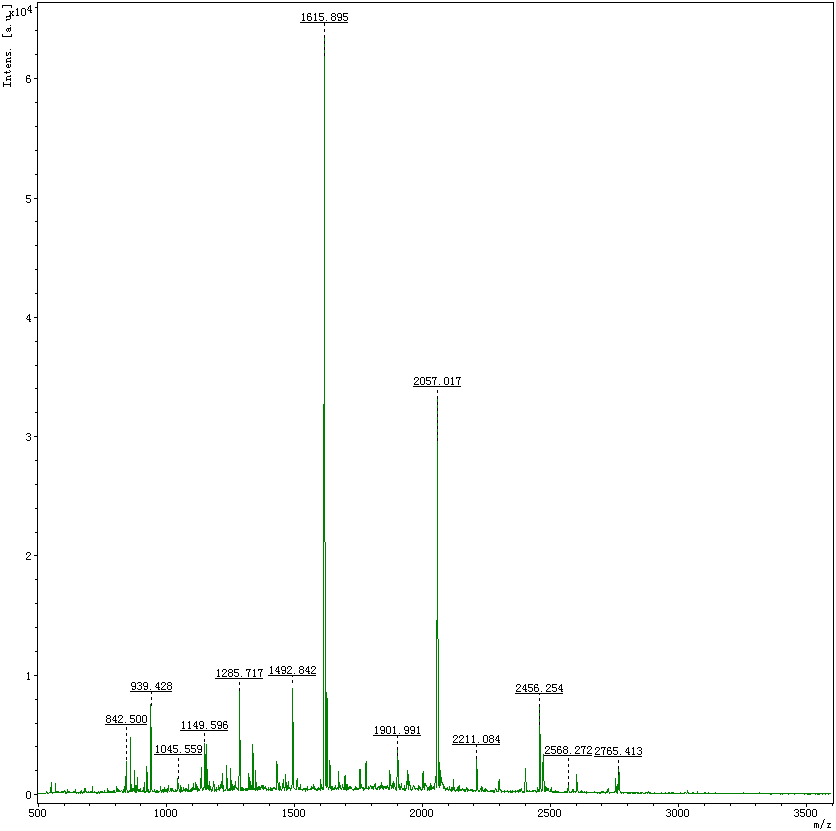


Spot 57


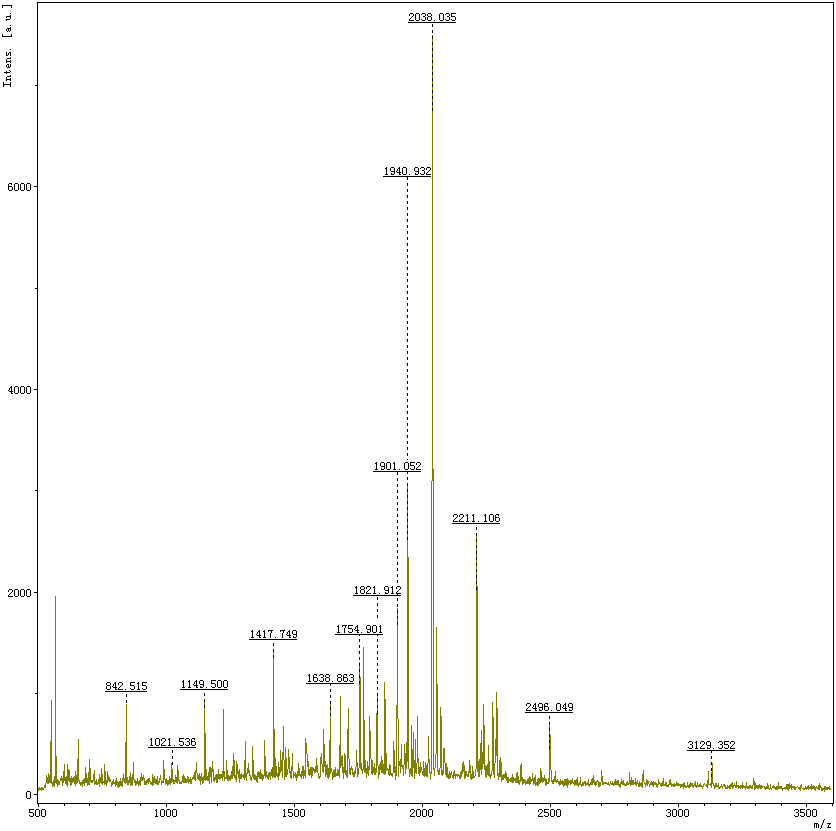


Spot 58


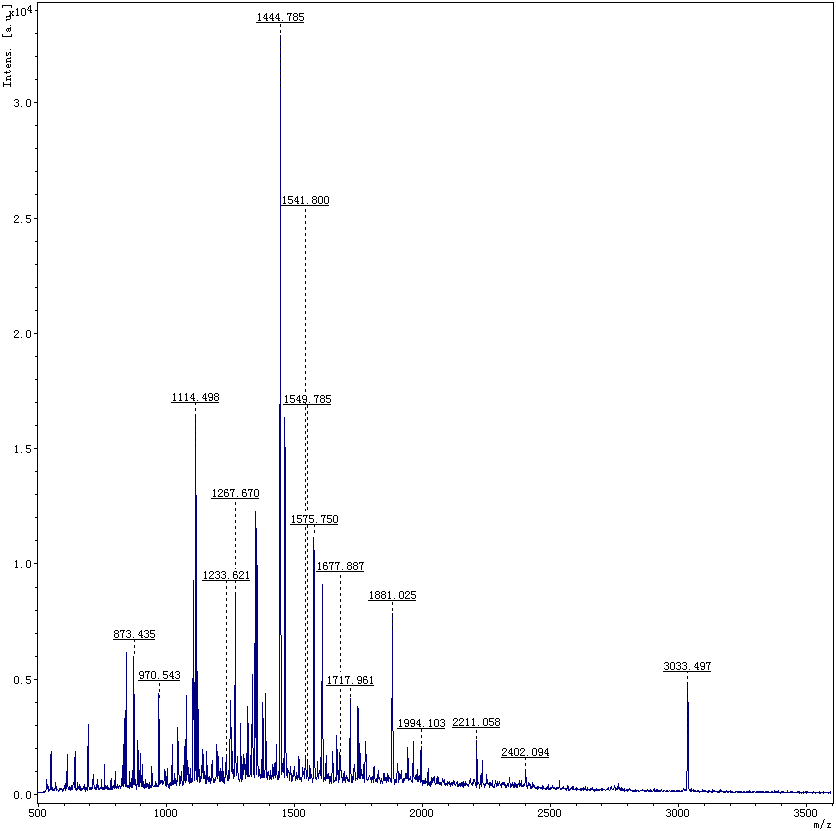


Spot 59


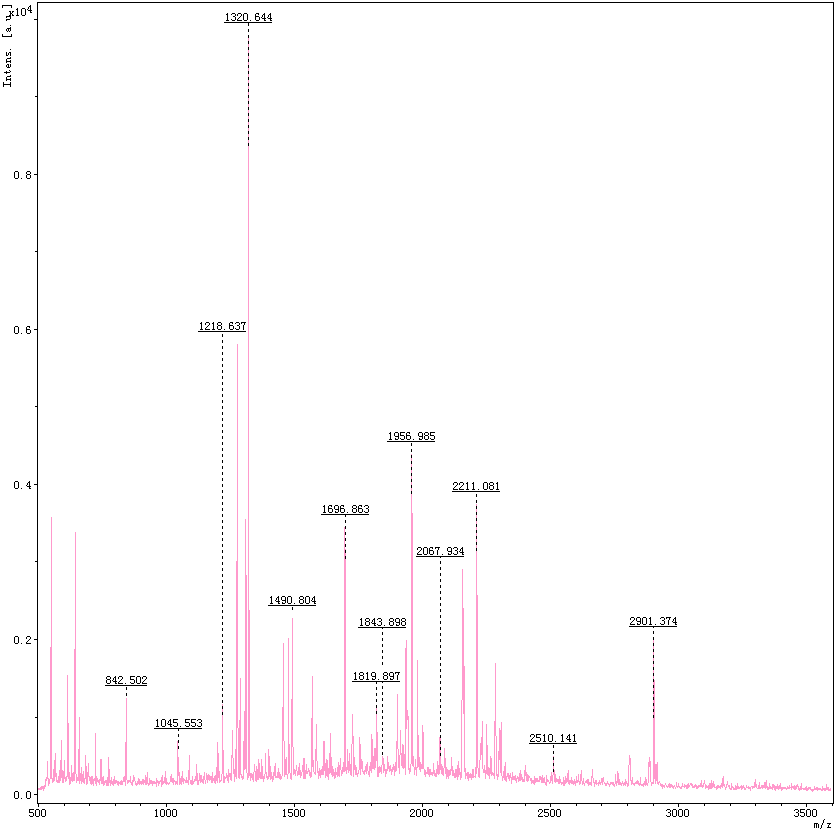


Spot 60


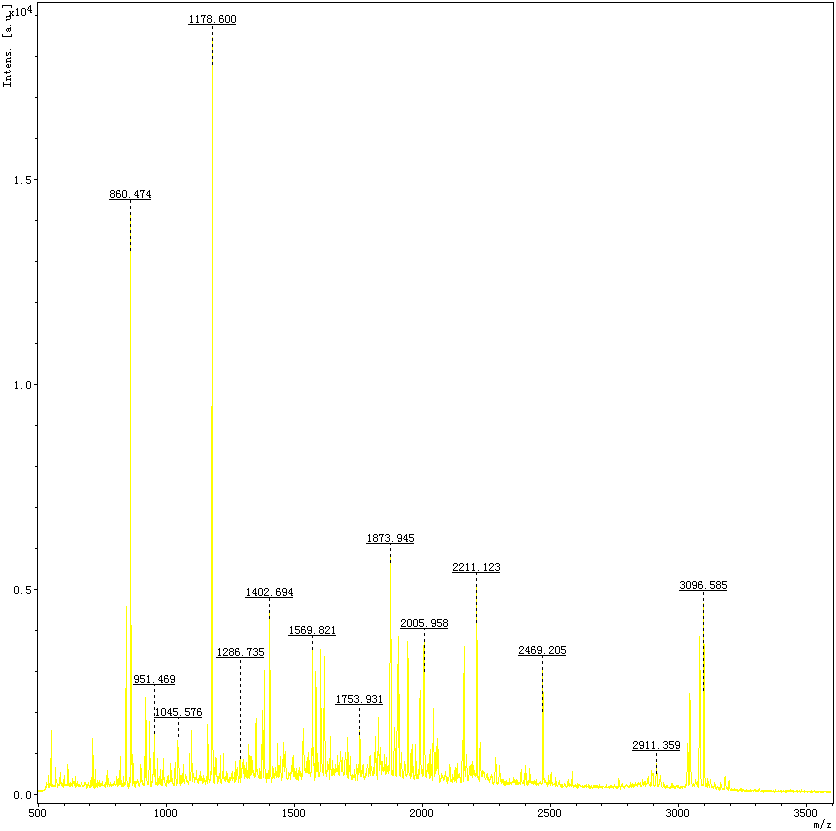


Spot 61


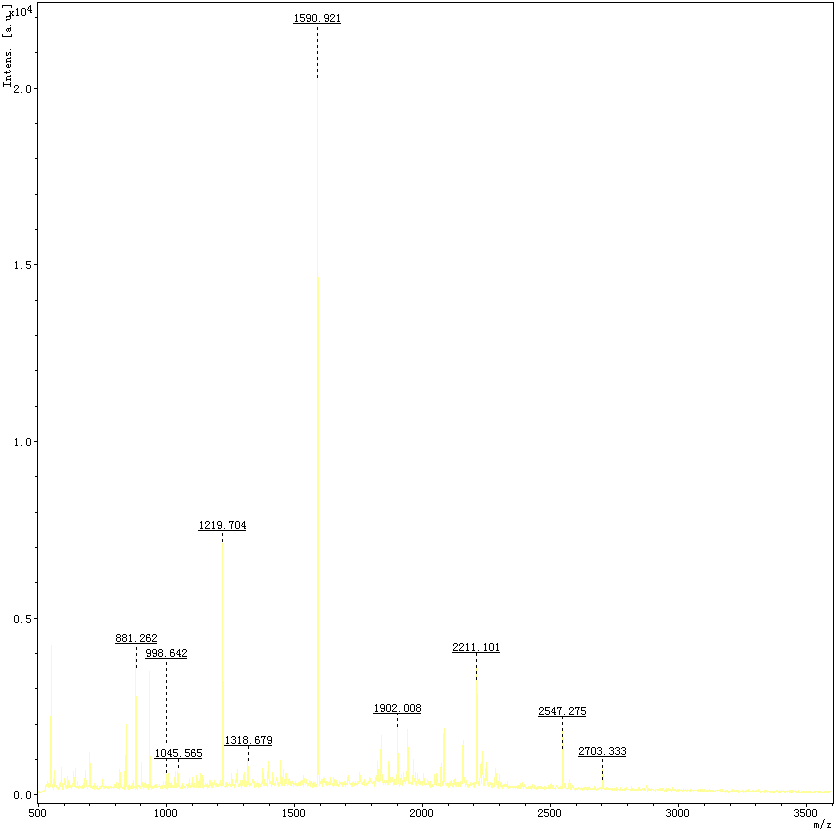


Spot 62


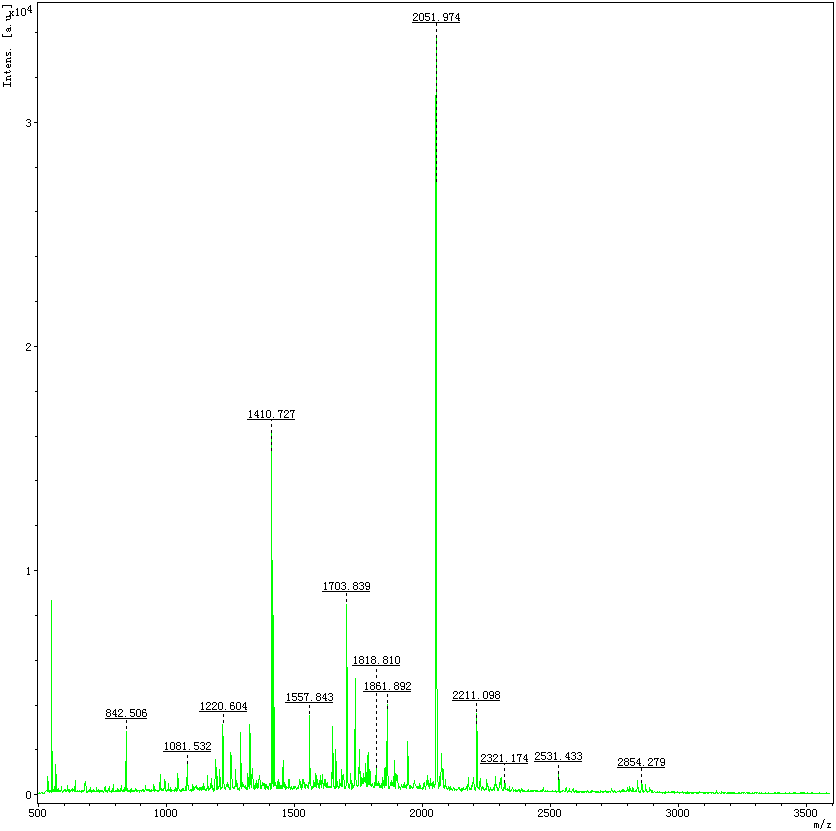


Spot 63


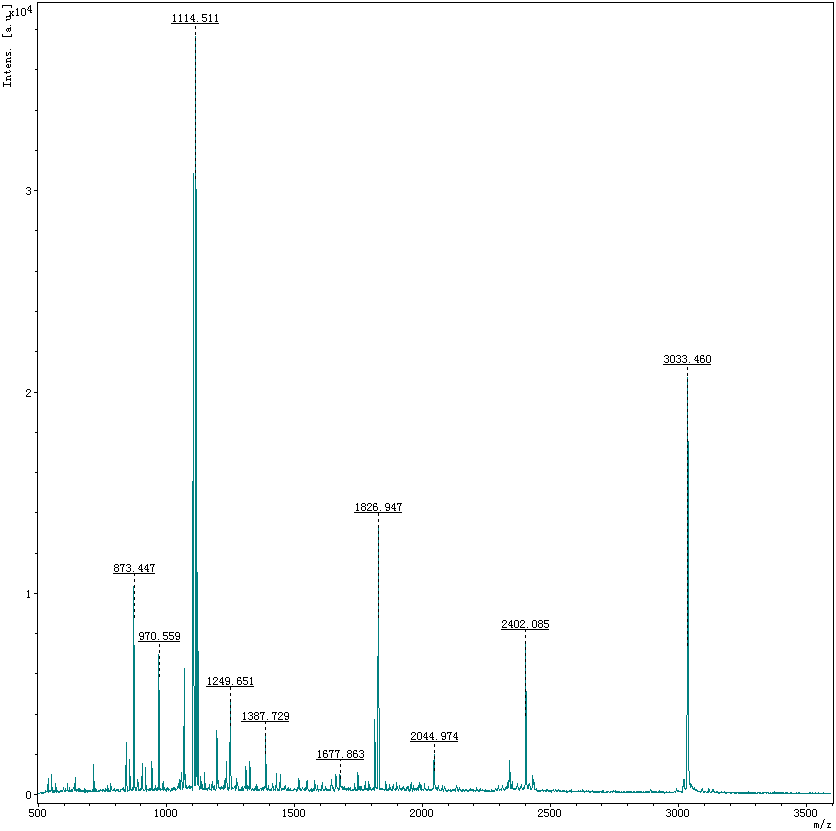


Spot 64


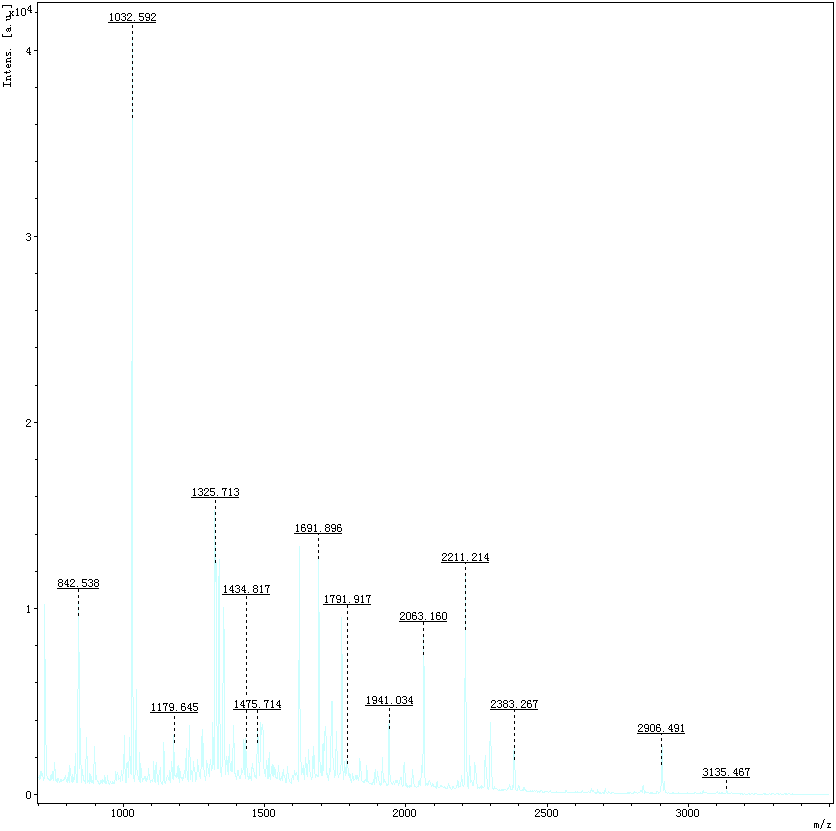


Spot 65


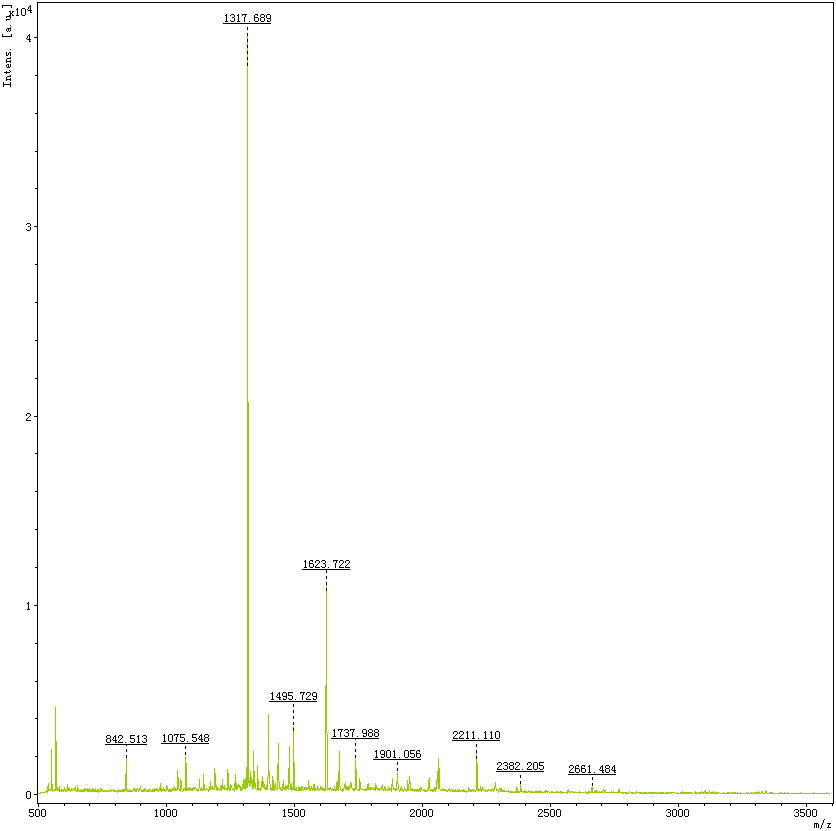


Spot 66


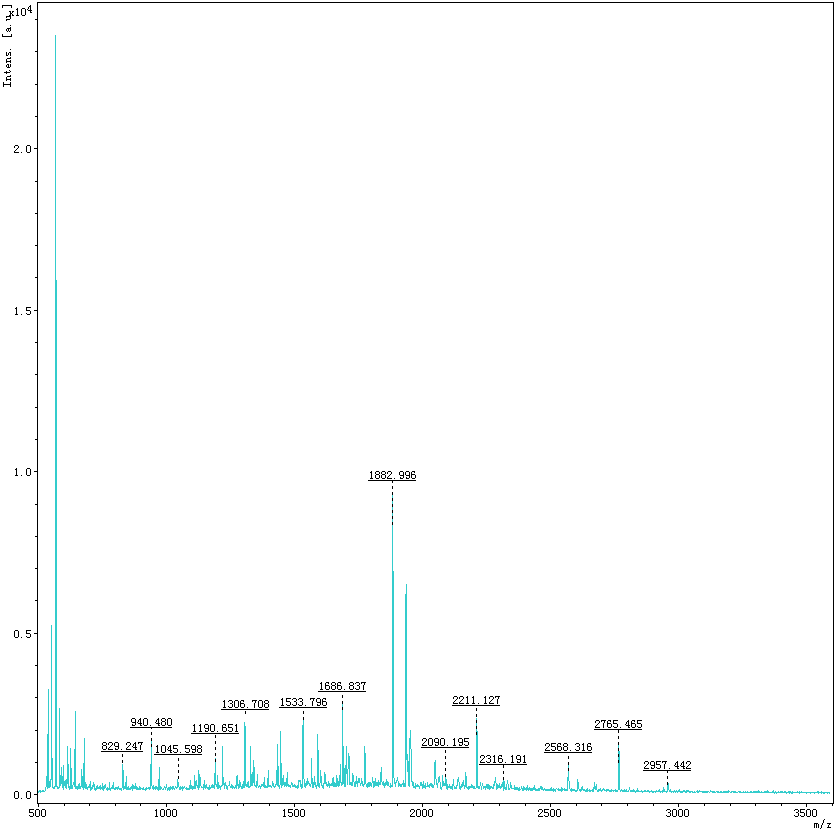


Spot 67


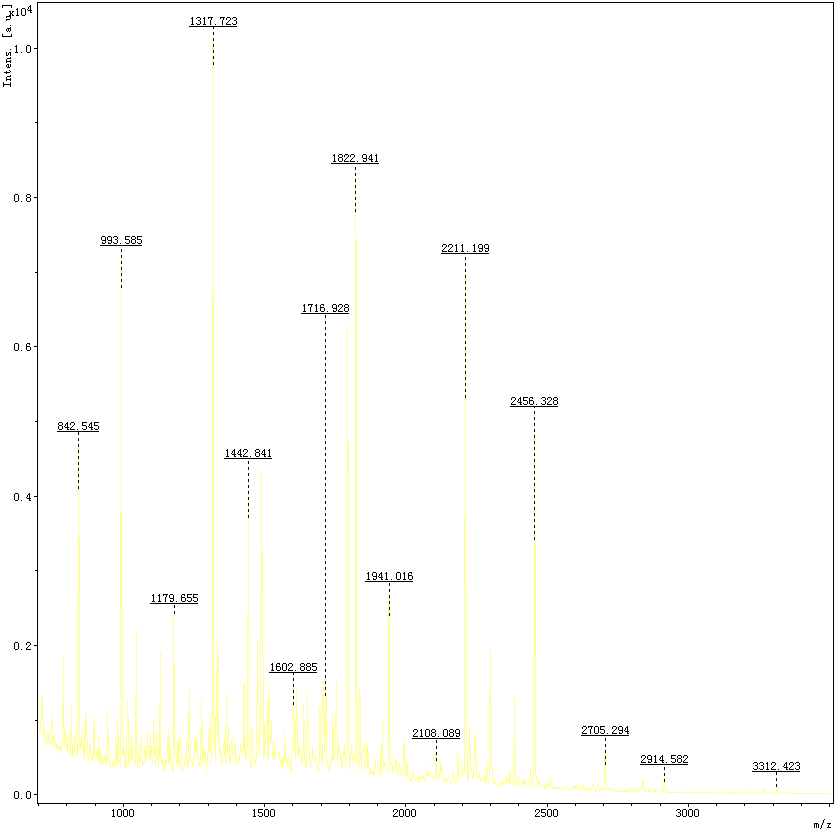


Spot 68


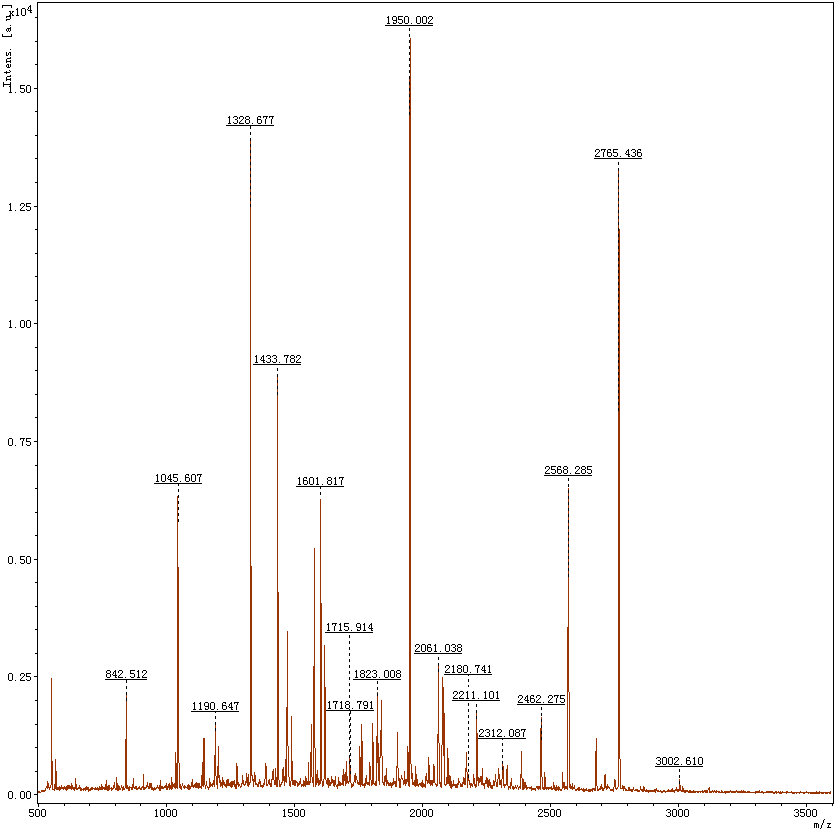


Spot 69


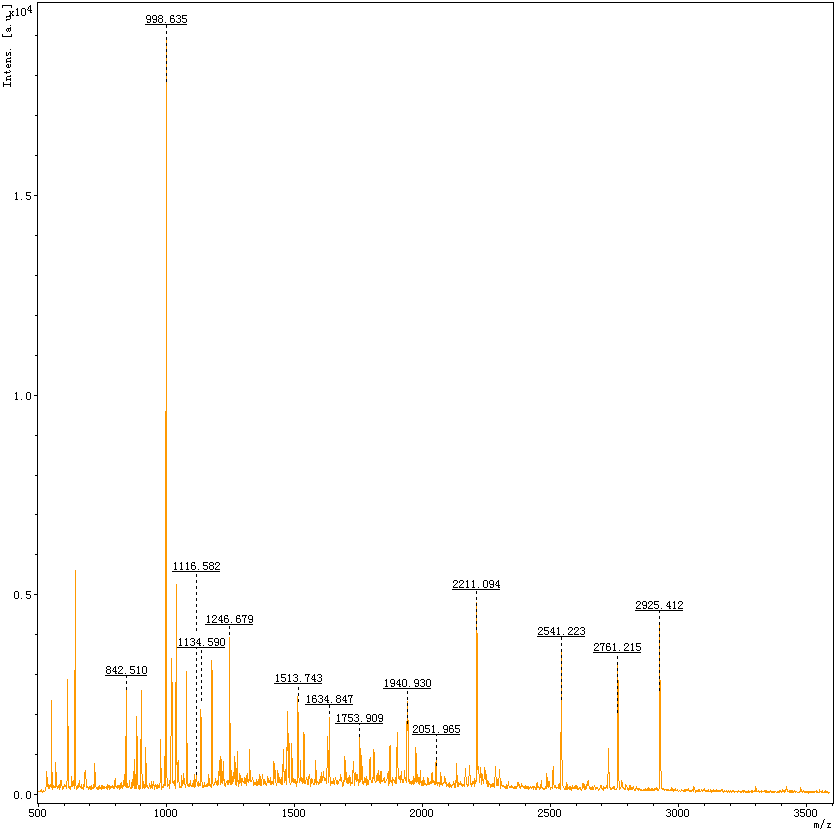


2.The nucleotide sequences of genes in this study.

| Spot No. | Gene name | fragment | | |
| --- | --- | --- | --- | --- |
| 4 | chlorophyll a/b binding protein precursor | TGATGGGTGCAGTTGAGGGTTACAGAATTGCTGGTGGGCCATTGGGTGAGGTTGTTGACCCACTTTACCCTGGTGGCAGCTTCGACCCATTGGGCCTTGCTGATGACCCAGAGGCTTTTGCTGAGTTGAAAGTGAAGGAGCTTAAAAATGGAAGACTAGCTATGTTCTCTATGTTTGGATTCTTTGTTCAAGCCATTGTGACCGGAAAGGGGCCTTTGGAAAACCTTGCTGACCACCTTGCTGACCCTGTTGCCAACAACGCCTGGTCCTACGCTACAAACTTCGTCCC | | |
| 6 | ACC oxidase 1 | ACTGATGGAACCCGAATGTCGATAGCCTCATTTTACAACCCAGGAAGTGATGCTGTGATCTATCCTGCACCAGAACTAGTAAACAAGGAAGCAGAAAAAAGCAGTATGTACCCGAAGTTTGTGTTTGAGGACTACATGAAGCTCTATGCTGAAGTGAAGTTTCAGGCAAAGGAGCCTAGATTTGAAGCTTTTAAGACCATGCAGGAGGCAGTCAGCGTCAGCCCCATTGCGACGGCTTGAGTGAAGAA | | |
| 13 | 26S protease regulatory subunit 6B homolog | ACTTCTTCGTCCTGGTAGGCTTGACCGCAAGATTGAATTCCCTTTGCCTGATAGACGACAAAAAAGGCTTGTGTTTCAGGTTTGCACTGCTAAAATGAACTTAAGTGACGAAGTTGACTTGGAAGATTACGTCTCTCGACCTGACAAGATCAGTGCTGCTGAGATCACAGCCATCTGTCAAGAAGCAGGGAT | | |
| 17 | GDP-mannose 3,5-epimerase | GGTTCTTCTATGCGTCAAGTGCTTGCATTTACCCTGAGTTTAAACAGTTGGAGACCAATGTGAGCTTGAAGGAAGCTGATGCGTGGCCTGCTGAGCCTCAAGATGCTTACGGTTTGGAGAAACTAGCAACAGAGGAGTTGTGCAGGCACTATACCAAAGACTTTGGAATCGAGTGCAGAAGTGGTCGTTTTCACAACATCTATGGACCTTTTGGC | | |
| 18 | Phosphoglycerate kinase | GGTCCTGAGGTTGAGAAACTGGTTGCTGAGATCCCTGAAGGGGGTGTTTTGCTCTTAGAAAACGTGAGGTTTTACAAAGAGGAAGAGAAGAACGACCCTGAGTTCGCTAAGAAGTTGGCATCACTAGCAGACTTGTACGTCAATGATGCTTTTGGCACTGCACATAGGGCCCATGCGTCTACTGAAGGTGTTGCCAA | | |
| 23 | xyloglucanendotransglucosylase | CAAGCACCCTTTTACGCATACTACAAGGACTTTGACATTGAAGGCTGCACCATGCCTGGTCCCAACAGTTGTAGCTCTAACCCATCCAATTGGTGGGAAGGTGCCACCTATCAACACCTGGACTCTGTTGCCGCCCACCGTTACCGGTGGGTCCGCGTGAACCACATGGTTTATGATTATTGCACAGACAAAATTCGGAACCCTGTGACCCCACCAGAATG | | |
|  |  |  | | |
|  |  |  | | |
| Spot No. | Gene name | | fragment | |
| 28 | NAD+ dependent isocitrate dehydrogenase subunit 2 | | | AGATTGCCAAAAGTTATCCCGACATCAAATACAATGAAATTATTGTGGACAACTGCTGCATGCAACTTGTTTCAAAACCCGAGCAATTTGATGTCATGGTGACACCGAACCTATACGGCAACCTAATTTCAAACACTGCTGCTGGTATTGCTGGTGGCACAGGTGTAATGCCAGGAGGAAATGT |
| 31 | RuBisCO large subunit | | | AACGCCAGGGTTTTGATTACAGATCAAAAGATATCTTCAATAAAGGAGATTATTCCACTATTGGAAAAAACAACTCAATTAAGAGCTCCTTTGCTGATTATTGCTGAGGATGTCAGTGGCGAGGCTTTGGCTACTCTTGTTGTAAACAAGCTTCGTGGTATCCTTAATGTTGCTGCCATTAAAGCACCTGGTTTTGGTGAAAGGAGAAAGGCTTTGCTTCAAGATATTGCCATCGTGACAGGAGCCGAGTAT |
| 33 | temperature-induced lipocalin | | | TGGACTGATGGTAAGAGGGGATATATTGAAGGAACTGCTTATAAGGCTGATCCTAAAAGTGATGAGGCTAAATTGAAGGTTAAGTTTTGGGTGCCACCTTTTTTGCCTATCATTCCTGTTACTGGTG |
| 35 | fructose-bisphosphatealdolase | | | AGAACCTTCGCCAACCATCAATCTCCGTAGTTCGGTTCAGCACCAACGCACCCGCTGTACTAACCGTCCGTGCTTCATATGCCGACGAGCTTGTTAAAACCGCTAAAACCATTGCATCACCTGGGAGAGGAATCCTGGCTATGGATGAGTCGAATGCTACATGTGGAAAGCGGTTAGATTCTATTGGTCTTGAGAACACTGAGGCTAACCGACAAGCTTACCGGACCCTACTTGTTACTCCACCAGGTCT |
| 36 | glutamate dehydrogenase | | | TTCGGAAATGTTGGTTCGTGGGCTGCTCAACTTATTCATGAAGCTGGTGGAAAAGTTGTAGCAGTGAGTGATATAACGGGAGCTATAAAGAATAGTAATGGACTTGACATCCCCAACCTGCTAAAACAT |
| 39 | aldolase | | | GACTACATTGACAAAGTTGGGGAGCTTGCCAAAAAGCATGGGTTGAAGCTTCATATCGATGGGGCGCGGATTTTTAATGCCGCTGTTGCTCTTGATGTTCCTGTTCATAGGCTTGTACAAGCTGCTGACTCTGTTTCGGTATGCTTGTCGAAAGGGCTTGGTGCACCAGTTGGGAGTGTCATTGTTGGAACAAAAACCTTCATTGATAGGGCAAGAATTCTTCGAAAAACCTTAGGGGGTGGAATGAGACAGGTCGGCTT |
| Spot No. | Gene name | | fragment | |
| 45 | Auxin-induced protein PCNT115 | AAGAATCAAGTTGGGTTCACAGGGTTTGGAAGTATCAGCACAAGGTTTGGGTTGTATGGGCATGTCTGCATACTATGGCCCACCAAAACCCGAAGCCGACATGATCAACCTCATCCATCAGGCCATTGCTGCTGGTGTTACACTTCTTGACACTTCGGATGTCTATGGGCCCCATACAAATGAAATCTTACTTGGAAAGGCTTTGAAGGG | | |
| 51 | ATP synthase beta subunit | TATGGTTAGGACTATTGCTATGGATGGTACCGAAGGCCTTGTTCGCGGTCAACGTGTTTTAAATACTGGCTCTCCCATCACTGTGCCTGTTGGTAGAGCTACACTTGGAAGGATTATTAATGTTATTGGAGAGCCTATTGATCATAGAGGCGATATTAAAACGGATCATTATTTGCCCATTCATAGAGAAGCTCCAGCTTTTGTTGAGCAAGCAACTGAACAACAAATTCTTGTTACTGGAATTAAGGTTGTTGATCTCCTTGCACCGTATCAAAGAGGAGGAAAGA | | |
| 54 | Actin-depolymerizing factor 2 | GCATCTGGAATGGCTGTTGACGATGAATGCAAGTTAAAGTTTTTGGACCTAAAAGCAAAGAGAAACTACCGATATATTACTTTCAAGATCGAGGAGCAAAAAGTGATGATTGACAAGATTGGTGGCCCTAACGAAACCTACGAGGATTTCACAAACTCTCTTCCTGGCAATGAATGCCGCTATGCTGTCTTTGATTTTGAC | | |
| 56 | carbonic anhydrase isoform 1 | GTATTTGCCTGCTCGGATTCTCGAGTTTGTCCGTCACACATCCTGGATTTCCAGCCAGGTGAAGCCTTTGTAGTCAGAAACATTGCTAACATGGTTCCTCCTTATGATACGGTTAAACACTCTGGAGCAGGGGCGGCTATTGAATATGCAGTAGTGCACCTAAAGGTGGAGAATATTGTAGTTATTGGACATAGCTGTTGTGGTGGAATTAAAGGACTCATGTCCATCCCTGAAGATGGAAGTACTGCCTCTGACTTCATTGAACAATGGGTCAAAATCGGGTTGCCTG | | |
| 59 | magnesium-protoporphyrin IX methyltransferase | TGGCTTGGGACTTGGGGTTCTTGACCAAAGCACGAAAATACACCTTTTTCAAGCCAAAATTCATATTTTACGCCACATATTTGTCTGAAAAGATCGGGTATTGGAGATACATCACCATATACAGACACCTAAAGGCGAACCCCGAGTACCAATGTTACCCAATTTTCAAGTACTTTGAGAACTGGTGCCAAGACGAGAACAGACACGGTGACTTTTTCTCTGCACTCATGAAGGCACAGCCACAGTTTCTCAATGACTGGAAGGCTAAGCTTTGGGCTCGTTTCTTCTGCTTATCTGTG | | |
|  |  |  | | |
| Spot No. | Gene name | | fragment | |
| 61 | Malate dehydrogenase | ATCCCTGCTTCTTGACAGATGGCTGTGATCTCAGCAGCACTGATCTTGTCAGGTCGAGAGACGTAATCTTCCAAGTCAACTTCGTCACTTAAATTCATTTTAGCAGTGCAAACCTGAAACACAAGCCTTTTTTGTCGTCTATCAGGCAAAGGAAACTCAATCTTTCGGTCAAGCCTACCAGGACGAAGAAGT | | |
| 62 | Acyl-[acyl-carrier-protein] desaturase | GATGGGCGTGATGACAACCTCTTTGAACATTTCTCAGCTGTTGCACAAAGACTCGGTGTCTATACTGCCAAAGACTATGCAGATATTCTTGAATTTCTGGTGGGTCGGTGGAAAGTGGCTGACTTAACCGGGCTATCTGGTGAAGGGCGCAAGGCCCAAGAGTATGTTTGTGGGTTGCCA | | |
| GAPDH | Reference gene | CTGCTTCTTTCAACATCATTCCCAGCAGCACTGGAGCTGCCAAGGCTGTTGGCAAAGTGTTGCCTGCACTTAACGGTAAATTGACCGGAATGGCCTTCCGTGTACCAACCGTTGATGTCTCAGTTGTTGACTTGACTGTGAGACTTGAGAAGAAGGCTACCTATGAGCAG | | |
